# The WHO estimates of excess mortality associated with the COVID-19 pandemic

**DOI:** 10.1038/s41586-022-05522-2

**Published:** 2022-12-14

**Authors:** William Msemburi, Ariel Karlinsky, Victoria Knutson, Serge Aleshin-Guendel, Somnath Chatterji, Jon Wakefield

**Affiliations:** 1 World Health Organization, Geneva, Switzerland; 2 Hebrew University, Jerusalem, Israel; 3 Department of Biostatistics, University of Washington, Seattle, WA USA; 4 Department of Statistics, University of Washington, Seattle, WA USA

**Keywords:** Epidemiology, Social sciences

## Abstract

The World Health Organization has a mandate to compile and disseminate statistics on mortality, and we have been tracking the progression of the COVID-19 pandemic since the beginning of 2020^1^. Reported statistics on COVID-19 mortality are problematic for many countries owing to variations in testing access, differential diagnostic capacity and inconsistent certification of COVID-19 as cause of death. Beyond what is directly attributable to it, the pandemic has caused extensive collateral damage that has led to losses of lives and livelihoods. Here we report a comprehensive and consistent measurement of the impact of the COVID-19 pandemic by estimating excess deaths, by month, for 2020 and 2021. We predict the pandemic period all-cause deaths in locations lacking complete reported data using an overdispersed Poisson count framework that applies Bayesian inference techniques to quantify uncertainty. We estimate 14.83 million excess deaths globally, 2.74 times more deaths than the 5.42 million reported as due to COVID-19 for the period. There are wide variations in the excess death estimates across the six World Health Organization regions. We describe the data and methods used to generate these estimates and highlight the need for better reporting where gaps persist. We discuss various summary measures, and the hazards of ranking countries’ epidemic responses.

## Abstract (web summary)

Msemburi et al. describe how the World Health Organization has estimated the excess mortality associated with the COVID-19 pandemic, by month and for 2020 and 2021, and analyse their estimates across the WHO member states, with 14.83 million global excess deaths estimated.

## Main

The COVID-19 pandemic caught the world unprepared, and it has exacted a toll many would have considered inconceivable in the modern era before its emergence. As of 31 December 2021, more than 287 million confirmed cases of COVID-19 across the world had been reported to the World Health Organization (WHO) including 5.4 million deaths (https://covid19.who.int/).

From the documenting of initial COVID-19 cases in Wuhan, China in December of 2019 through to the WHO declaring it a pandemic in March of 2020^1^, accurately tracking COVID-19 and its impact has been riddled with challenges. An initial major challenge was developing the diagnostic tools to correctly identify the presence of the virus. A number of countries relied on pre-existing platforms to achieve this and they quickly adapted and scaled up the available technologies to allow for COVID-19 testing. However, many countries lacked such capacity. In addition, countries have differed in their application of standards for the certification of COVID-19 as the underlying cause-of-death^2^. This has caused both country-level and worldwide assessment of the spread and impact of the pandemic to be incomplete. An estimate of the excess mortality associated with the COVID-19 pandemic is therefore a better measure of the overall impact of the crisis.

Excess mortality is defined as, “The difference in the total number of deaths in a crisis compared to those expected under normal conditions”^3^. Excess mortality accounts for both the total number of deaths directly attributed to the virus and those resulting from the indirect impact, such as disruption to essential health services or travel disruptions^4^. Excess mortality is a well established concept dating back centuries^5^, and has been used extensively to estimate the toll of past health crises and pandemics such as the 1918 ‘Spanish Flu’^6^. The measure overcomes the variation among countries in reporting and testing and the misclassification of the cause of death on death certificates^7^ and requires only information on the total number of deaths during the health crisis, and before, to establish the expected number of deaths.

Unfortunately, excess mortality cannot be directly estimated for all countries owing to many not having the requisite all-cause mortality (ACM) data. The WHO usually receives routine mortality data on an annual basis following the year of death or after an even longer lag. Civil registration and vital statistics (CRVS) systems differ across countries with varying completeness, timeliness and quality control measures for compiling unit record cause of death numbers into aggregates identified by cause, age, sex, place and period of death^8–11^. Moreover, differential reporting coverage, the absence of electronic surveillance systems in some locations and limited investments in CRVS systems has resulted in many nations lacking the structures necessary to provide good-quality routine data, even before the onset of the pandemic. Correspondingly, they lack the capacity and data required to monitor ACM during this unprecedented pandemic. This results in numerous countries being unable to contribute to the centralized systematic mortality surveillance that would be necessary for the WHO to measure global-, regional- and country-level excess mortality. Acknowledging these data gaps, a model-based framework, relying on ACM information from countries for which data exist and other relevant factors, has been developed by the WHO. The purpose of this framework is to estimate country, regional and global excess deaths from 1 January 2020 to 31 December 2021 on a monthly timescale.

## Process, methods and data

Detailed descriptions of the process followed, data used and methods applied to generate the estimates of excess mortality within this paper are provided in the Methods and summarized here. The WHO, in collaboration with the United Nations Department of Economic and Social Affairs, assembled a technical advisory group to develop guidance on how to best estimate excess mortality in light of extensive data gaps. Preliminary estimates were generated according to the advice of this group and, following standard WHO procedures, a consultative process was initiated with member states who assessed the input data sources, methods and results, and provided feedback which was in turn used to update the estimates. Ideally, we would have ACM data for all countries and for all months. The reality is that such monthly national data are available for only 100 countries (52%), with other countries having annual data, subnational data or no data. For the latter three cases, we predict the monthly data within a Poisson count model framework, as detailed in the Methods.

The ACM data used for our modelling come from various sources including nominated country focal points and public-facing databases, and can be characterized according to three criteria: whether the data are nationally representative, what proportion of the two-year pandemic period they cover and the time periods to which the data are aggregated.

By region, the monthly data that are available consist of only 6 out of the 47 countries in the Africa region (13%), 23 out of the 35 countries from the region of the Americas (66%), 9 out of the 21 countries from the Eastern Mediterranean region (43%), 51 out of the 53 countries in the European region (96%), 2 out of 11 countries in the South East Asia region (18%) and 9 out of 27 countries in the Western Pacific region (33%). There are 10 countries with subnational or annual data; 4 are from the region of the Americas, 1 from the European region, 3 from the South East Asia region and 2 from the Western Pacific region.

We use a Bayesian Poisson framework to estimate both the expected deaths (for all countries and all months) in the absence of the pandemic, and the ACM for those countries with no such data during the pandemic. In addition to the data on ACM, we gathered information on specific variables with spatiotemporal variations considered to be associated with changes in excess mortality over the course of the pandemic. These variables are chosen based on the strengths of the associations and availability across locations for the duration under study. We consider several that are assumed to change by month such as the COVID-19 death rate, the COVID-19 test positivity rate, aggregate containment measures (combining lockdown restrictions and closures) and average national temperature, together with others that are fixed over the period of study including a high-income country binary indicator, historic cardiovascular disease death rates and historic diabetes prevalence rates. A log-linear regression model on these variables, also within the Bayesian Poisson framework, is used to predict mortality levels in the locations without adequate reporting of mortality during the pandemic. For a handful of countries, instead of covariates, their subnational observed deaths are used to predict the national deaths using multinomial models that assume the relationships estimated between pre-pandemic subnational and national mortality persist into the pandemic. Finally, the reported or distributions of predicted deaths, conditional on data availability, together with the derived expected death distributions, are used to estimate monthly excess deaths in all locations for the years 2020 and 2021.

There are many different facets to the monthly time series of excess mortality that we may examine: (1) the raw excess counts, (2) the excess rate (say per 100,000 of population), (3) the P-score, which is the ratio of the excess to the expected and (4) the ratio of the excess to the reported COVID-19 deaths. Each of the four metrics are not known with certainty, even when we observe the total ACM over the complete pandemic, because the expected numbers are estimated. In general, between-region and between-country comparisons are difficult for many reasons, including the different age structures of the populations.

Raw excess counts directly show the basic human toll of the pandemic but are obviously critically dependent on the population size of the group (for example, country, region) over which we are calculating the excess. The excess rate adjusts for the population, to produce summaries that are more comparable across countries. It would be preferable to adjust for the age–sex population structure of each country, but unfortunately we do not have reliable excess counts by age and sex for all countries of the world.

Another method to compare countries’ excess deaths is to normalize the excess estimates by the expected number of deaths for the analysed period, expressed as a percentage. This measure is know as a P-score^12^. For example, if 100 deaths were expected to occur and the actual number of deaths was 140, excess deaths would be 40 and the P-score would be 40%. The P-score implicitly considers both the population size and the age structure. Two countries may have identical population sizes, but very different routine mortality because of the age structure. For example, both Iran and Germany have a similar population size of about 83 million in 2019 but annual mortality that year was almost 2.5 times higher in Germany^13^, mainly due to the German population being much older. For example, in Germany, 16% of the population are over the age of 65 whereas in Iran it is less than 4% (ref. ^14^). Hence, the expected deaths are higher in Germany than in Iran. Continuing with this example, an excess deaths estimate of (say) 100,000 in both countries would rank them identically on a per capita basis, but the P-score in Iran would be higher than Germany’s owing to it being a higher relative increase compared to the expected number of deaths.

### Global, regional and income group summary

Globally, for the period January 2020 to December 2021, we estimate 14.83 million excess deaths with an uncertainty interval (UI) of 13.23 million to 16.58 million, which is 2.74 (UI 2.44 to 3.06) times higher than the 5.42 million COVID-19 deaths reported to the WHO for this period. Throughout the paper, the reported UIs are 95% Bayesian credible intervals. We estimate 4.47 (UI 3.91 to 5.07) excess deaths in 2020 and 10.36 (UI 9.06 to 11.97) in 2021 globally. Turning to the P-scores, there were 7.97% (UI 6.96% to 9.03%) and 18.30% (UI 15.99% to 21.15%) increases in deaths globally in 2020 and 2021, respectively, compared to what we would have expected if the pandemic had not occurred.

**Fig. 1:**
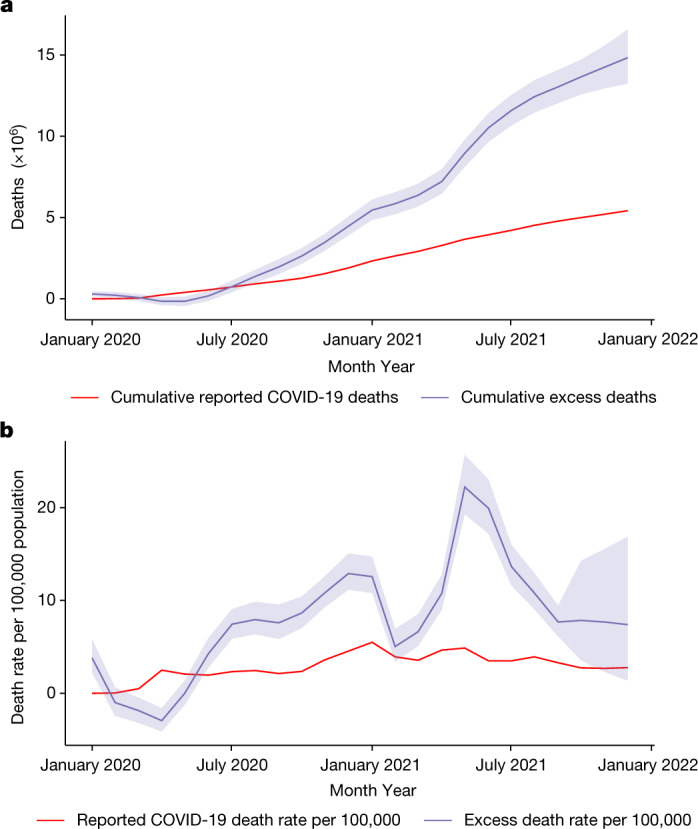
Global excess and reported COVID-19 deaths and death rates per 100,000 population. **a**, Cumulative global excess death estimates and the cumulative reported COVID-19 deaths by month from January 2020 to December 2021. **b**, Global excess death rates per 100,000 population and the reported COVID-19 death rates per 100,000 population, also by month, from January 2020 to December 2021. On both plots, the central lines of the excess mortality series show the mean estimates and the shaded regions indicate the 95% uncertainty intervals. Source data

The top panel of Fig. 1 shows the cumulative excess and reported COVID-19 deaths by month. We see the steepening of the curve in the middle of 2021. This sharp increase is almost entirely due to the estimate of the catastrophic wave that hit India at this time. In the bottom panel, the monthly excess death rate per 100,000 is plotted, and again, the peak towards the middle of 2021 is evident.

In the Extended Data Table 1 we provide summaries aggregated across all 194 WHO member states to give global estimates for the period January 2020 to December 2021. We also aggregate the member state estimates according to the six WHO regions and for the same period. The greatest contribution to the total is estimated for the SEAR region. The ratio of excess mortality to reported COVID-19 mortality is greatest in the AFR and SEAR regions, although we must emphasize that these are the regions with the greatest data paucity. As measured by the P-score, the worst affected regions were AMR (22%) and SEAR (22%), with EUR (17%) and EMR (12%) having intermediate values and AFR (8%) and WPR (0%) having the lowest values.

In the Extended Data Table 2 we also provide the corresponding summaries when the member state estimates are aggregated according to the four World Bank income groupings. There are wide variations across the respective economies but in general the estimates point to excess mortality being magnitudes higher than the reported COVID-19 mortality. Over 50% of the estimated excess occurs in lower-middle income economies. The low-income economies include much of sub-Saharan Africa, which reported relatively few COVID-19 deaths.

**Fig. 2:**
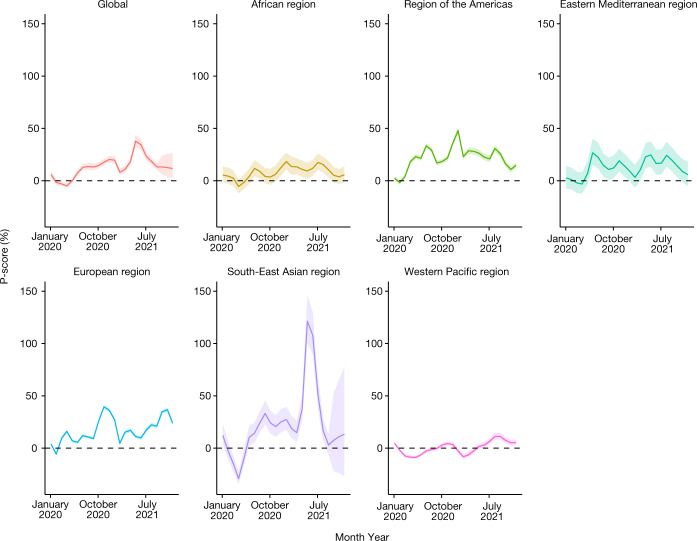
Global and WHO region P-scores (excess deaths relative to expected deaths). Monthly estimates of P-scores, expressed as a percentage, aggregated globally and for the six WHO regions for the period January 2020 to December 2021. All plots show the mean estimates and the 95% uncertainty intervals. Source data

In Fig. 2 we plot P-scores by month globally and for each WHO region. The global P-score increases steadily to the end of 2020, and then drops before a sharp increase in the middle of 2021, followed by a steady decline. The striking peak is in SEAR for the middle of 2021 for which we estimate that more than twice as many deaths have occurred, relative to those expected for this period. AMR has a peak in January 2021, and EUR has a peak at the end of 2020, and then drops before steadily increasing in 2021.

### Selected country summary

**Fig. 3:**
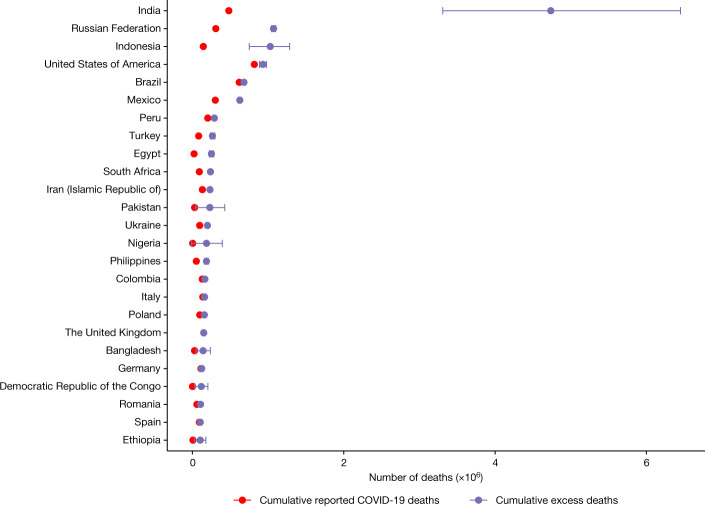
The 25 countries with the highest total estimated excess deaths between January 2020 and December 2021. The red dots show the total reported COVID-19 death numbers. The purple dots show the mean total estimated excess death numbers with the width of the bars showing the 95% uncertainty intervals. Source data

Figure 3 displays the excess deaths estimates and reported COVID-19 death counts for the 25 countries with the highest numbers of estimated excess deaths, along with error bars for the uncertainty interval. The 20 countries with the highest excess estimates represent approximately half (48.9%) the global population and account for over 80% of the estimated global excess deaths for the January 2020 to December 2021 period. These countries are (in alphabetical order) Bangladesh, Brazil, Colombia, Egypt, India, Indonesia, Iran, Italy, Mexico, Nigeria, Pakistan, Peru, the Philippines, Poland, the Russian Federation, South Africa, the United Kingdom, Turkey, Ukraine and the United States of America.

There are an estimated 4.74 million (UI 3.31 to 6.45 million) excess deaths for India alone in the period January 2020 to December 2021, followed by 1.07 million (UI 1.05 to 1.10 million) excess deaths in the Russian Federation, 1.03 million (UI 0.75 to 1.29 million) excess deaths in Indonesia and 932K (UI 887K to 978K) excess deaths in the United States of America. Figure 3 contains countries of all possible data types in our model:

- Countries with full ACM data for the entire analysed period (Russian Federation, United States of America, Brazil, Egypt, Spain and so on) for which the uncertainty in the estimates comes from the expected (counterfactual) number of deaths and is therefore relatively narrow.
- Countries with mixed ACM data (India, Indonesia, Turkey), that is, subnational data projected to the national level. This produces an additional layer of uncertainty from the projection of the information to the national level.
- Countries for which ACM data were unavailable (Pakistan, Nigeria, Ethiopia and so on), where the estimates are derived from the covariate prediction model. In these countries the uncertainty is the highest and results are to be interpreted with the greatest caution. However, the estimated undercount in these countries is well within the plausible range of undercounts as estimated for countries with full ACM data.

**Fig. 4:**
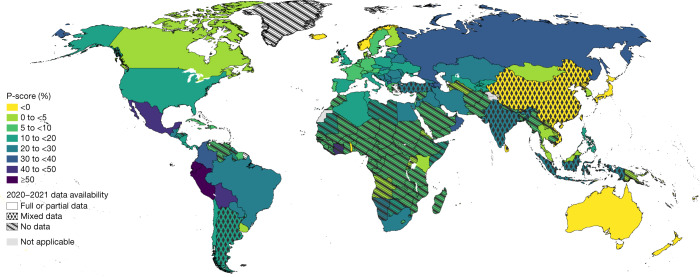
Mapping estimated P-scores (excess deaths relative to expected deaths). The map shows the geographic distribution of the mean P-scores for years 2020 and 2021 across all 194 WHO member states. The darker the colour the higher the estimated mean P-score. The patterns indicate the quality of the all-cause mortality data that were available for each respective country with the solid pattern showing full or partial data, dots for mixed data and diagonal lines for no data. Source data

**Fig. 5:**
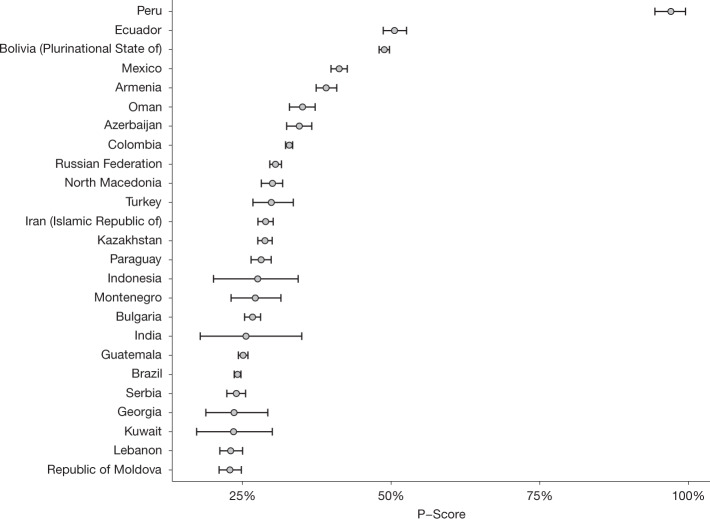
The 25 countries with the highest mean P-scores (excess deaths relative to expected deaths). The plot shows the 25 countries with the highest mean P-scores for years 2020 and 2021 after ranking all WHO member states with populations greater than 200,000 by mean P-score from highest to lowest. The mean for each country is shown using the central grey dots and the widths of the bars show the 95% uncertainty intervals. Source data

The total estimated excess deaths are heavily determined by the population size of each country. Figure 4 shows a map of the estimated P-scores for 2020–2021, and Fig. 5 displays the 25 countries with the highest estimated P-scores. In both figures the P-scores are calculated using expected numbers over 2020–2021.

From the P-score point of view, the worst impacted countries generally have smaller populations than those from Fig. 3. For example, India and the United States of America have the highest and fourth highest estimated excess deaths in the world in absolute terms but the United States of America is absent from the top 25 and India is 21st in the list. On the other hand, smaller countries such as Peru, Bulgaria and Bolivia have been impacted more heavily, relative to population size, when examining excess mortality in absolute terms.

So far as individual countries are concerned, Peru has a devastating P-score estimate of 97%, a doubling of deaths over the pandemic relative to what was expected. Other countries with a large increase include Ecuador with a 51% increase in deaths and Bolivia with a 49% increase.

**Fig. 6:**
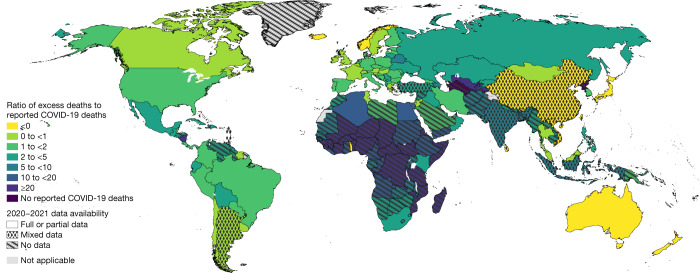
Mapping the ratio of total excess deaths to total reported COVID-19 deaths. The map shows the geographic distribution of the mean ratio of the total excess deaths to total reported COVID-19 deaths for years 2020 and 2021 across all 194 WHO member states. The darker the colour the higher the estimated mean ratio. The patterns indicate the quality of the all-cause mortality data that were available for each respective country with the solid pattern showing full or partial data, dots for mixed data and diagonal lines for no data. Source data

Figure 6 maps the ratio of excess deaths to reported COVID-19 deaths. There is a wide range for this excess measure, with many countries in the AFRO region having high ratios, and countries in Western Europe having ratios closer to 1 (with some, such as France, having values below 1). Globally, over January 2020–December 2021, there were 5,420,534 reported COVID-19 deaths, and according to our estimates, the ratio of excess to reported COVID-19 deaths is 2.74 (UI 2.44 to 3.06), which is a huge discrepancy.

As mentioned previously, we estimate the highest cumulative excess death numbers for India, accounting for 4.74 million deaths with a 95% credible interval of (3.31, 6.45) million. We base this estimate for India on subnational data—we have mortality data for 17 states and union territories (out of 36) over the pandemic. Using a proportionality assumption as discussed in the Methods and in Knutson et al.^15^, we produce a national estimate. In the Supplementary information we summarize excess estimates from other studies that use different data sources and illustrate that our estimates are consistent with these estimates.

A crucial component of the excess calculation is the estimation of the expected number of deaths. There are two elements to the calculation, the mortality data upon which it is based and the model that is adopted. First, with respect to the data, the WHO adjust the raw mortality counts, if there is perceived to be any incompleteness in reporting (and the scaling value may be carried forward to the pandemic period). We note that as part of the process to produce excess estimates, country consultation is carried out, in which the adjusted country numbers are shared with nationally nominated focal points who are tasked with reviewing the adjusted counts. Second, for the expected counts modelling, we used splines both for the annual trend and for the within-year seasonal variation. A country for which the completeness adjustment and spline modelling provided a less than satisfactory excess estimate was Germany. Under the default data process/spline modelling the excess estimate was 195,000 with 95% credible interval (161,000, 229,000). However, on closer examination this excess estimate was too high because of a combination of data/model issues. For Germany, ACM in 2016–2018 were scaled up owing to the completeness assessment, which lead to a dip in the ACM sequence in 2019. The annual spline fit to these adjusted data produced expected numbers that were too low (and therefore an excess that was too high). Hence, we reanalysed the Germany data with unadjusted data and a linear term, rather than a spline. This produced a more realistic excess estimate of 122,000 with a 95% credible interval of (101,000, 143,000).

For Sweden, we were concerned there were similar issues because of an unnecessary completeness adjustment of the raw mortality figure reported to the WHO in 2019 (the mortality count was lower than recent counts). The original excess estimate was 11,300 (9,900, 12,700). On closer scrutiny, we decided that this adjustment was not necessary and we redid the analysis for Sweden, again including a linear term for the annual trend instead of a spline (for the same reasons as described for Germany). This operation resulted in an estimate for Sweden that was higher, specifically 13,400 (11,700, 15,200). The changes in the excess estimates for Germany and Sweden do not change the global or EURO figures substantively (and the figures quoted in this paper are based on the revised estimates). As a side note, for both these countries when using the unadjusted data, both the linear and spline annual trend models produced similar excess estimates. However, using a spline for the annual trend can lead to sensitivity to the last year of pre-pandemic data, and a priority going forward is to systematically compare and evaluate different models for producing the expected numbers, building on recent work^16^. For the next round of estimates we will also revisit the under-reporting adjustment procedure. More details on revisiting the Germany and Sweden estimates are in the Supplementary information.

A natural inclination is to rank countries in terms of one of the metrics we have discussed. Basing rankings on point estimates will often be misleading because, particularly in the context of estimating excess mortality, there will often be considerable uncertainty in the chosen metric for a given country.

In the Methods, we include an illustrative example of a rankings analysis, using the excess rate. We show how one can evaluate the (posterior) probability that the excess is greater in one country than in another and provide graphical summaries to aid in interpretation. We also discuss the temporal comparison of rankings. In the Supplementary information, we describe a more substantive analysis that addresses how the United Kingdom compared to countries of the European Union, in terms of the excess rate and the P-score, over the pandemic.

In general, one can rarely simply look at the excess rate and associated rankings and make statements concerning the manner in which a country dealt with the pandemic, as there are many factors at play. These include: the age structure of the population, the population density and cultural practices, the government responses during different periods of the epidemic, how the population responded to government actions, and the infectiousness and fatality rates of the various variants that were present at different times.

## Discussion

These estimates call our attention to four important points. First and foremost, COVID-19 has resulted in marked global excess mortality: 14.83 million deaths (13.23, 16.58) over 2020–2021. In 2020, the excess was 4.47 (3.91, 5.07) million and in 2021, the excess was 10.36 (9.06, 11.97) million. The majority of the countries in the world have seen substantive increases in mortality.

Second, both the reported and excess estimated toll of COVID-19 were heavier in 2021 than in 2020. Third, excess mortality is much higher than reported COVID-19 mortality globally. In many countries COVID-19 deaths have been reported accurately, yet in others, the estimated excess mortality is much higher than reported COVID-19 mortality, occasionally by several orders of magnitude. In total, the estimates show that global excess mortality is 2.74 times higher than reported COVID-19 deaths. Finally, for almost half the countries of the world, tracking excess mortality is not possible using the data that are available and for these we must rely on statistical models. Critically, the missing countries are not randomly spread across the globe and so the countries with missing data may be systematically different from the sample of countries for which we do have data when we account for the age distributions of the populations, the underlying disease burdens of the countries, what is known about the strengths of their health systems and potentially most importantly, when, and how the pandemic evolved within their borders. These differences considered, some of the estimates and uncertainty intervals for locations for which we know very little must be interpreted with caution.

Despite its strengths, this study has important limitations extending beyond the data paucity mentioned above. The non-COVID counterfactual trend is derived using historical data and is sensitive to the assumptions made in the forecast. The weight given to recent data years relative to those further back in time, and how smoothly changes over time are projected to persist into the pandemic period, can influence the expected levels. We used spline models as the basis for the modelling of the expected numbers, but as we have mentioned will revisit this choice for the next round of estimates, as such models can produce inappropriate extrapolations. Another limitation relates to the quality of the input data used. The completeness of observed deaths and the consistency in quality as well as construct validity for the various covariates used in the regression model, impact the accuracy of the empirical estimates of excess and the robustness of the predictive model for deriving excess for countries in which deaths have not been observed^16^. Currently, completeness of reporting during the pandemic is based on historic completeness of registered deaths relative to the WHO Global Health Estimates (GHE)^17^. The GHE uses multiple data sources to generate country-, age-, sex-, year- and cause-specific mortality estimates, which in turn undergo extensive country consultation. However, comparing 2019 estimates of the GHE to registered deaths from even high-quality registration systems, some differences are observed. Subsequent iterations of the excess mortality work will include updates to how the expected deaths are derived and how completeness of reporting is calculated and extrapolated to the pandemic period.

The greater proportion of global excess is derived using observed mortality data and the estimated toll is staggeringly high. To place these estimates in context, the leading cause of death in 2019 was ischaemic heart disease, with 8.9 million deaths (https://www.who.int/news-room/fact-sheets/detail/the-top-10-causes-of-death). Information on the leading causes of death is not currently available for the pandemic years, but we would expect COVID-19 to be among the leading causes of death in 2020 and the leading cause of death in 2021. We estimate that the mean global per capita excess mortality rate was 0.06% in 2020, more than doubling to 0.13% in 2021. This surpasses the influenza pandemics of 1957, 1968 and 2009 (estimated at 0.04%, 0.03% and 0.005%, respectively)^18^. However, the 1918 influenza pandemic was magnitudes higher, with an estimated 1.0% per capita excess mortality rate, or 75 million global excess deaths when adjusted to the 2020 population^18^.

Excess mortality quantifies the increase in mortality from all causes, including direct COVID-19 deaths, indirect COVID-19 deaths (for example, health-system overload) and strictly non-COVID-19 deaths (for example, those resulting from other health shocks such as violent conflict or disasters). These estimates from the WHO cannot quantify the relative importance of each of these factors. However, considering the low levels of excess mortality in countries in which COVID-19 transmission, infection and mortality rates were low during some of the analysed period (for example, Malaysia, Mongolia, Uruguay in 2020) or its entirety (for example, Australia, Japan, New Zealand), suggests that in many countries the greater proportion of excess deaths can be attributed to COVID-19 directly. In fact, where accurately quantified, excess mortality may provide a reliable lower-bound on COVID-19 deaths considering that for several countries, we have mortality deficits or negative estimates for certain months. The greater number of these countries have high-quality reporting systems and this deficit is due to deaths from non-natural and natural causes decreasing during the analysed period^13^ and there having been less severe influenza seasons in 2020 and 2021 relative to previous years^19^. In many such countries, mortality improvements may be attributable to health systems being especially geared up to respond, the populations seeking early attention because of heightened sensitivity to health issues, compliance with public health and social measures that could reduce transmission of other infections and containment measures such as lockdowns. However, we are also aware that there have been non-COVID-19-related crises that have been experienced by some countries, for example, the conflict in Armenia, for which only a proportion of the excess mortality in 2020 would rightly be associated with COVID-19.

The alternative to excess mortality estimates—that is, relying on reported COVID-19 deaths—represents a severe undercount of the toll. Indeed, even in countries with ACM data for which the estimates are much more certain, mortality has risen substantially such that excess mortality is much higher than reported COVID-19 deaths, whether it is by 50% or by several hundred per cent. There is very little chance that the countries for which ACM data are not available have been able to report COVID-19 deaths accurately. There is ample, albeit preliminary evidence from many countries for which ACM data were not obtained (such as Pakistan and Haiti), that mortality has increased, and excess mortality is much higher than reported COVID-19 deaths. As another example, in the Africa region for which ACM data are most lacking, recent studies have highlighted the underestimation in the reported statistics^20^. In this region, the direct estimates of the ratio between excess mortality and reported COVID-19 deaths spans the gamut from about 1 in Tunisia to 2.62 in South Africa, 12 in Egypt and over 20 in Algeria. Thus, although a total regional estimate of a ratio of 8.03 might seem high at first glance, it is well within bounds of the more certain ratios and the true, yet unknown ratio, may very well be even higher both regionally and globally. As already noted, the excess estimates for sub-Saharan Africa are the least robust because of a paucity of data.

In the two years within which the COVID-19 pandemic has severely impacted humanity, important lessons remain to be fully documented and harnessed as part of the global public health surveillance capacity. First, the urgent need to improve data and health information systems and the way data are collected, analysed, shared and reported. Second, the required alignments of communicable disease surveillance with the continuous strengthening of health information systems and their integration with other existing routine surveillance systems, and with demographic and geographic monitoring systems to facilitate timely and targeted interventions. COVID-19 surveillance must also be combined with Universal Health Coverage and the International Health Regulations monitoring and related indicators for health-system preparedness, including vaccine coverage and water, sanitation and hygiene services.

As shown in the Supplementary information, there is a more than doubling of excess deaths when comparing 2021 to 2020. Despite the advances in diagnostics and therapeutics in 2020 and the rapid development of vaccines throughout the year, the end of 2020 saw the permeation of the virus into highly populous societies that had previously suffered limited exposure. In 2021, the rise in infections outpaced the roll-out of vaccines in many such locations and this either led to or was worsened by the emergence of more infectious, higher fatality, SARS-CoV-2 strains such as the Delta variant. We can speculate about how vaccine hesitancy, premature relaxation of containment measures and a global COVID-19 ‘fatigue’ contributed to how the pandemic developed but this is an important area for further study. And although the variants of SARS-CoV-2 continue to emerge (https://www.who.int/activities/tracking-SARS-CoV-2-variants) and are sequenced to identify variants that are likely to produce more serious illness, there remains limited information in real time to track them to create early warning systems of global import. This is critical as even the impact of less severe variants such as Omicron cannot be discounted among unvaccinated and older adult populations^21^.

To emerge from this crisis, the world needs to be able to monitor mortality and morbidity with real-time, reliable and actionable data. Strengthened country capacity for data and information requires collaboration across governmental and non-governmental institutions, including ministries of health and finance, hospitals, insurance companies, charities, national statistics institutions, offices of the registrar general, local and regional government, think tanks, academia and more. Monitoring systems of specific causes and ACM at the national, regional and global levels may serve as an early warning system for future health emergencies that will allow more timely responses to prevent local outbreaks from inflicting harms on lives and livelihoods in their immediate surroundings and across the world. Gaps in knowledge and data lead to gaps in response. It is thus vital for future responses that countries have well-functioning CRVS systems, which are the foundation upon which monitoring, prevention and future advancements on health rest.

WHO has made all of the results of the excess mortality estimates publicly available in an interactive web application at https://worldhealthorg.shinyapps.io/covid19excess/. This tool allows transparent exploration of estimates from the country level up to the regional and global levels. All data and code are available at https://github.com/WHOexcessc19/Codebase, so that our analyses and the results are completely reproducible.

The WHO excess mortality model is a live model that will be periodically updated given additional mortality data as well as data on covariates of relevance. We will also continue to improve the statistical framework and model. These estimates also serve as inputs to other important projects such as WHO’s GHE^17^ and the UN’s World Population Prospects^14^. Although WHO has made preliminary results available disaggregated by broad age groups and by sex for every country, region and the world and documented the method to do this^22^, this is work in progress and hence will be reported in a future paper.

## Methods

### Process

The process for producing the estimates of excess mortality consisted of three main steps. First, a Technical Advisory Group (TAG) was established to develop a set of methods that were used to produce estimates of excess deaths associated with the COVID-19 pandemic in countries. Second, WHO member states were consulted on the estimates, input data sources and methods. Finally, feedback from the countries was then incorporated into the modelling to update the estimates. The details of each step are described below.

In February 2021, the WHO, in collaboration with the United Nations Department of Economic and Social Affairs, formed the TAG on COVID-19 Mortality Assessment to advise on the development of analytical methods for estimating excess mortality in all countries. The TAG is composed of leading demographers, epidemiologists, economists, data and social scientists and statisticians from a range of backgrounds and geographies. A complete list of the TAG members is provided at the end of the paper. In addition to determining the levels and the age and sex distributions of the excess deaths associated with the COVID-19 pandemic, the expertise of the TAG has been leveraged to study the impact of the pandemic on broader areas such as inequality in COVID-19 mortality between and within countries, death registration and reporting systems, and how existing surveys and censuses can be used to fill in data gaps to quantify the impact of the pandemic. At the time of writing, this work is still ongoing.

In August 2021, a circular letter was sent to all WHO member states to nominate focal points to take part in country consultation. Member states were requested to review and provide feedback on the preliminary estimates of COVID-19 excess mortality and submit additional data that may not have been previously available to WHO. The first round of the country consultation was conducted between October and November 2021 through WHO’s Country Portal, an online platform to facilitate data exchange between member states and WHO, for which the draft estimates and methodology for each country were made available to the designated national focal points. Countries that had not nominated a focal point were approached through their respective WHO country office or permanent mission in Geneva, Switzerland.

Between October 2021 and February 2022, a global technical consultation and two information sessions with member states were held to brief them on the progress and exchange views on the methodology. A series of regional webinars and technical consultations with individual countries were also organized for further discussion on input data, methods and estimates. By the end of March 2022, 140 countries (or 72% of the 194 member states) had participated in the country consultation, 65 had provided some data and 76 had provided feedback, which was then used to generate updated estimates. The revised estimates for a 24-month period from January 2020 to December 2021 were shared with the national focal points in March 2022.

The process of generating the estimates of excess mortality associated with the COVID-19 pandemic has followed the Guidelines for Accurate and Transparent Health Estimates Reporting^23^. In view of the fast-changing situation surrounding the pandemic, the excess mortality estimates will continue to be refined and revised as more data are identified and the methodology evolves over time.

### Data

**Extended Data Fig. 1:**
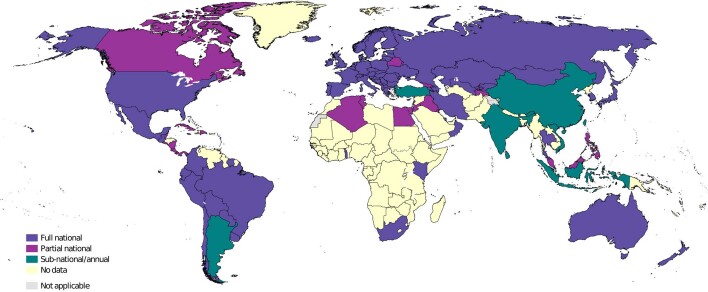
Mapping the availability of all-cause mortality data. The countries in dark blue have all 24 months of data available for January 2020 to December 2021 whereas those in purple have monthly data available but for less than 24 months. For the countries in green we only have either subnational or annual data for some or all of the period and for those in yellow do not have any representative all-cause mortality data available for the pandemic period.

The specific countries from each region for which data have been gathered are listed in the Supplementary information and are shown in Extended Data Fig. 1. Estimates of the excess mortality associated with the COVID-19 pandemic require historical ACM data that can be used to generate the death numbers under a hypothetical non-COVID-19 scenario, as well as ACM data for the target years against which the counterfactual is contrasted to calculate the excess. In the absence of nationally representative data, subnational data can be used to estimate national totals.

Reported ACM data at the national level on a weekly or monthly basis are available for only a subset of countries. The data used in this study span multiple sources:

- Data routinely shared with WHO as part of its standing agreement with member states as well as specifically provided to WHO in response to a data call for this project.
- Data that have been reported by European countries to Eurostat according to the European Statistical System^24^.
- Data that have been compiled for the Human Mortality Database as part of the Short-term Mortality Fluctuations project^25,26^.
- Data that have been compiled in the World Mortality dataset^13^.

Additionally, annual level data for 2020 and/or 2021 were obtained from the national statistics offices of China^27,28^, Grenada^29^, Saint Kitts and Nevis^30^, Saint Vincent and the Grenadines^31^, Sri Lanka^32^ and Vietnam^33^.

The countries with current reported ACM generally have ACM data for the pre-pandemic period as well. For those without such historical data, the WHO GHE^34^ database was used. Using the annual historic mortality we forecasted expected ACM to 2020 and 2021, to provide the expected mortality in these locations. The method for this forecast will be described shortly.

In addition to the data on reported ACM and the estimates from the GHE, the final dimension to the input data are variables that can potentially be used as predictors for excess mortality in those countries/time periods without ACM data. The strategy applied to create a covariate list was pragmatic and focused on identifying those variables that have been found to be contextually important and that have been measured/estimated in the majority of countries. The predictor variables are composed of both time-varying and time-invariant variables. Time-varying variables were the test positivity rate, temperature, confirmed COVID-19 death rate per 100,000 population (which is reported to the WHO), COVID-19 positive test rate per 100,000 population (from Our World in Data https://ourworldindata.org) and a variable constructed from a number of containment measures^35^. The COVID-19 death rate and the positive test rate are available for all member states for the entire period (https://covid19.who.int/). The time-invariant variables were a binary measure of the income level (low/middle versus high) and the historic diabetes prevalence and cardiovascular mortality rates as estimated by the Global Burden of Disease project^9^.

Subnational-level (states, provinces, cities, collections thereof and so on) data were obtained from various sources for Argentina^36^, India^37–42^, Indonesia^43^ and Turkey^44^.

### Statistical models

We write the excess in country $c$ at time $t$ as

1 $${\delta }_{c,t}={Y}_{c,t}-{E}_{c,t},$$

where ${Y}_{c,t}$ is the realized ACM and ${E}_{c,t}$ is the ACM that would be expected in the absence of the pandemic. Even for countries with fully observed ACM during the pandemic the excess is a random quantity, because we do not know the counts ${E}_{c,t}$ that would have occurred in the absence of the pandemic—the latter is the result of a modelling exercise, which produces forecasted ACM, with associated uncertainty.

The major challenges for modelling are to form a coherent approach in the face of disparate data sources of varying degrees of quality and in different spatially and temporally aggregated forms. We constructed a model from first principles within a Bayesian inferential framework, and as a first step developed a framework in which we directly model the raw death counts (as opposed to derived quantities such as rates). Death is binary, and so must follow a Bernoulli distribution, and it is also statistically rare, and so the Bernoulli can be accurately approximated by a Poisson distribution. The advantage of the latter is that it is amenable to manipulation when one considers subsets of availability such as over space (when subnational data only are available) or over time (when annual counts only are available). In the Poisson model the variance equals the mean, which is restrictive as mortality data typically exhibit greater variability than the nominal variance. Hence, we use models that allow for such excess-Poisson variation.

**Extended Data Fig. 2:**
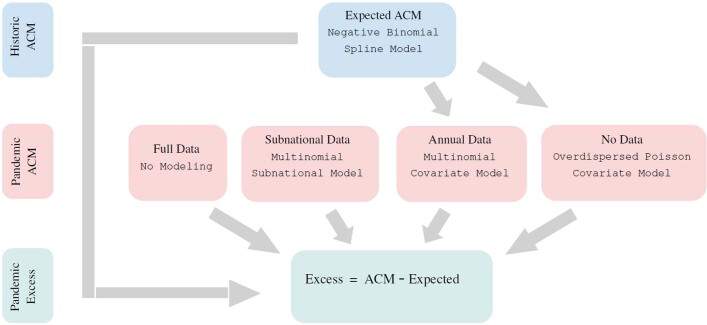
Overview of modelling strategy to produce excess mortality estimates for all countries. The flowchart outlines how the excess mortality associated with the COVID-19 pandemic is estimated. By definition, this is the difference between the all-cause mortality (ACM) during the pandemic period and that which was expected had the pandemic not occurred (light-green). Starting with the historic monthly ACM data (light-blue) we apply the Negative Binomial Spline model to generate the expected deaths for each country. The data that are available inform the modelling strategy employed to calculate the pandemic period ACM (light-pink). For the places with full data, the reported ACM are taken as is. For those countries with only subnational ACM data, a Multinomial Subnational model is employed to derive national level ACM based on the historic fractions of deaths observed in subnational regions. The overdispersed Poisson Covariate model is fitted to countries with monthly pandemic data, and this model is used to estimate pandemic period ACM for countries without any reported ACM. The covariate model is also used to infer the within-year monthly pandemic ACM in countries with only annual data, via the Multinomial Covariate model.

For modelling all countries of the world we need to consider various data situations. Although some countries have full data, others have annual or subnational data only, and for countries with no data we need to build a predictive model based on country-specific variables. Extended Data Figure 2 shows the relationship between the different models, and how they feed into the excess calculation.

### Model for expected numbers

For all countries and time points we model the expected numbers on the basis of historic data (for most countries, the period 2015–2019 was used for this modelling). We use a negative binomial model that allows for excess-Poisson variation. The annual historic yearly trend in ACM is modelled using a spline model, and within-year variation using a seasonal spline model. A spline is a flexible approach to modelling that allows departures from a linear association^45^. A negative binomial model has two parameters, a mean (which is obtained from spline components), and a scale parameter that accounts for excess-Poisson variation. The mean count for country $c$ and in month $t$ is modelled as:

$${{\rm{M}}{\rm{e}}{\rm{a}}{\rm{n}}{\rm{c}}{\rm{o}}{\rm{u}}{\rm{n}}{\rm{t}}}_{c,t}=\exp ({{\rm{a}}{\rm{n}}{\rm{n}}{\rm{u}}{\rm{a}}{\rm{l}}{\rm{t}}{\rm{r}}{\rm{e}}{\rm{n}}{\rm{d}}}_{c}+{{\rm{s}}{\rm{e}}{\rm{a}}{\rm{s}}{\rm{o}}{\rm{n}}{\rm{a}}{\rm{l}}{\rm{c}}{\rm{o}}{\rm{m}}{\rm{p}}{\rm{o}}{\rm{n}}{\rm{e}}{\rm{n}}{\rm{t}}}_{c,t})$$

where the annual trend uses a thin-plate spline and the seasonal component uses a cyclic cubic spline. After fitting to pre-pandemic data, we project the modelled trend forward to predict expected counts, by month, for 2020 and 2021. There is uncertainty in these predictions, which we incorporate into the excess mortality uncertainty intervals we produce.

For some countries, we only have national historic ACM data. For such countries we model within-year variation using temperature as a surrogate for seasonality. Full details of all modelling steps are given in Knutson et al.^15^.

### Model for countries without full pandemic data

For almost half of the 194 WHO member states we do not have the ACM counts over the pandemic, and so must predict them using country characteristics. We choose a simple form for this prediction model, with mean

2 $$\text{E}\left[{Y}_{{ct}}{\rm{|}}{E}_{c,t}\right]={E}_{c,t}{\theta }_{c,t},$$

where ${\theta }_{c,t} > 0$ is a relative rate parameter. If ${\theta }_{c,t} > 1$, then for country $c$ and at month $t$ the mortality is greater than expected, whereas if ${\theta }_{c,t} < 1$, then for country $c$ and at month $t$ the mortality is less than expected. We used $G$ time-invariant variables, ${Z}_{{gc}}$ (these are annual values from 2019). These were an indicator of high income and/or low or middle income, the cardiovascular mortality rate in 2019 and the diabetes prevalence rate in 2019. In addition, we used $B$ time-varying variables: a containment variable (it is calculated using all ordinal containment and closure policy indicators and health-system policy indicators, for further details see Hale et al.^14^), the square root of the reported COVID-19 death rate, temperature and the COVID-19 test positivity rate. We then build a log-linear model for the rate parameter:

3 $$\log {\theta }_{c,t}=\mathop{\underbrace{\alpha }}\limits_{{\rm{I}}{\rm{n}}{\rm{t}}{\rm{e}}{\rm{r}}{\rm{c}}{\rm{e}}{\rm{p}}{\rm{t}}}+\mathop{\underbrace{\mathop{\sum }\limits_{g=1}^{G}{\gamma }_{g}\,{Z}_{gc}}}\limits_{{\rm{T}}{\rm{i}}{\rm{m}}{\rm{e}}-{\rm{i}}{\rm{n}}{\rm{v}}{\rm{a}}{\rm{r}}{\rm{i}}{\rm{a}}{\rm{n}}{\rm{t}}\,{\rm{c}}{\rm{o}}{\rm{n}}{\rm{t}}{\rm{r}}{\rm{i}}{\rm{b}}{\rm{u}}{\rm{t}}{\rm{i}}{\rm{o}}{\rm{n}}{\rm{s}}}+\mathop{\underbrace{\mathop{\sum }\limits_{b=1}^{B}{\beta }_{bt}{X}_{bct}}}\limits_{{\rm{T}}{\rm{i}}{\rm{m}}{\rm{e}}-{\rm{v}}{\rm{a}}{\rm{r}}{\rm{y}}{\rm{i}}{\rm{n}}{\rm{g}}\,{\rm{c}}{\rm{o}}{\rm{n}}{\rm{t}}{\rm{r}}{\rm{i}}{\rm{b}}{\rm{u}}{\rm{t}}{\rm{i}}{\rm{o}}{\rm{n}}{\rm{s}}}+\mathop{\underbrace{{{\epsilon }}_{c,t}\,}}\limits_{{\rm{E}}{\rm{x}}{\rm{c}}{\rm{e}}{\rm{s}}{\rm{s}}-{\rm{P}}{\rm{o}}{\rm{i}}{\rm{s}}{\rm{s}}{\rm{o}}{\rm{n}}\,{\rm{v}}{\rm{a}}{\rm{r}}{\rm{i}}{\rm{a}}{\rm{t}}{\rm{i}}{\rm{o}}{\rm{n}}}$$

where ${\rm{\exp }}\left({\gamma }_{g}\right)$ and ${\rm{\exp }}\left({\beta }_{{bt}}\right)$ denote relative rate parameters and ${{\epsilon }}_{c,t}\sim {\rm{N}}(0,{{\sigma }}_{{\epsilon }}^{2})$ are independent error contributions that pick up random variation unexplained by the log-linear regression function. The time-varying coefficients allow the associations to evolve during the pandemic. As we desire the evolution to be smooth in time, for these time-varying coefficients ${\beta }_{c,t}$ we use a random walk of order 2 (RW2) prior that encourages smooth estimates^46^. In equation (2) above, we have conditioned on known expected numbers. In reality, and as just described, these are modelled to give a distribution over plausible values. The uncertainty in the expected predictions ${E}_{c,t}$ is well modelled by a gamma distribution, and the advantage of this choice is that it can be conveniently combined with a Poisson model to produce a negative binomial model with the log-linear mean given by equation (3). Full details (including evidence of the accuracy of the gamma model) can be found in Knutson et al.^15^.

This model was fitted to all countries with observed monthly ACM data over some portion of 2020–2021, using the integrated nested Laplace approximation method^47^, to obtain posterior distributions over the unknown parameters. The resultant posterior distribution reflects the uncertainty in the parameters (both in the expected numbers and the log-linear covariate model), and can be used to construct a predictive distribution for the ACM in countries with no data or partial data.

For some countries, only subnational data were available, and so we construct a model for the national ACM data using a proportionality assumption, expanding on previous work^48^. We describe the model in the context of India. We use ACM data from 17 states and union territories out of 36 (data from different numbers of states are available in different pandemic months) to infer the national total, under the assumption that the proportion of deaths in the states with available data remains approximately constant over time. For example, if a state historically accounts for 10% of deaths in India, one would predict a national death total of 10$\times$ the observed number of deaths in that state only. Under the Poisson framework, this proportionality assumption yields a multinomial distribution for the fractions of deaths and we can predict the unknown national totals over the course of the pandemic after fitting the multinomial model.

Extensive model validation was carried out for both the countries with no data, and those with subnational data only. This included exercises in which we systematically removed all data for each country in turn, or we removed data for all countries for single months. We then predicted these removed data using the retained data and evaluated model performance using metrics such as bias and the coverage of prediction intervals. Results for these exercises can be found in the supplementary materials of Knutson et al.^15^. We emphasize that the model (3) is not used for countries with subnational data.

For other countries that have annual (but not monthly) national data during the pandemic, we lean on the fact that the distribution of Poisson monthly counts, given the annual count, is multinomial with probabilities that are the normalized rate parameters, that is,

$${p}_{c,t}=\frac{{E}_{c,t}{\theta }_{c,t}}{\mathop{\sum }\limits_{{t}^{{\prime} }=1}^{12}{E}_{c,{t}^{{\prime} }}{\theta }_{c,{t}^{{\prime} }}}.$$

where the rates ${\theta }_{c,t}$ are defined via the log-linear covariate model (3). This gives us a way to apportion the annual counts to the constituent months.

Our approach, differs from those of the other two global endeavours of the Institute for Health Metrics and Evaluation (IHME)^49^ and *The Economist*^50^. We have used a very conventional statistical modelling approach in which a parametric model is fitted using Bayesian inferential machinery, and with the models for different data types being consistent with each other to make the country by country results directly comparable to each other. As an example, if the mortality in subnational regions are Poisson random variables, then the sum (the mortality in the country) is also Poisson. Further, given the total mortality in a country the subnational counts follow a multinomial distribution. Our framework exploits these relationships when we formulate models for the situation in which we have subnational data only. Similarly, our annual model (for countries with such data only) is consistent with the monthly models we use for the majority of the countries. The IHME approach is unprincipled and not transparent and corresponds to a number of steps being bolted together, without a coherent model tying them together. Rather than using a direct count model based on a Poisson framework, the IHME approach models the log of the excess rate as a function of covariates, without any weighting, so that the population sizes of the different countries do not feed into the uncertainty calculation. A fundamental problem with the overall approach is that the uncertainty intervals are constructed in a non-standard and ad hoc way, so that the confidence intervals, in particular, will not be accurate representations of the true uncertainty. *The Economist* approach models the excess rate with a flexible tree-based machine learning technique, gradient boosting. The approach is clearly described and uses a resampling technique, the bootstrap, to form interval estimates, but there is no theory to support the use of the bootstrap with boosting, and so again, the uncertainty intervals should be viewed sceptically. A full description and critique of the alternative methods are available in Knutson et al.^15^. In the Supplementary information, we provide a comparison between point and interval country estimates obtained by the methods of the WHO, IHME and *The Economist*.

### P-scores

Recall that the P-score is defined as the ratio of the excess to the expected, expressed as a percentage. Mathematically, this corresponds to,

$${\text{PS}}_{c,t}=100\times \frac{{Y}_{c,t}-{E}_{c,t}}{{E}_{c,t}},$$

and ${\text{PS}}_{c,t}\ge -100,$ with zero deaths corresponding to –100, negative values corresponding to fewer deaths than expected and larger positive values corresponding to increasing levels of relative excess mortality. Under the model (2), we have $\text{E}\left[{Y}_{c,t}\right]={E}_{c,t}{\theta }_{c,t}$ so that

$$\text{E}\left[{\text{PS}}_{c,t}\right]=100\times \left({\theta }_{c,t}-1\right).$$

For countries whose ACM is unobserved, the rate is modelled via the log-linear form (equation (3)) which gives a specific form to the manner in which we assume the P-score changes as a function of country-specific covariates.

### Rankings

A natural, if sometimes unfortunate, inclination is to attempt to rank regions or countries in terms of the various metrics. Statistically, this is fraught with difficulties. The easiest approach, which is often followed in the media, is to simply rank on the basis of a point estimate of the metric, such as the mean or the median. The obvious problem with this approach is that the uncertainty in estimation is not accounted for. Using the Bayesian machinery that we use for inference we can account for the uncertainty probabilistically. In the simplest case of two countries, let ${X}_{1}$ and ${X}_{2}$ represent the excess rates in countries 1 and 2, respectively. We can then evaluate the (posterior) probability that ${X}_{1} > {X}_{2}$, and report this, rather than a binary statement that country 1 has a higher rate than country 2. The extension to multiple countries is immediate, as is the ability to calculate the probability of a higher rate in one country as compared to any collection of other countries.

**Extended Data Fig. 3:**
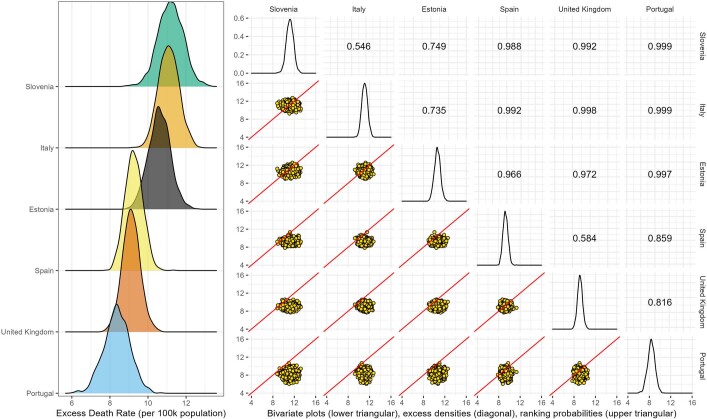
Excess mortality densities and ranking probabilities for select countries. Left: Ridgeplots representing the uncertainty in the cumulative excess monthly mortality rate over January 2020–December 2021 for six European countries. Right: Bivariate plots of pairs of excess rates (lower triangular), 1-dimensional summaries for individual countries (diagonal), and probabilities that the excess for the country labelled on the left exceeds the rate for the country labelled at the top (upper triangular). These probabilities are the fraction of points that lie below the red line in the corresponding bivariate plot.

For illustration of the issues of assessing rankings, we select six European countries that have overlap in their excess rate uncertainty (that is, posterior) distributions. In the left panel of Extended Data Fig. 3 we display posterior distributions for the excess rates of the six countries, ordered from top to bottom by highest to lowest median excess rate. There is clearly overlap in many of the distributions, but quantitative statements on the rankings require more than these plots. In the right panel of Extended Data Fig. 3 we present scatterplot representations of the bivariate probability distributions describing the relationships between pairs of countries. The red lines offer a reference by which we can evaluate the ranking probabilities (by calculating the fractions of points that are either side of the line). For example, the probabilities that the rate for Slovenia is greater than that of each of Italy, Estonia, Spain, the United Kingdom and Portugal are 0.546, 0.749, 0.988, 0.992 and 0.999, respectively. Even these plots do not give the complete picture as they are two-dimensional summaries of a six-dimensional object (the probability distribution over the six rates). We can provide other summaries, for example, the probability that the rate in Slovenia is greater than the rates in all of the other five countries is 0.479.

**Extended Data Fig. 4:**
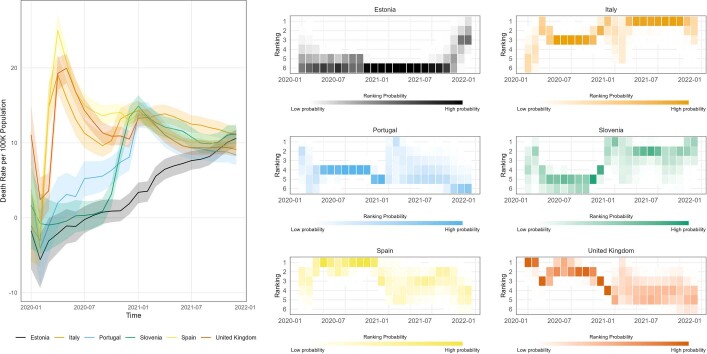
Excess rates over time and ranking probabilities for select countries. : Left: excess monthly mortality rate (per 100,000 of population), with 95% uncertainty intervals, by month, for six European countries. The panels to the right display the ranking probabilities for each of the individual countries. The probabilities sum to one for each country within each month, i.e., a country has to be in one of the six ranking positions. Also for a fixed month and ranking position across all countries the probabilities sum to one because one of the countries has to be in rank 1, 2, etc, in each month.

The rankings just discussed are based on the cumulative excess rate over January 2020–December 2021. Another potentially interesting summary is the relative rankings of countries’ rates over time. In Extended Data Fig. 4 we plot the excess rate over time (top panel) and the ranking probabilities (bottom panels). In each month, we calculate the probabilities that the rate of each country is highest, second highest and so on. In 2020, we see that among the six countries considered, Spain, the United Kingdom and to a lesser extent Italy, have high rates, whereas in 2021, Italy and Slovenia and to a lesser extent Spain have high rates. The rate in Estonia is generally low, apart from the last few months of 2021.

The Supplementary information contains a more substantive example where we consider the rankings of 27 countries of the European Union and the United Kingdom over time, in terms of both the excess rate and the P-score.

### Reporting summary

Further information on research design is available in the Nature Portfolio Reporting Summary linked to this article.

## Online content

Any methods, additional references, Nature Portfolio reporting summaries, source data, extended data, supplementary information, acknowledgements, peer review information; details of author contributions and competing interests; and statements of data and code availability are available at 10.1038/s41586-022-05522-2.

### Supplementary information

Supplementary Information Comparison of WHO estimates with those of *The Economist* and IHME, country rankings on excess mortality estimates, additional analyses for Germany and Sweden, estimates for India and tables of annual estimates by country (these sections include Supplementary Figs. 1–14 and Supplementary Tables 1–13).

Reporting summary

Peer Review File

### Source data

Source Data Fig. 1.

Source Data Fig. 2.

Source Data Fig. 3.

Source Data Fig. 4.

Source Data Fig. 5.

Source Data Fig. 6.

## Figures and Tables

**Extended Data Table 1:**
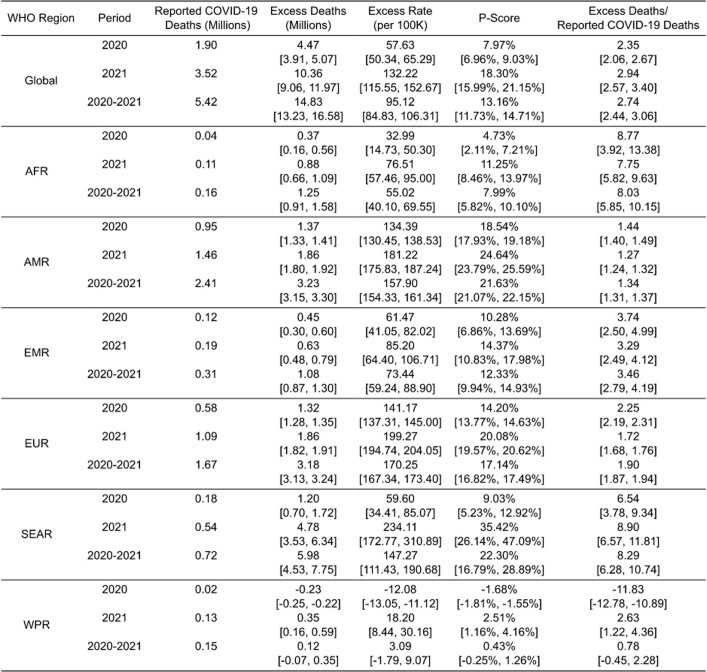
Cumulative reported COVID-19 and excess mortality measures by region

The measures shown are excess deaths, excess rates per 100,000 population, P-Scores and the ratio of excess to reported deaths. Estimates are aggregated by period for 2020, 2021 and both years combined and by region defined as Global=194 WHO member states and then six WHO regions for AFR=African Region, AMR=Region of the Americas, EMR=Eastern Mediterranean Region, EUR=European Region, SEAR=South East Asian Region, WPR=Western Pacific Region.

**Extended Data Table 2:**
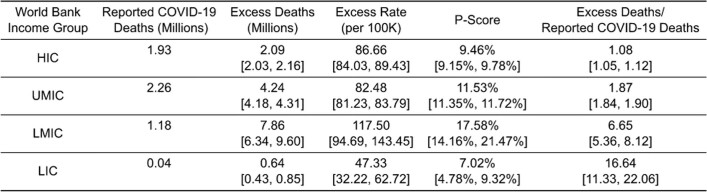
Cumulative reported COVID-19 and excess mortality measures by income group

The measures shown are excess deaths, excess rates per 100,000 population, P-Scores and the ratio of excess to reported deaths. Estimates are aggregated over 2020 and 2021 and by the 2021 World Bank income groupings defined as HIC=High-Income Economies, UMIC=Upper-Middle-Income Economies, LMIC=Lower-Middle Income Economies, LIC=Low-Income Economies.

## Data Availability

All data used as inputs to our modelling analyses are contained in model input files located at https://github.com/WHOexcessc19/Codebase. The site also contains all generated quantities and output datasets. Source data are provided with this paper.

## Code availability

All analyses were performed using R version 3.6.3. Scripts are available at https://github.com/WHOexcessc19/Codebase, which allows the estimates to be reproduced.
