# Supplementary material for: The WHO estimates of excess mortality associated with the COVID-19 pandemic
Source: Nature. 2022 Dec 14;613(7942):130–7. doi: 10.1038/s41586-022-05522-2 (PMC9812776; doi:10.1038/s41586-022-05522-2)
Supplement: Supplementary file 1 — Comparison of WHO estimates with those of The Economist and IHME, country rankings on excess mortality estimates, additional analyses for Germany and Sweden, estimates for India and tables of annual estimates by country (these sections include Supplementary Figs. 1–14 and Supplementary Tables 1–13). [file 41586_2022_5522_MOESM1_ESM.pdf]

nature portfolio

https://doi.org/10.1038/s41586-022-05522-2

Supplementary information

The WHO estimates of excess mortality associated with the COVID-19 pandemic

In the format provided by the authors and unedited

Supplementary Materials for "The WHO estimates of excess mortality associated with the COVID-19 pandemic"

William Msemburi[1], Ariel Karlinsky[2], Victoria Knutson[3], Serge Aleshin-Guendel[3], Somnath Chatterji[1] and Jon Wakefield[3,4]

[1]World Health Organization, Geneva, Switzerland
[2]Hebrew University, Jerusalem, Israel
[3]Department of Biostatistics, University of Washington, Seattle, USA
[4]Department of Statistics, University of Washington, Seattle, USA

Correspondence:
William Msemburi,
Division for Data, Analytics and Delivery for Impact,
World Health Organization,
Email: msemburiw@who.int

Contents

Comparison of WHO Estimates with those of The Economist and IHME 3
Country rankings on excess mortality estimates 10
Additional Analyses for Germany and Sweden 20
Estimates for India 22
Tables of annual estimates by country 25

Comparison of WHO Estimates with those of The Economist and IHME

In this section we provide numerical comparisons of the three sets of estimates from WHO, IHME and The Economist. In Tables 1 and 2 we provide the point and interval estimates for the cumulative excess deaths to the end of 2020 and 2021, respectively, comparing the aggregates for the WHO regions according to the three different sources. We see very large differences in EMR, AFR and WPR. For EMR, the WHO estimate is about half of that from the other two approaches whereas for AFR, the IHME estimate is just under double that of the WHO estimate and significantly larger than that from the Economist. The WPR region has wide variation with the Economist estimate being almost double the IHME estimate and several times higher than the WHO estimate.

In Figure 1 we plot the IHME point estimates for 2020–2021 versus the WHO estimates, highlighting the countries with larger differences. It is difficult to determine the excess mortality in China, and the WHO estimate is lower than both IHME and The Economist, since it is based on the official annual mortality.

Figure 2 plots the IHME width of 95% CIs versus the WHO width. It is hard to glean any clear patterns here. This is perhaps not surprising since, as described in Knutson et al. (2023), the IHME method for constructing CIs is completely unprincipled, and so the intervals should be viewed skeptically. In general, the intervals are narrower than those of WHO and The Economist. Later we will discuss the excess estimates for India, and in Table 4 we see the IHME interval estimate for India is very narrow, and does not seem credible.

In Figure 3 we plot the IHME point estimates for 2020–2021 versus The Economist estimates, highlighting the countries with larger differences. In general, the WHO estimates are closer to those of The Economist than to those of IHME. The estimates for China are again very different.

Figure 4 plots The Economist width of 95% CIs versus the WHO width. The Economist method does not account for uncertainty in the expected numbers, and so for those countries with observed mortality data in the pandemic, there is no uncertainty in the excess estimate – these are the points on the bottom axis (a large number of these points are in the EUR and AMR regions). Otherwise, the uncertainty from The Economist modeling is in general larger, but as we discuss in detail in Knutson et al. (2023). The Economist uses the bootstrap to estimate uncertainty, and at time of writing, no theory exists to justify its use in the context of gradient boosting.

Source	Region	period	Excess mean	Excess low	Excess high	Ratio mean	Ratio low	Ratio high
Economist	Global	2020	5 596 861	3 591 146	6 243 631	2.95	1.89	3.29
IHME	Global	2020	4 195 055	3 020 412	6 055 527	2.21	1.59	3.19
WHO	Global	2020	4 471 371	3 905 364	5 065 204	2.35	2.06	2.67
Economist	AFR	2020	358 103	-435 599	633 339	8.5	-10.34	15.04
IHME	AFR	2020	276 600	168 457	434 571	6.57	4	10.32
WHO	AFR	2020	369 562	164 952	563 481	8.77	3.92	13.38
Economist	AMR	2020	1 362 940	1 330 748	1 379 355	1.44	1.4	1.45
IHME	AMR	2020	1 257 563	1 054 514	1 784 418	1.33	1.11	1.88
WHO	AMR	2020	1 368 283	1 328 129	1 410 412	1.44	1.4	1.49
Economist	EMR	2020	878 396	488 366	1 000 168	7.36	4.09	8.38
IHME	EMR	2020	520 602	285 859	853 216	4.36	2.39	7.15
WHO	EMR	2020	446 076	297 920	595 261	3.74	2.5	4.99
Economist	EUR	2020	1 330 274	1 262 326	1 361 210	2.28	2.16	2.33
IHME	EUR	2020	974 776	770 885	1 312 382	1.67	1.32	2.24
WHO	EUR	2020	1 316 962	1 280 911	1 352 700	2.25	2.19	2.31
Economist	SEAR	2020	1 553 194	397 812	2 129 423	8.43	2.16	11.56
IHME	SEAR	2020	1 118 000	668 731	1 767 373	6.07	3.63	9.6
WHO	SEAR	2020	1 204 810	695 574	1 719 513	6.54	3.78	9.34
Economist	WPR	2020	117 785	-220 727	646 937	5.95	-11.14	32.66
IHME	WPR	2020	45 262	28 389	63 881	2.29	1.43	3.23
WHO	WPR	2020	-234 322	-253 210	-215 661	-11.83	-12.78	-10.89

Supplementary Table 1: Cumulative estimated excess deaths by WHO region and ratio of estimated excess deaths to reported COVID-19 deaths, January 2020 to December 2020, comparing etimates from the Economist, IHME and the WHO. Global=194 WHO member states, AFR=African Region, AMR=Region of the Americas, EMR=Eastern Mediterranean Region, EUR=European Region, SEAR=South-East Asian Region, WPR=Western Pacific Region

Source	Region	period	Excess mean	Excess low	Excess high	Ratio mean	Ratio low	Ratio high
Economist	Global	2020 and 2021	17 733 569	12 238 979	21 074 628	3.27	2.26	3.89
IHME	Global	2020 and 2021	18 200 000	17 100 000	19 600 000	3.36	3.15	3.62
WHO	Global	2020 and 2021	14 834 866	13 231 107	16 580 230	2.74	2.44	3.06
Economist	AFR	2020 and 2021	1 602 731	282 007	1 924 480	10.31	1.81	12.38
IHME	AFR	2020 and 2021	2 090 000	1 720 000	2 720 000	13.44	11.06	17.49
WHO	AFR	2020 and 2021	1 248 553	909 942	1 578 048	8.03	5.85	10.15
Economist	AMR	2020 and 2021	3 254 290	3 193 805	3 319 830	1.35	1.33	1.38
IHME	AMR	2020 and 2021	4 220 000	4 000 000	4 450 000	1.75	1.66	1.85
WHO	AMR	2020 and 2021	3 227 943	3 154 917	3 298 341	1.34	1.31	1.37
Economist	EMR	2020 and 2021	2 301 938	1 386 922	2 653 499	7.4	4.46	8.53
IHME	EMR	2020 and 2021	2 240 000	1 900 000	2 640 000	7.2	6.11	8.49
WHO	EMR	2020 and 2021	1 075 999	867 866	1 302 531	3.46	2.79	4.19
Economist	EUR	2020 and 2021	3 353 014	3 160 431	3 433 558	2.01	1.89	2.06
IHME	EUR	2020 and 2021	3 700 000	3 620 000	3 780 000	2.22	2.17	2.26
WHO	EUR	2020 and 2021	3 180 157	3 125 695	3 239 004	1.9	1.87	1.94
Economist	SEAR	2020 and 2021	6 364 975	2 654 580	9 957 292	8.83	3.68	13.81
IHME	SEAR	2020 and 2021	5 500 000	5 100 000	5 930 000	7.63	7.07	8.22
WHO	SEAR	2020 and 2021	5 982 010	4 526 314	7 745 465	8.29	6.28	10.74
Economist	WPR	2020 and 2021	849 412	234 442	2 118 935	5.5	1.52	13.72
IHME	WPR	2020 and 2021	462 000	416 000	523 000	2.99	2.69	3.39
WHO	WPR	2020 and 2021	120 205	-69 744	352 798	0.78	-0.45	2.28

Supplementary Table 2: Cumulative estimated excess deaths by WHO region and ratio of estimated excess deaths to reported COVID-19 deaths, January 2020 to December 2021, comparing etimates from the Economist, IHME and the WHO. Global=194 WHO member states, AFR=African Region, AMR=Region of the Americas, EMR=Eastern Mediterranean Region, EUR=European Region, SEAR=South-East Asian Region, WPR=Western Pacific Region.

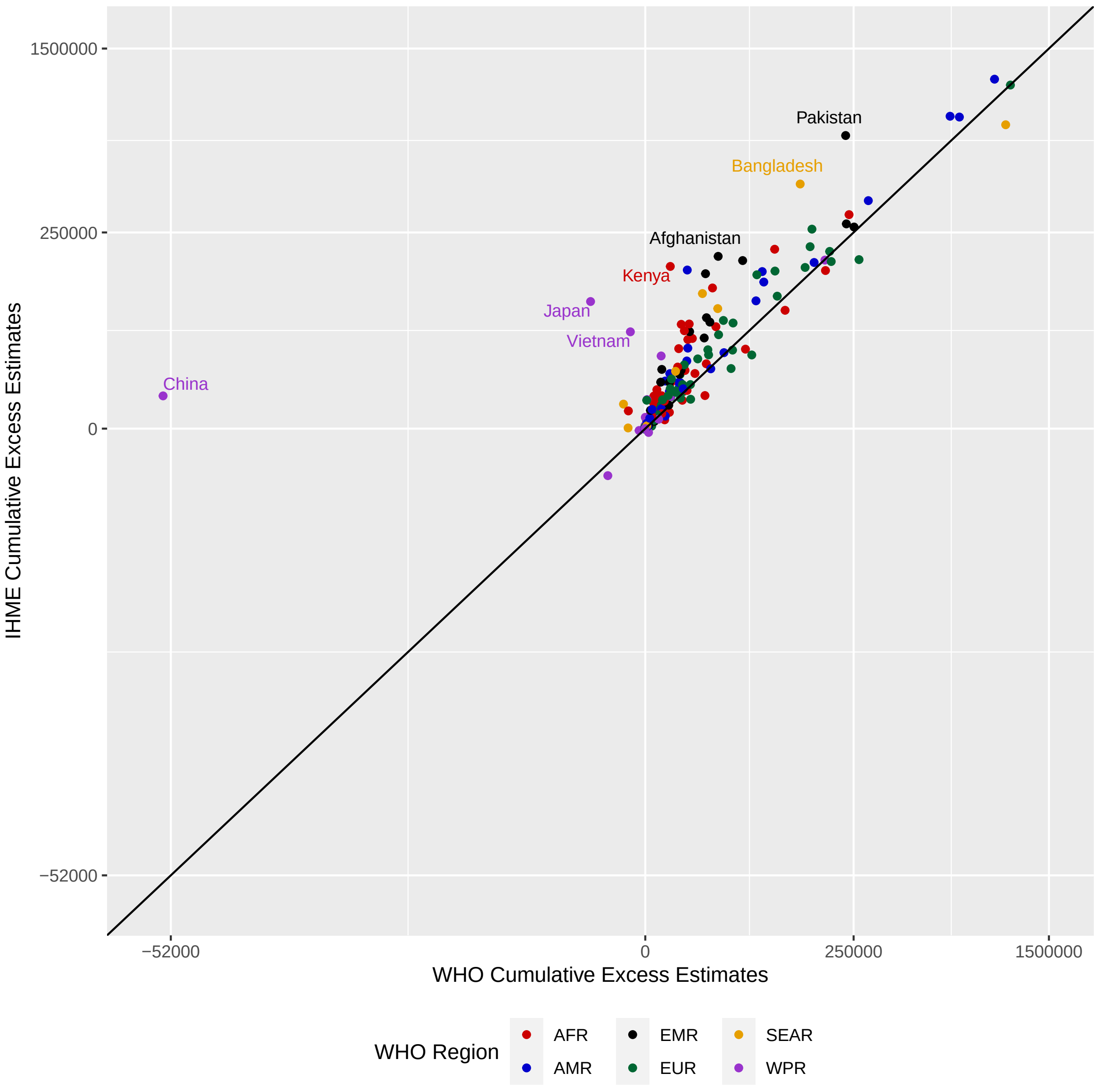

Supplementary Figure 1: Cumulative estimates of excess mortality for IHME plotted against WHO, January 2020 to December 2021. Color-coded by WHO region.

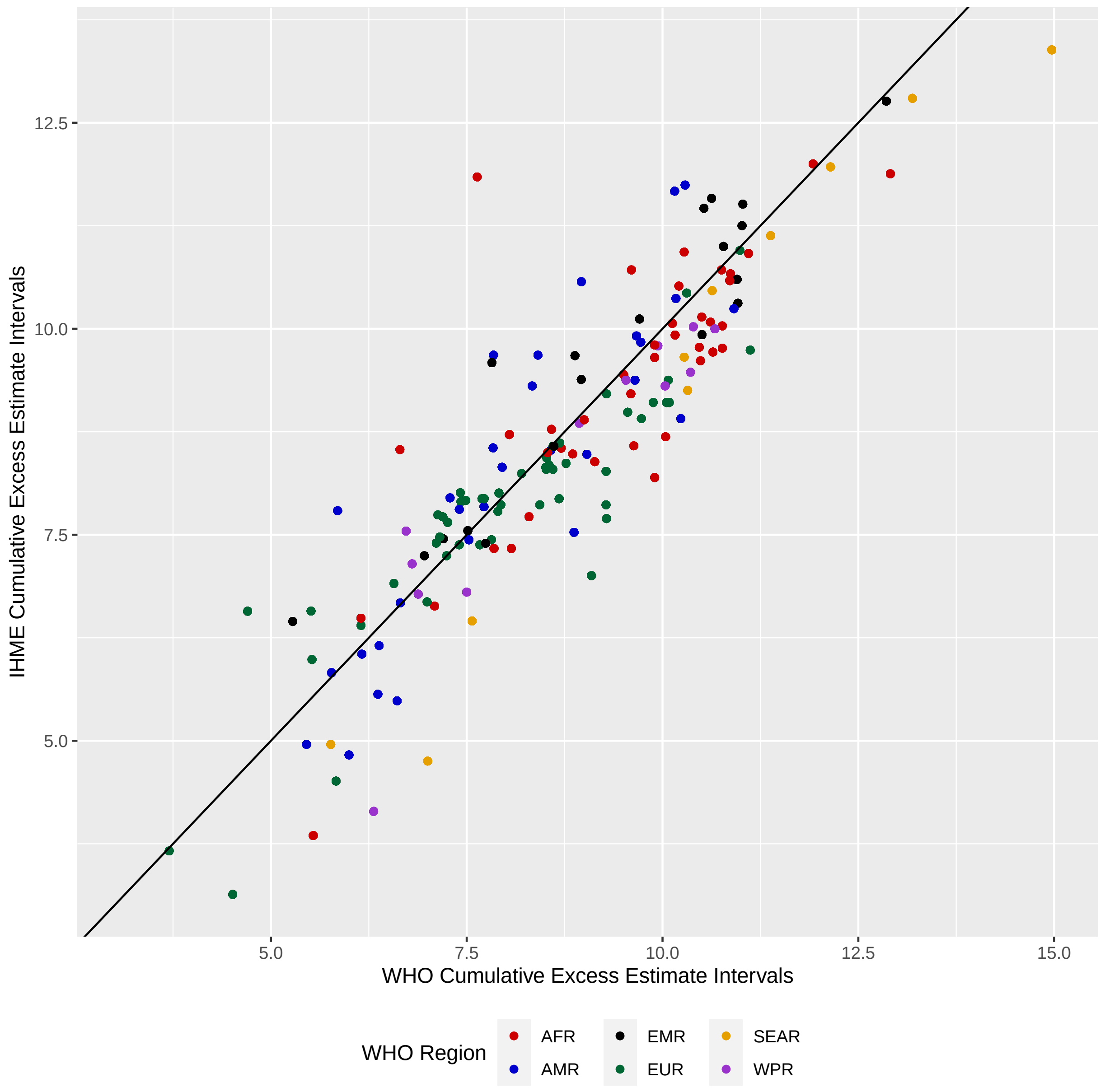

Supplementary Figure 2: 95% CI width of cumulative estimates of excess mortality for IHME plotted against WHO, January 2020 to December 2021. Color-coded by WHO region.

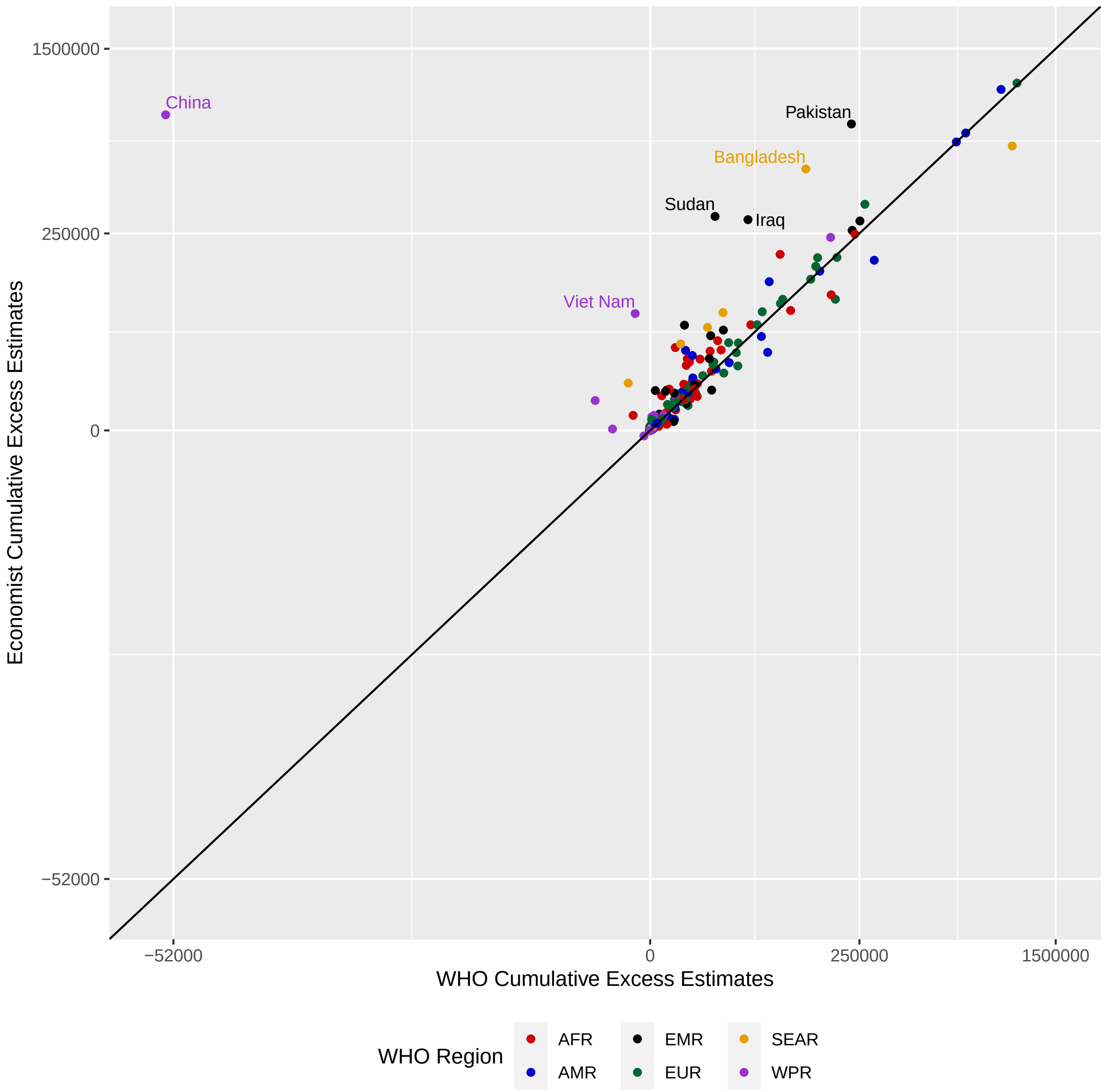

Supplementary Figure 3: Cumulative estimates of excess mortality, for The Economist plotted against WHO, January 2020 to December 2021. Color-coded by WHO region.

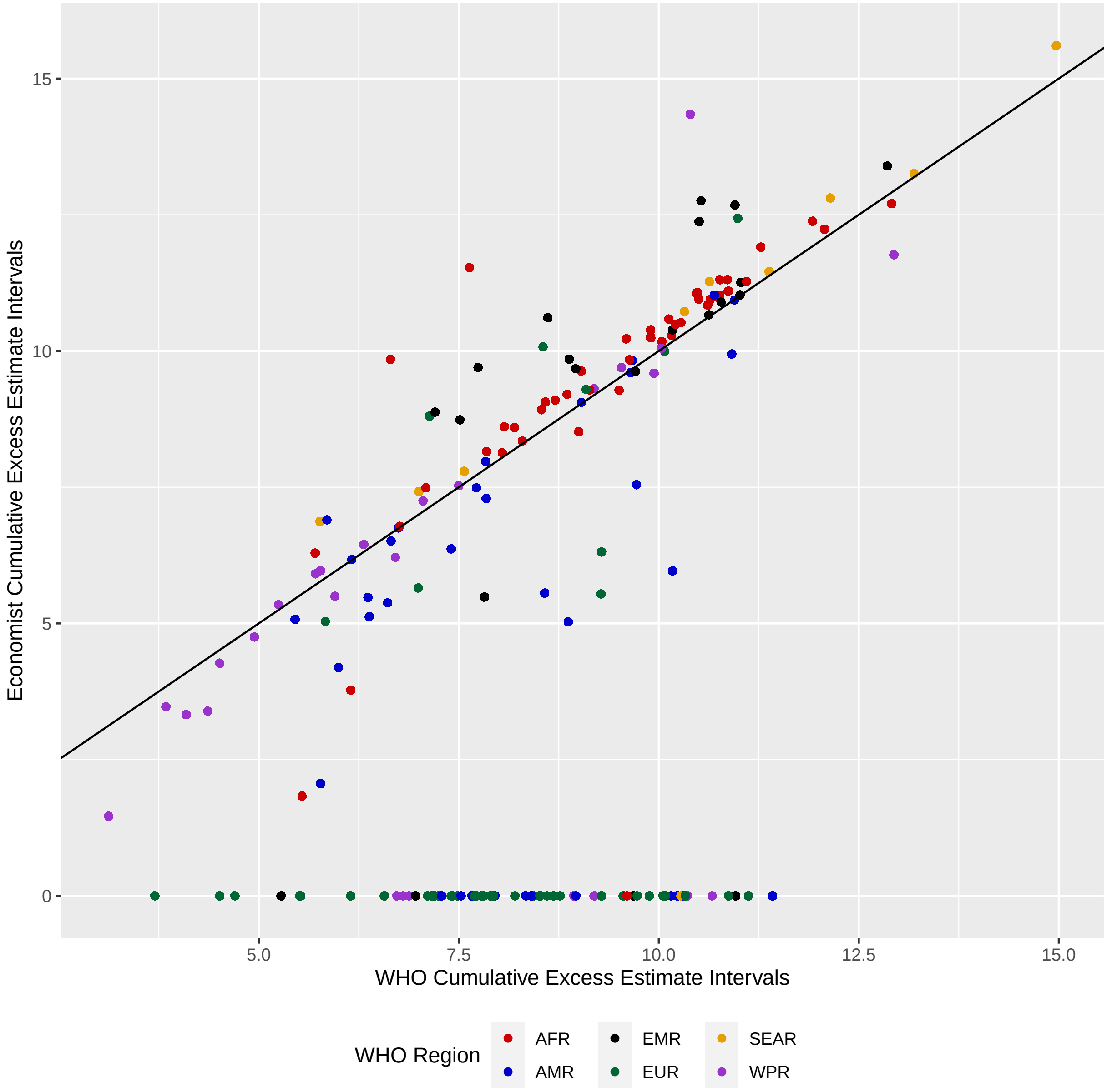

Supplementary Figure 4: 95% CI width of cumulative estimates of excess mortality for The Economist plotted against WHO, January 2020 to December 2021. Color-coded by WHO region.

Country rankings on excess mortality estimates

The ranking of countries according to some indicator is fraught with difficulties, some of which have been discussed in the context of COVID-19 mortality and the United Kingdom (Spiegelhalter, 2020).

We present an analysis of the excess mortality rates for the UK as compared to the 27 countries of the European Union (EU). The relative ranking of the UK as compared to the EU has been the subject of much political debate. For example, from Reuters (2020) on April 29, 2020: "The United Kingdom's COVID-19 death toll is probably higher than 27,241, making it one of the worst-hit countries in Europe, Labour Party leader Keir Starmer said on Wednesday as he questioned the government's response to the outbreak... We are possibly on track to have one of the worst death rates in Europe," Starmer told parliament. "Far from success, these latest figures are truly dreadful."

More recently, these claims have been questioned. For example, on May 25, 2022, at Prime Minister's question time, Boris Johnson (UK Prime Minister), in an exchange with Keir Starmer, said, "How many times did he come to this place and say that the United Kingdom had the highest covid death rate in Europe? How many times? He was proved completely wrong. Did he ever apologise? Absolutely not." (May 25th, 2022 Whole Day content downloaded from `https://hansard.parliament.uk/commons/`). And on June 8 2022, Johnson said, "By the way, he continually came to this House – I will just remind him of this – and said that we had the worst covid record in Europe. It turned out to be completely untrue; but he still has not retracted it." (June 8th, 2022 Whole Day content downloaded from `https://hansard.parliament.uk/commons/`). Misleading ranking analyses have also been reported in the mainline media (Boyd et al., 2022).

So an interesting question is, what was the rate of excess mortality in the UK, as compared to countries in the EU. In the above quotes and articles, the cumulative situation is being referred to, but we need to examine the situation dynamically over time. Ignoring the time aspect we first collapse across 2020 and 2021 and examine the rankings. Figure 5 shows the posterior summaries for the excess mortality rate for each of the 28 countries (left), along with the bivariate comparisons between the countries (right). The countries are ordered from top to bottom in terms of decreasing posterior mean (the use of the posterior median gave the same ordering). From this, we might conclude that, in terms of the excess rate, the UK was "in the middle of the pack". However, if we examine the rankings over time a more nuanced story emerges.

In the top panel of Figure 6 we show the point estimate (posterior mean) of the excess mortality, by month, while in the bottom panel, we show the probabilities of the UK falling in rank position 1, 2, etc; we go down to 16 only (rather than the full number of 28), since the non-zero probabilities are spread across the first 16 positions. Numerical summarizes are given in Table 3. While one should not over-interpret the specific values, it is clear that the UK had a relatively high excess rate, as compared to countries in the EU, until October of 2020.

**Month**	**Top 1**	**Top 2**	**Top 3**	**Top 4**	**Top 5**
January	0.471	0.727	0.892	0.967	0.992
February	0.322	0.565	0.728	0.840	0.918
March	0.000	0.000	0.362	0.618	0.745
April	0.000	0.541	0.998	1.000	1.000
May	0.288	0.996	1.000	1.000	1.000
June	0.512	0.994	1.000	1.000	1.000
July	0.292	0.992	1.000	1.000	1.000
August	0.087	0.982	1.000	1.000	1.000
September	0.009	0.953	1.000	1.000	1.000
October	0.000	0.660	0.937	0.996	1.000
November	0.000	0.000	0.002	0.015	0.058
December	0.000	0.000	0.000	0.000	0.000

Supplementary Table 3: Percent of estimates in which the UK is in the top rankings of cumulative excess rate among European countries in 2020.

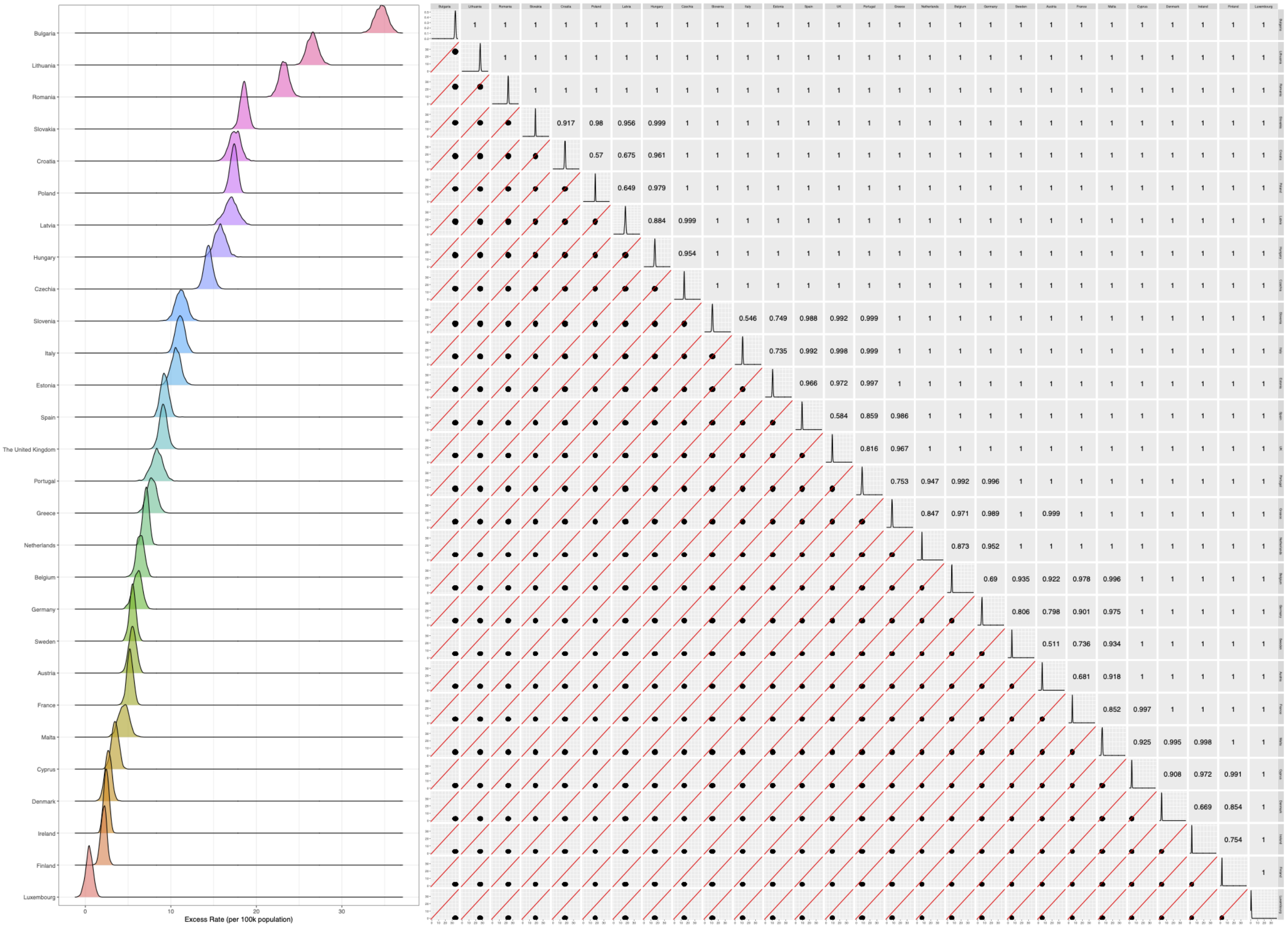

Supplementary Figure 5: Left: Ridgeplots representing the uncertainty in the cumulative excess monthly mortality rate over January 2020–December 2021 for all EU countries and the UK. Right: Bivariate plots of pairs of excess rates (lower triangular), 1-dimensional summaries for individual countries (diagonal), and probabilities that the excess for the country labeled on the left exceeds the rate for the country labeled at the top. These probabilities are the fraction of points that lie below the red line in the corresponding bivariate plot.

For completeness, Figure 7 gives the full set of ranking distributions across time. Bulgaria clearly has the highest rates among the 28 countries for the majority of 2021.

We could also rank using the P-Score (ratio of excess to expected), which takes some account of the age-sex structure of the population. In the top panel of Figure 8 we show the point estimate of the P-Scores by month, for each of the 28 countries being considered. It is quite similar to the excess rate version in Figure 6, but there are differences. Figure 9 shows the marginal P-Scores (left) and bivariate summaries for the 28 countries and Figure 10 shows the full set of ranking distributions across time, all with respect to the P-Score.

If we naively rank in terms of a point estimate (posterior means) then the UK is 12th in terms of the P-Score and 14th for the excess rate. The UK jumps above Spain (0.53 versus 0.42) because the median age in UK in 2020 is 39.5 years but 43.5 years in Spain[1].

In an ideal world we would be able to critically evaluate the excess mortality rates of different countries to try to determine prevention and control strategies that worked, and policies that were inadequate. This is unfortunately very difficult. But in House of Commons (2021) it was acknowledged that the UK's COVID-19 response in the first few months of the pandemic was inadequate. For example, from the Executive Summary: "In the first three months the strategy reflected official scientific advice to the Government which was accepted and implemented. When the Government moved from the contain stage to the delay stage, that approach involved trying to manage the spread of covid through the population rather than to stop it spreading altogether. This amounted in practice to accepting that herd immunity by infection was the inevitable outcome, given that the United Kingdom had no firm prospect of a vaccine, limited testing capacity and there was a widespread view that the public would not accept a lockdown for a significant period".

We end with a number of caveats. The analysis we have presented is only as good as the data we have access to, and the appropriateness of the models we have fitted. The mortality data varies in quality by country. In terms of modeling, we have observed mortality data for all countries, though some completeness assumptions need to be made. The excess calculation depends fundamentally on the model for the expected numbers, and different models/time periods for the expected modeling may lead to very different rankings. We have not considered the age-sex distribution of the excess (or at least only directly via examination of the P-Score), which is a crucial element, since countries have very different

[1] https://population.un.org/wpp/

demographic profiles, and mortality has a very strong association with age in particular. Age-sex modeling is not the subject of this paper, but for the countries we have considered this would be a viable option, since the data are in general of high quality.

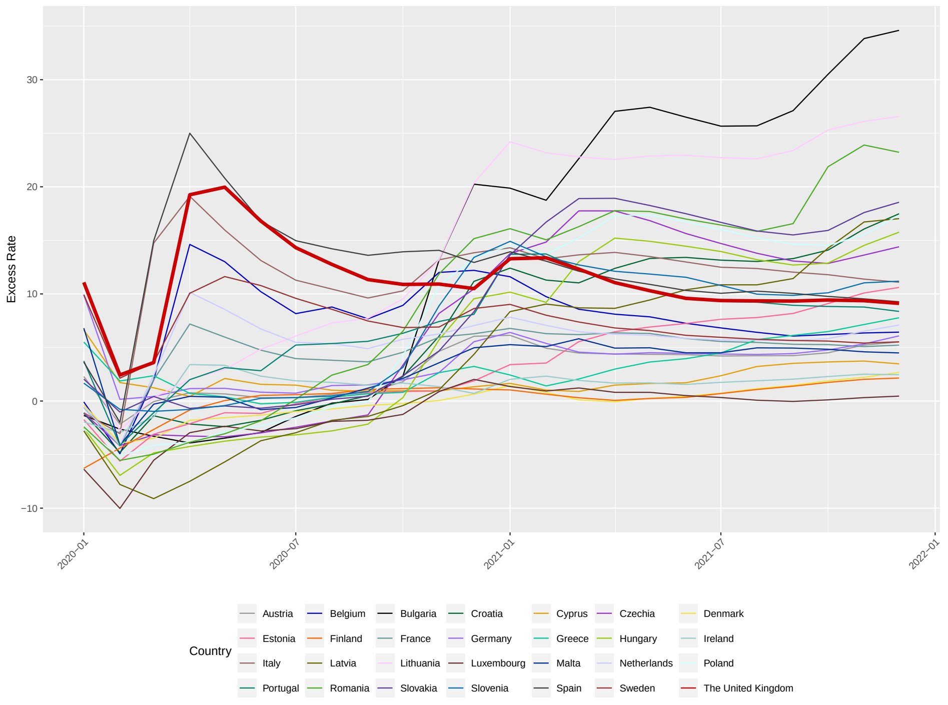

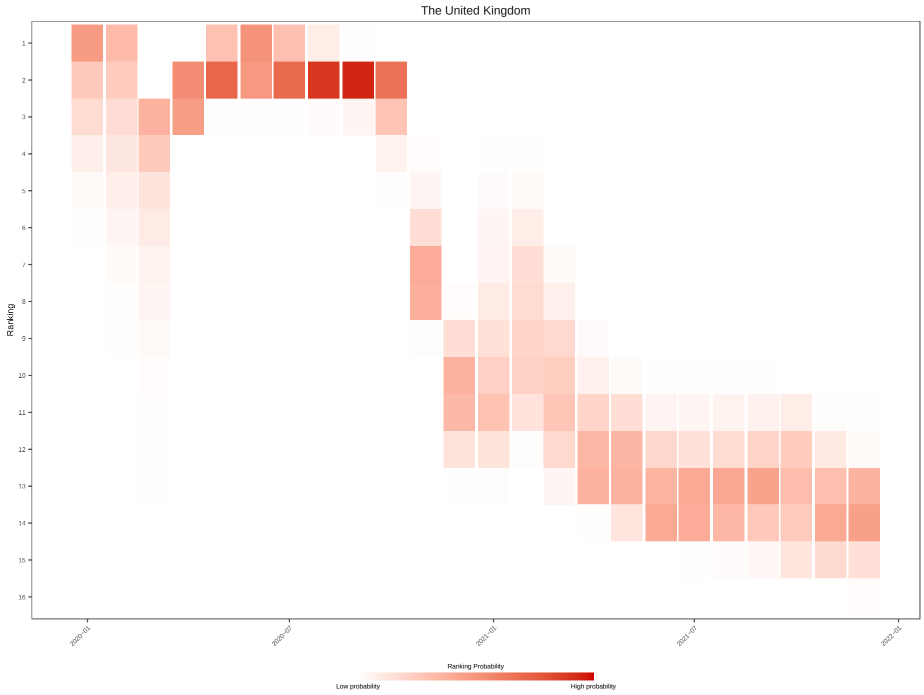

Supplementary Figure 6: Top: Point estimate of excess mortality rate for 27 EU countries and the UK, by month. Bottom: Ranking distribution for the UK, with respect to the excess mortality rate.

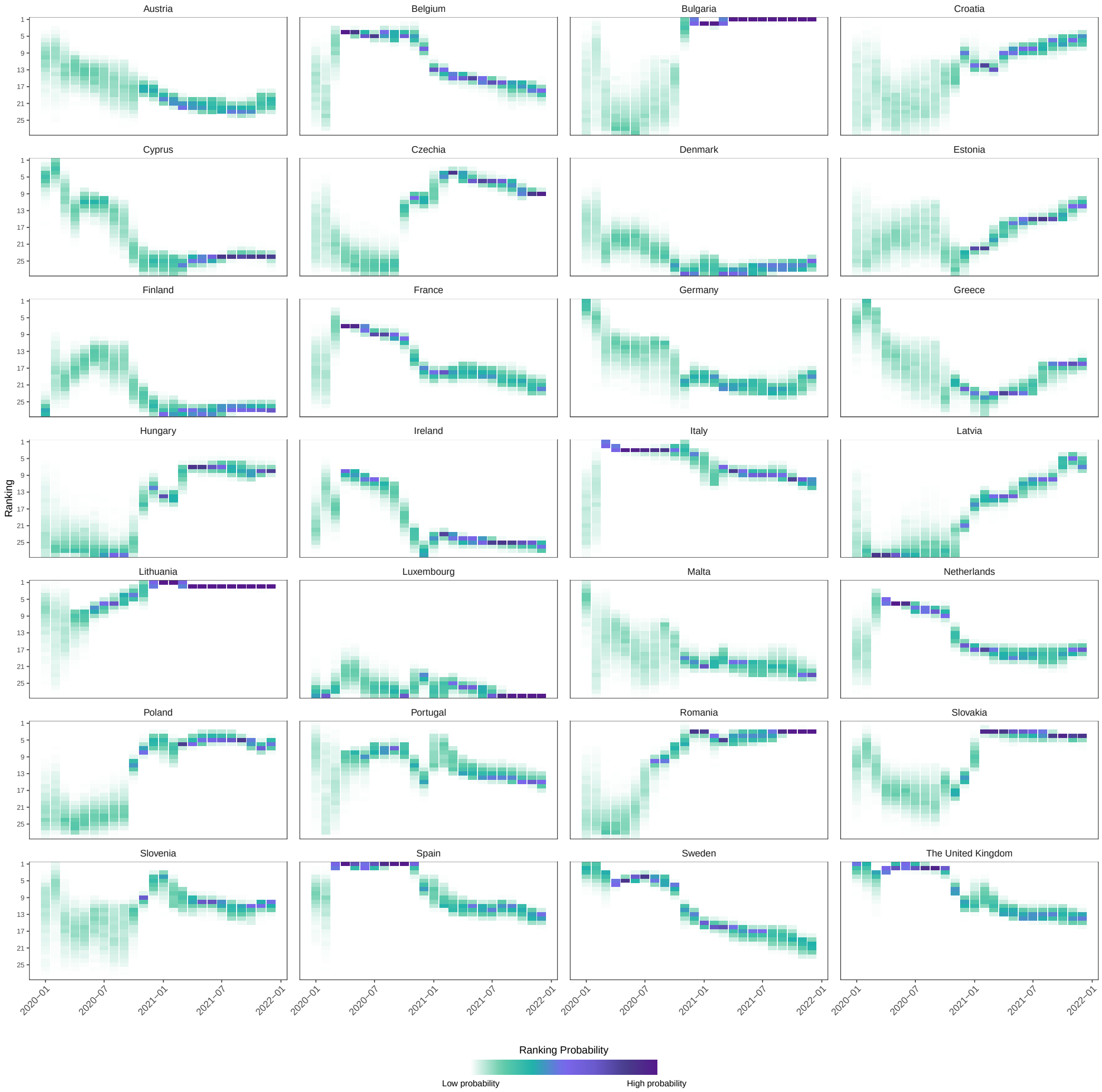

Supplementary Figure 7: Ranking distribution, with respect to the excess mortality rate, for 27 EU countries and the UK.

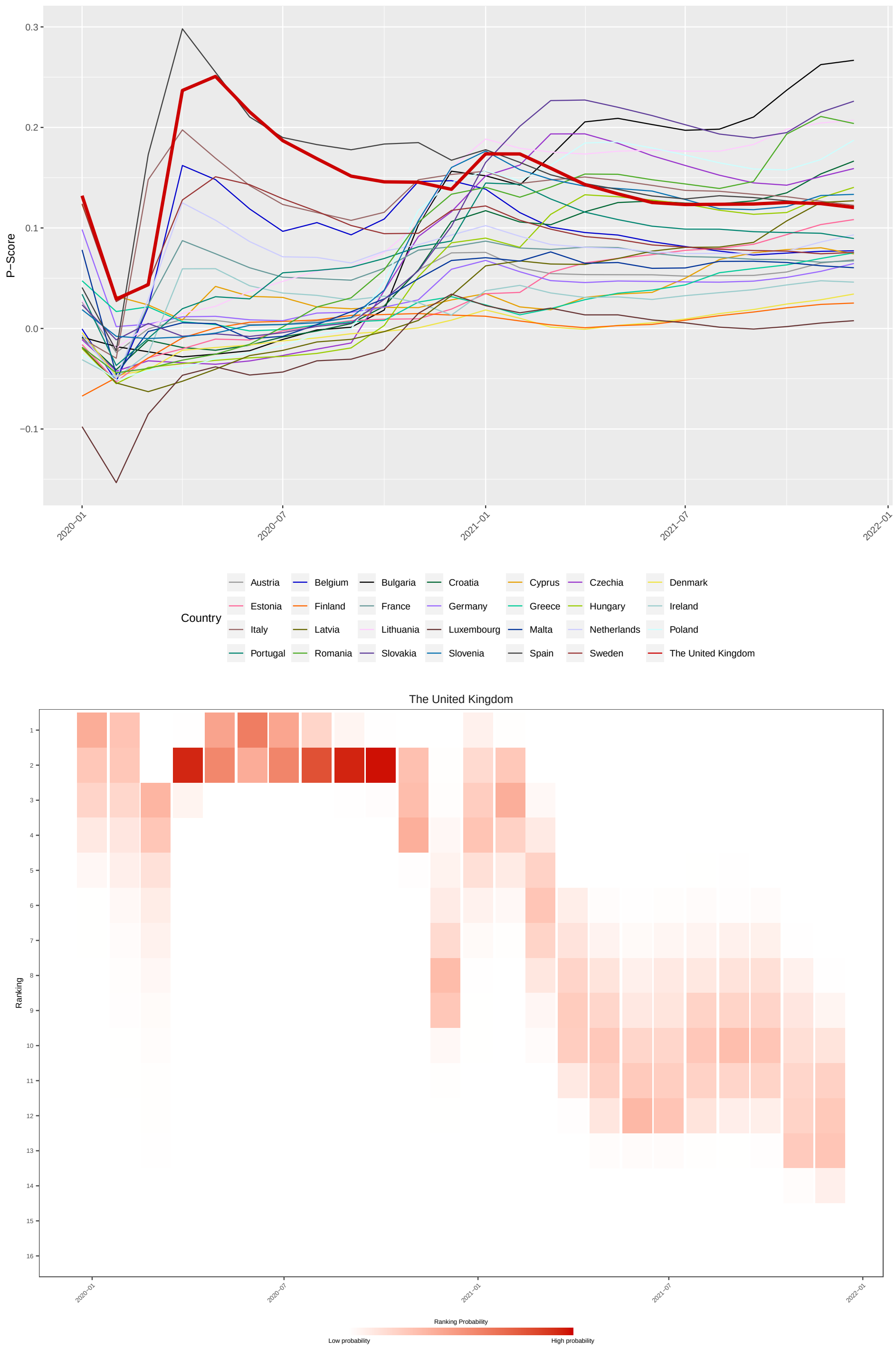

Supplementary Figure 8: Top: Point estimate of P-Score for 27 EU countries and the UK, by month. Bottom: Ranking distribution for the UK, with respect to the P-Score.

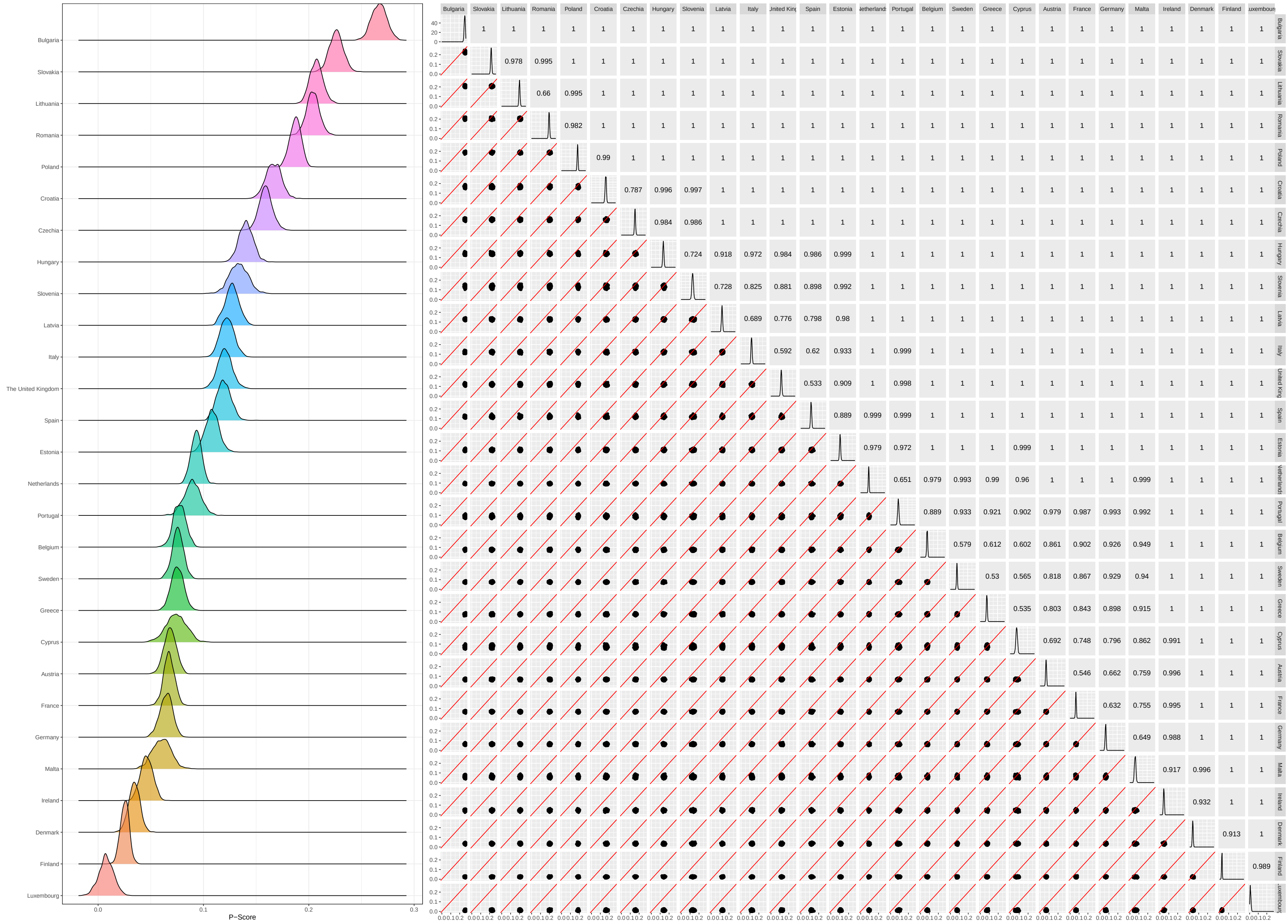

Supplementary Figure 9: Left: Ridgeplots representing the uncertainty in the cumulative excess monthly P-Score over January 2020–December 2021 for all EU countries and the UK. Right: Bivariate plots of pairs of excess rates (lower triangular), 1-dimensional summaries for individual countries (diagonal), and probabilities that the excess for the country labeled on the left exceeds the rate for the country labeled at the top. These probabilities are the fraction of points that lie below the red line in the corresponding bivariate plot.

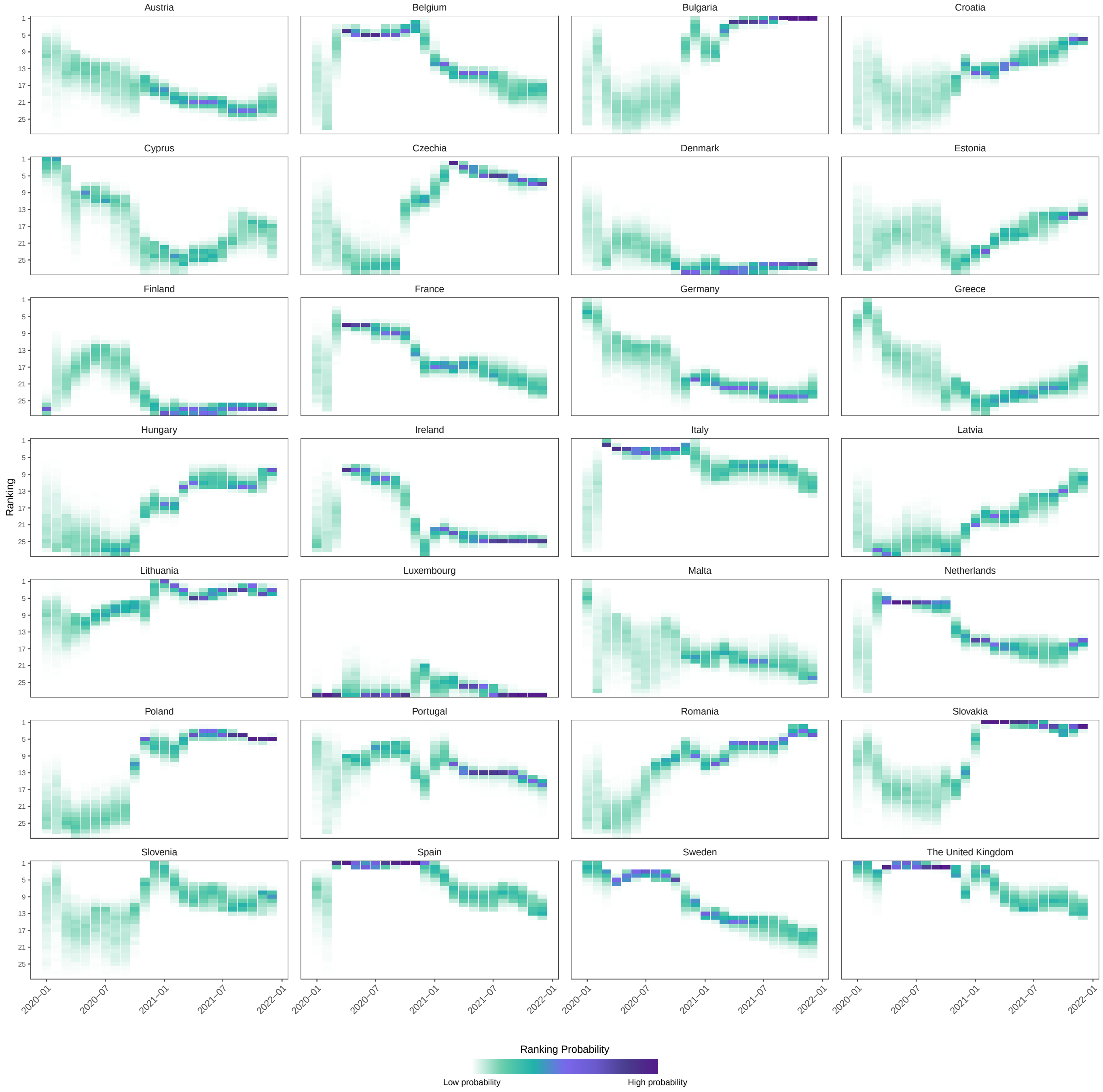

Supplementary Figure 10: Ranking distribution, with respect to the P-Score, for 27 EU countries and the UK.

Additional Analyses for Germany and Sweden

After the official release of the WHO results, there was attention on Germany and Sweden which has lead us to examine our models and data sources for those countries. The original excess estimate for Germany was 195K (UI 161K, 229K). This estimate was obtained using a negative binomial model with a thin-plate spline for the annual trend (on the linear predictor scale). A scaling factor to account for completeness was also used, which lead the ACM counts in 2016–2018 to be scaled up. Unfortunately for the adjusted data the spline fit was unduly influenced by a lower count in 2019 which caused the spline prediction in the pandemic to be too low, and the excess correspondingly to be too high. We removed the completeness adjustment and replaced the spline term for the annual trend with a linear term, and this produced the much more reasonable series in the left-hand panel of Figure 11. This produces a revised excess estimate of 122K (UI 101K, 143K). With the adjusted data, the spline also produced a reasonable fit (right-hand panel of Figure 11) and very similar estimates.

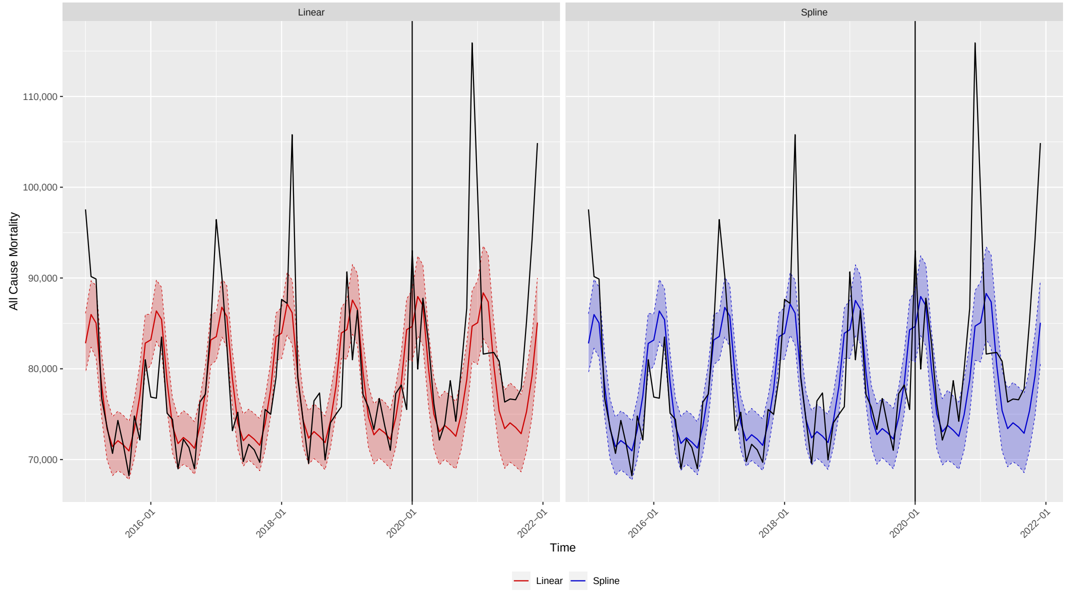

Supplementary Figure 11: Expected ACM counts and observed ACM (the black lines) for Germany, using unadjusted ACM data.

For Sweden, the WHO made a completeness adjustment for the 2019 mortality count (which was lower than the previous year), which resulted in an increase in the count, and

this same adjustment was also applied to the 2020 and 2021 counts. The original excess estimate for Sweden, using the adjusted data, was 11.3K (UI 9.9K, 12.7K). With hindsight the adjustment was unnecessary and so we present here an analysis with the unadjusted data, and replacing the spline term for the annual trend with a linear term. The revised estimates with the unadjusted data are 13.4K (UI 11.7K, 15.2K), so an increase over the previous analysis. With the unadjusted data the spline model gave similar excess estimates. The fits are shown in Figure 12.

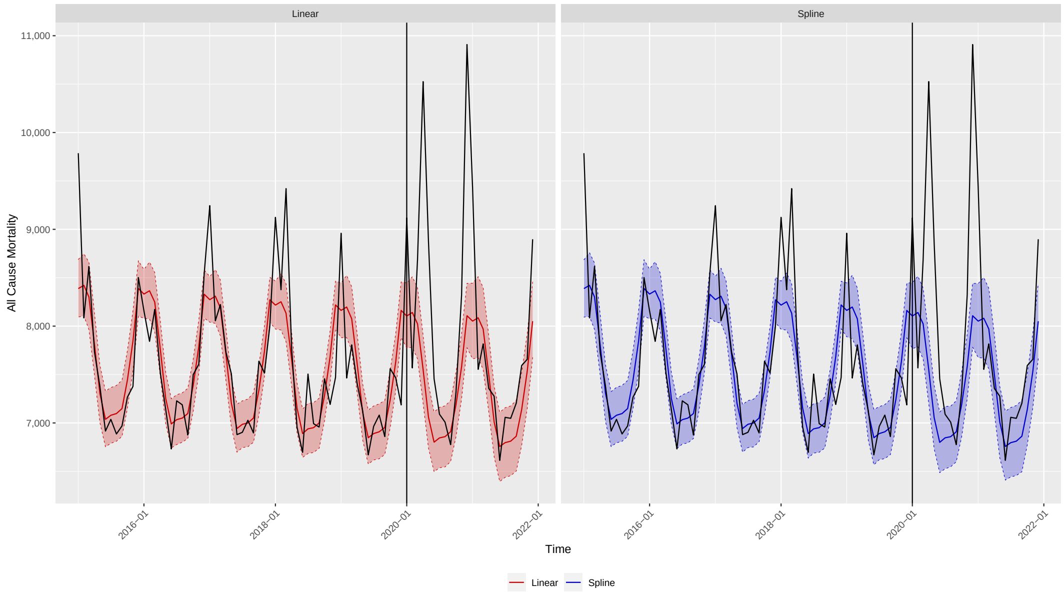

Supplementary Figure 12: Expected ACM counts and observed ACM (the black lines) for Sweden, using unadjusted ACM data.

For the next update of estimates, we will revisit the completeness process that was used to produce the counts used in the various analyses, and also examine different models for calculating the expected numbers.

Estimates for India

In Figure 13 we display in purple the states for which we use data in the subnational analysis.

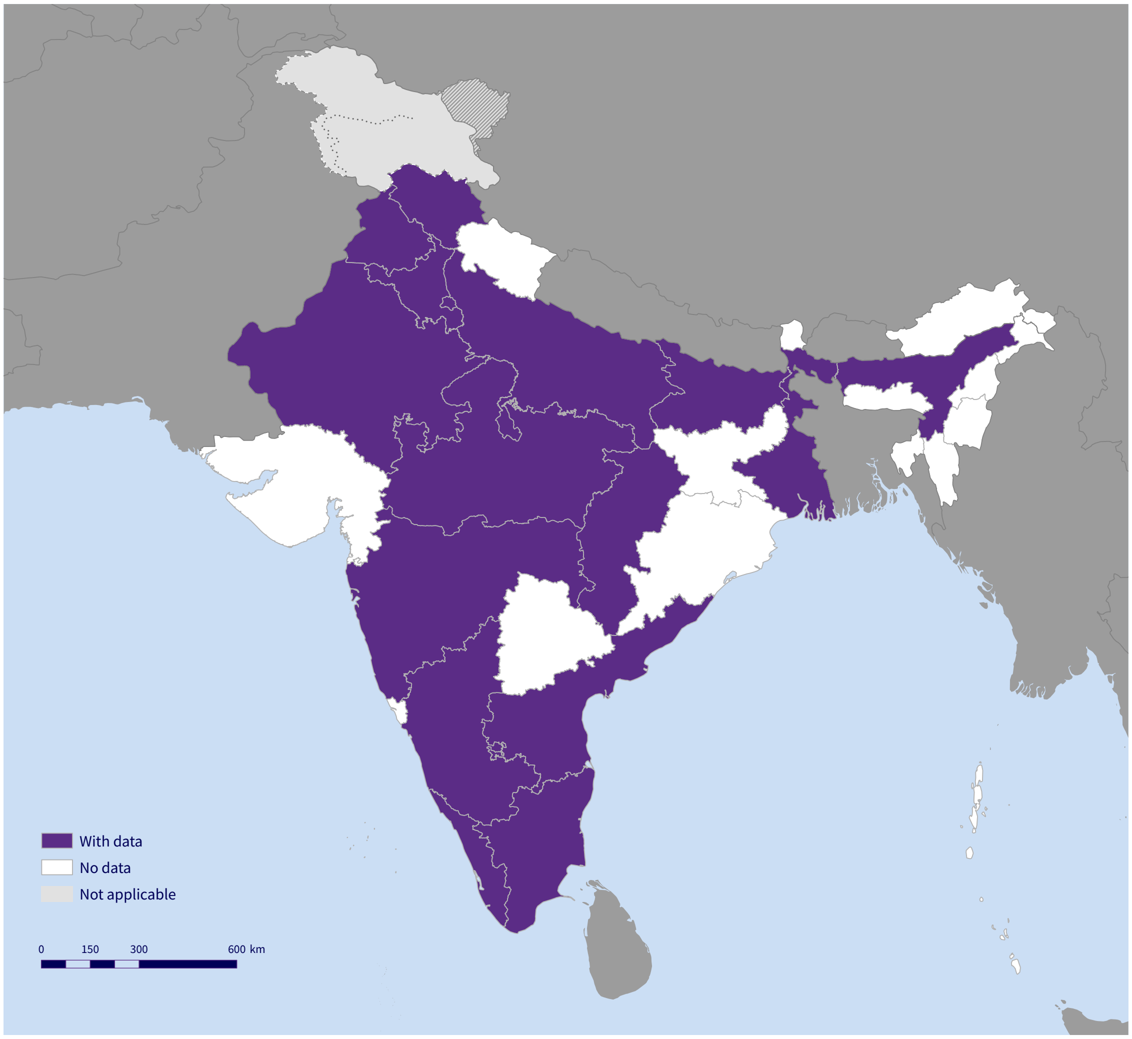

Supplementary Figure 13: States colored purple denote those that have data which we use for the subnational analysis.

Figure 14 shows the cumulative excess over January 2020–December 2021, along with the estimated contribution from the state-level data only.

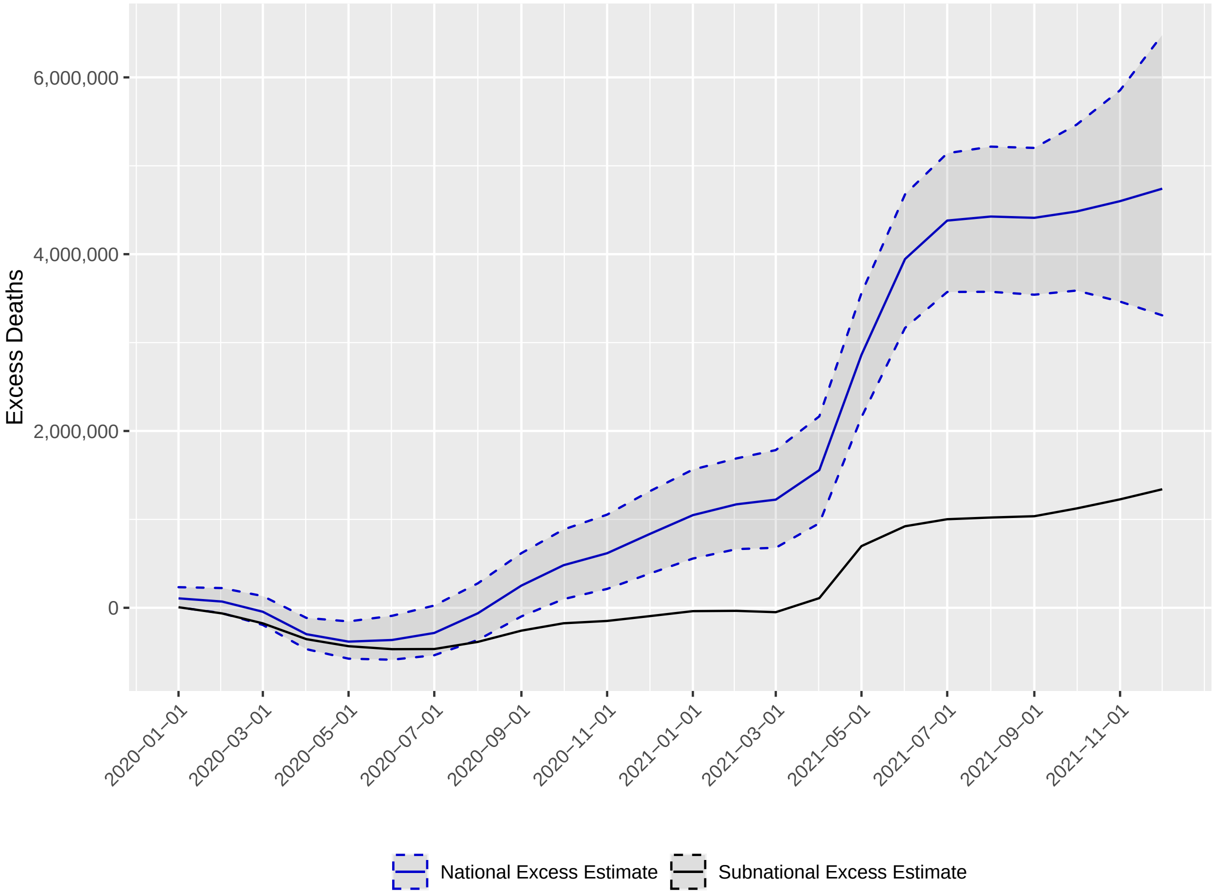

Supplementary Figure 14: Cumulative excess over January 2020 to December 2021 for India. The black curve is the contribution from the observed state level data.

In Table 4 we give our estimates (based on all data for the first 21 months and with expected numbers calculated from 2019 only) and also give the estimates from IHME, The Economist, Jha et al. (2022) and three estimates from Anand et al. (2021). The Jha et al. (2022) estimate is based on a nationally representative telephone survey, a government survey that covers 0.14 million adults and the Government of India's data from facility-based deaths and CRS deaths in 10 states. Anand et al. (2021) use three methods with different data sources: Indian States' CRS (method 1), international age-specific infection fatality rates applied to Indian demography (method 2) and seroprevalence and a household survey (method 3).

There is reasonable agreement between the different estimates, which is reassuring, given the disparate data sources used. Along with the sensitivity and leave-one-out analyses contained in the Supplementary Materials of Knutson et al. (2023), this provides further evidence that the model is reasonable and the subnational data (taken as a whole) are representative, so that, when combined, they provide a reliable excess mortality estimate.

**Approach**	**Estimate** ($\times 10^6$)	Period
Naive	5.04 (4.48, 5.59)	Jan 20–Dec 21
WHO	4.74 (3.31, 6.48)	Jan 20–Dec 21
The Economist	4.86 (1.70, 8.47)	Jan 20–Dec 21
IHME	4.07 (3.71, 4.36)	Jan 20–Dec 21
Naive	4.29 (4.00, 4.59)	June 20–June 21
WHO	4.33 (2.85, 6.13)	June 20–June 21
Jha et al. (2022)	3.23 (3.06, 3.39)	June 20–June 21
Naive	3.96 (3.62, 4.29)	April 20–June 21
WHO	3.99 (2.40, 5.95)	April 20–June 21
Anand et al. (2021) Method 1	3.4	April 20–June 21
Anand et al. (2021) Method 2	4.0	April 20–June 21
Anand et al. (2021) Method 3	4.9	April 20–June 21

Supplementary Table 4: Estimates of excess deaths in India according to different sources. The parentheses give 95% uncertainty intervals. The Jha et al. (2022) estimate is for excess COVID-19 deaths. The naive estimates are based on the ACM estimates $Y_{t,1}/\widehat{p}_t$ where $Y_{t,1}$ is the observed ACM from the available states, and $\widehat{p}_t$ is the estimated fraction of deaths available in month t.

Tables of annual estimates of excess mortality, reported COVID-19, and P-Score by country

Country	ISO3	Year	Data Type	Income Group	WHO Region	Reported COVID	Excess	P-Score
Afghanistan	AFG	2020	No Data	LIC	EMR	2189.00	19392.46 (-1297.47, 40224.27)	7.59 (-0.51, 15.76)
Afghanistan	AFG	2021	No Data	LIC	EMR	5167.00	25140.34 (3443.2, 47993.63)	9.82 (1.35, 18.75)
Albania	ALB	2020	Full National	UMIC	EUR	1174.00	5567.98 (4876.73, 6295.28)	16.57 (14.21, 19.13)
Albania	ALB	2021	Full National	UMIC	EUR	2041.00	7120.69 (6223.95, 7937.42)	19.63 (16.73, 22.36)
Algeria	DZA	2020	Full National	LMIC	AFR	2751.00	55860.88 (53091.36, 58723.35)	26.84 (25.17, 28.61)
Algeria	DZA	2021	No Data	LMIC	AFR	3520.00	13726.18 (-5840.28, 34371.58)	6.45 (-2.77, 16.15)
Andorra	AND	2020	Full National	HIC	EUR	84.00	231.6 (204.55, 260.55)	36.75 (31.06, 43.25)
Andorra	AND	2021	No Data	HIC	EUR	56.00	143 (-16.7, 321.47)	22.09 (-2.55, 49.99)
Angola	AGO	2020	No Data	LMIC	AFR	405.00	5339.37 (-11074.3, 23140.95)	2.33 (-4.83, 10.1)
Angola	AGO	2021	No Data	LMIC	AFR	1352.00	17345.64 (-536.4, 37257.87)	7.55 (-0.23, 16.22)
Antigua and Barbuda	ATG	2020	Full National	HIC	AMR	5.00	-56.97 (-81.46, -33.21)	-8.57 (-11.87, -5.2)
Antigua and Barbuda	ATG	2021	No Data	HIC	AMR	113.00	27.07 (-125.9, 189.07)	4.06 (-18.33, 28.25)
Argentina	ARG	2020	Full National	UMIC	AMR	43018.00	31691.84 (23826.03, 38985.39)	8.81 (6.47, 11.05)
Argentina	ARG	2021	Subnational Data	UMIC	AMR	74093.00	58190.5 (31803.52, 85450.27)	15.58 (8.53, 22.89)
Armenia	ARM	2020	Full National	UMIC	EUR	2823.00	9918.39 (9514.98, 10340.19)	38.98 (36.8, 41.31)
Armenia	ARM	2021	Full National	UMIC	EUR	5149.00	9744.83 (9252.39, 10209.4)	39.16 (36.45, 41.79)
Australia	AUS	2020	Full National	HIC	WPR	909.00	-7655.39 (-9813.88, -5700.7)	-4.5 (-5.7, -3.39)
Australia	AUS	2021	Full National	HIC	WPR	1317.00	-6599.18 (-9834.11, -3594.2)	-3.76 (-5.51, -2.09)
Austria	AUT	2020	Full National	HIC	EUR	6307.00	6534.57 (5347.36, 7680.3)	7.52 (6.07, 8.95)
Austria	AUT	2021	Full National	HIC	EUR	7063.00	5403.86 (3931.19, 6826.42)	6.16 (4.41, 7.91)
Azerbaijan	AZE	2020	Full National	UMIC	EUR	2575.00	27577.81 (26076.1, 29112.8)	33.42 (31.02, 35.93)
Azerbaijan	AZE	2021	Full National	UMIC	EUR	5771.00	29430.62 (27412.79, 31378.04)	35.7 (32.44, 38.96)
Bahamas	BHS	2020	No Data	HIC	AMR	170.00	335.2 (61.66, 616.43)	12.4 (2.27, 22.8)
Bahamas	BHS	2021	No Data	HIC	AMR	546.00	667.44 (367.36, 1003.25)	24 (13.11, 36)
Bahrain	BHR	2020	No Data	HIC	EMR	352.00	246.16 (-146.12, 675.88)	4.94 (-2.92, 13.66)
Bahrain	BHR	2021	No Data	HIC	EMR	1042.00	419.69 (-72.64, 924.18)	7.82 (-1.33, 17.17)
Bangladesh	BGD	2020	No Data	LMIC	SEAR	7559.00	46041.31 (-15763.1, 111409.83)	5.69 (-1.95, 13.78)
Bangladesh	BGD	2021	No Data	LMIC	SEAR	20513.00	94723.04 (27298.98, 170505.86)	11.5 (3.29, 20.67)
Barbados	BRB	2020	Full National	HIC	AMR	7.00	-259.93 (-352.03, -167.06)	-7.49 (-9.9, -4.96)
Barbados	BRB	2021	Partial National	HIC	AMR	253.00	-95.95 (-345.22, 216.24)	-2.67 (-9.49, 6.16)
Belarus	BLR	2020	Full National	UMIC	EUR	1414.00	23914.65 (22372.37, 25504.15)	19.83 (18.31, 21.43)
Belarus	BLR	2021	Partial National	UMIC	EUR	4147.00	24977.98 (14372.48, 37383.91)	20.68 (11.7, 31.16)
Belgium	BEL	2020	Full National	HIC	EUR	19758.00	16968.06 (15300.2, 18632.79)	14.7 (13.06, 16.38)
Belgium	BEL	2021	Full National	HIC	EUR	8629.00	950.15 (-925.78, 2841.11)	0.82 (-0.78, 2.48)
Belize	BLZ	2020	Full National	LMIC	AMR	241.00	122.15 (88.41, 156.03)	6.01 (4.27, 7.79)
Belize	BLZ	2021	No Data	LMIC	AMR	356.00	574.98 (209.09, 980.91)	27.58 (10.14, 46.83)

Supplementary Table 5: Total reported COVID-19 and excess mortality measures for years 2020 and 2021. The measures shown are excess deaths and P-Scores by country and year. Country-specific information shown are the data availability type, World Bank income grouping and the WHO region.

Country	ISO3	Year	Data Type	Income Group	WHO Region	Reported COVID	Excess	P-Score
Benin	BEN	2020	No Data	LMIC	AFR	44.00	3933.14 (-3354.13, 11318.94)	4.19 (-3.57, 12.05)
Benin	BEN	2021	No Data	LMIC	AFR	117.00	7920.31 (199.25, 16866.73)	8.39 (0.21, 17.85)
Bhutan	BTN	2020	No Data	LMIC	SEAR	0.00	-211.85 (-595.86, 150.98)	-4.57 (-12.85, 3.26)
Bhutan	BTN	2021	No Data	LMIC	SEAR	3.00	-185.13 (-555.38, 225.91)	-3.9 (-11.8, 4.71)
Bolivia (Plurinational State of)	BOL	2020	Full National	LMIC	AMR	9135.00	36298.44 (35629.78, 36992.42)	40.85 (39.8, 41.96)
Bolivia (Plurinational State of)	BOL	2021	Full National	LMIC	AMR	10515.00	51731.99 (50971.08, 52545.59)	56.62 (55.33, 58.03)
Bosnia and Herzegovina	BIH	2020	Full National	UMIC	EUR	4050.00	5268.45 (4512.21, 6030.31)	13.77 (11.55, 16.07)
Bosnia and Herzegovina	BIH	2021	Full National	UMIC	EUR	9378.00	10447.59 (9296.81, 11532.52)	26.87 (23.2, 30.48)
Botswana	BWA	2020	No Data	UMIC	AFR	40.00	228.8 (-1436.18, 1858.56)	1.1 (-6.91, 8.97)
Botswana	BWA	2021	No Data	UMIC	AFR	2399.00	7183.84 (5110.93, 9321.12)	34.41 (24.44, 44.66)
Brazil	BRA	2020	Full National	UMIC	AMR	192681.00	210810.85 (202118.17, 218739.21)	15.09 (14.38, 15.74)
Brazil	BRA	2021	Full National	UMIC	AMR	426136.00	470456.44 (460272.23, 481068.28)	33.06 (32.11, 34.06)
Brunei Darussalam	BRN	2020	Full National	HIC	WPR	3.00	-122.89 (-174.2, -72.37)	-5.22 (-7.25, -3.15)
Brunei Darussalam	BRN	2021	No Data	HIC	WPR	54.00	12.8 (-379.81, 419.98)	0.53 (-15.18, 17.15)
Bulgaria	BGR	2020	Full National	UMIC	EUR	7515.00	16879.24 (15271.75, 18406.59)	15.64 (13.94, 17.3)
Bulgaria	BGR	2021	Full National	UMIC	EUR	23375.00	40614.91 (38934.23, 42357.22)	37.76 (35.63, 40.02)
Burkina Faso	BFA	2020	No Data	LIC	AFR	82.00	7172.88 (-4654.24, 18648.27)	4.72 (-3.06, 12.3)
Burkina Faso	BFA	2021	No Data	LIC	AFR	236.00	14986.64 (2327.94, 28391.68)	9.86 (1.53, 18.69)
Burundi	BDI	2020	No Data	LIC	AFR	2.00	4095.89 (-2477.49, 11037.17)	5.31 (-3.19, 14.34)
Burundi	BDI	2021	No Data	LIC	AFR	12.00	5241.55 (-1608.62, 12743.53)	6.75 (-2.06, 16.44)
Cabo Verde	CPV	2020	No Data	LMIC	AFR	112.00	327.82 (57.54, 623.4)	10.97 (1.91, 20.98)
Cabo Verde	CPV	2021	No Data	LMIC	AFR	240.00	598.81 (283.96, 915.48)	19.22 (9.03, 29.44)
Cambodia	KHM	2020	No Data	LMIC	WPR	0.00	637.67 (-7007.59, 8346.65)	0.65 (-7.14, 8.5)
Cambodia	KHM	2021	No Data	LMIC	WPR	3012.00	11880.08 (3540.83, 20752.53)	11.9 (3.54, 20.8)
Cameroon	CMR	2020	No Data	LMIC	AFR	448.00	12114.8 (-3374.11, 27534.29)	6.26 (-1.74, 14.2)
Cameroon	CMR	2021	No Data	LMIC	AFR	1403.00	23207.1 (6760.11, 39475.2)	12.16 (3.54, 20.69)
Canada	CAN	2020	Full National	HIC	AMR	15274.00	16809.33 (13858.64, 19961.64)	5.79 (4.72, 6.94)
Canada	CAN	2021	Partial National	HIC	AMR	14684.00	5209.91 (-8088.16, 19997.63)	1.78 (-2.74, 6.86)
Central African Republic	CAF	2020	No Data	LIC	AFR	63.00	2363.23 (-2046.53, 6800.67)	4.28 (-3.67, 12.35)
Central African Republic	CAF	2021	No Data	LIC	AFR	38.00	3883.24 (-577.63, 8864.8)	7.22 (-1.07, 16.44)
Chad	TCD	2020	No Data	LIC	AFR	104.00	6988.79 (-4949.82, 18966.98)	4.69 (-3.33, 12.75)
Chad	TCD	2021	No Data	LIC	AFR	77.00	12260.65 (-1007.68, 26605.52)	8.23 (-0.68, 17.9)
Chile	CHL	2020	Full National	HIC	AMR	16499.00	14575.46 (13608.36, 15514.95)	13.06 (12.09, 14.02)
Chile	CHL	2021	Full National	HIC	AMR	22597.00	24119.93 (22967.51, 25190.36)	21.25 (20.02, 22.4)
China	CHN	2020	Annual Data	UMIC	WPR	4788.00	-75524.91 (-85733.91, -66109.51)	-0.75 (-0.85, -0.66)
China	CHN	2021	Annual Data	UMIC	WPR	911.00	23462.18 (11165.22, 36447.91)	0.23 (0.11, 0.36)
Colombia	COL	2020	Full National	UMIC	AMR	42620.00	54060.44 (52591.06, 55387.16)	21.83 (21.11, 22.49)
Colombia	COL	2021	Full National	UMIC	AMR	87246.00	110683.86 (109195.84, 112256.24)	43.64 (42.8, 44.54)
Comoros	COM	2020	No Data	LMIC	AFR	9.00	156.47 (-242.04, 562.07)	3.1 (-4.78, 11.17)
Comoros	COM	2021	No Data	LMIC	AFR	149.00	567.01 (132.33, 1049.79)	11.17 (2.61, 20.68)
Congo	COG	2020	No Data	LMIC	AFR	100.00	1342.91 (-1214.11, 4061.11)	3.81 (-3.44, 11.52)
Congo	COG	2021	No Data	LMIC	AFR	267.00	3186.31 (208.32, 6521.57)	8.97 (0.59, 18.33)
Cook Islands	COK	2020	No Data	UMIC	WPR	0.00	-18.91 (-45.2, 10.88)	-11.48 (-27.77, 6.63)
Cook Islands	COK	2021	No Data	UMIC	WPR	0.00	-16.87 (-44.8, 12.65)	-10.05 (-26.47, 7.56)
Costa Rica	CRI	2020	Full National	UMIC	AMR	2156.00	1356.99 (967.09, 1754.03)	5.47 (3.83, 7.17)
Costa Rica	CRI	2021	No Data	UMIC	AMR	5198.00	8290.03 (4949.11, 11938.86)	32.68 (19.55, 47.38)
Cote d'Ivoire	CIV	2020	No Data	LMIC	AFR	137.00	7901.86 (-6885.58, 23417.71)	4.09 (-3.57, 12.11)
Cote d'Ivoire	CIV	2021	No Data	LMIC	AFR	575.00	17531.77 (1329.61, 33607.09)	9.18 (0.7, 17.58)

Supplementary Table 6: Total reported COVID-19 and excess mortality measures for years 2020 and 2021. The measures shown are excess deaths and P-Scores by country and year. Country-specific information shown are the data availability type, World Bank income grouping and the WHO region.

Country	ISO3	Year	Data Type	Income Group	WHO Region	Reported COVID	Excess	P-Score
Croatia	HRV	2020	Full National	HIC	EUR	3860.00	5502.92 (4614.66, 6319.62)	10.64 (8.77, 12.41)
Croatia	HRV	2021	Full National	HIC	EUR	8633.00	11673.19 (10719.32, 12617.77)	22.72 (20.47, 25)
Cuba	CUB	2020	Full National	UMIC	AMR	145.00	-233.78 (-1685.47, 1135.02)	-0.2 (-1.45, 1)
Cuba	CUB	2021	No Data	UMIC	AMR	8177.00	18393.56 (6006.8, 31904.33)	15.61 (5.1, 27.27)
Cyprus	CYP	2020	Full National	HIC	EUR	122.00	192.68 (50.77, 336.48)	2.84 (0.73, 5.05)
Cyprus	CYP	2021	Full National	HIC	EUR	524.00	816.06 (616.87, 1002.64)	12.01 (8.8, 15.14)
Czechia	CZE	2020	Full National	HIC	EUR	11887.00	13448.03 (11909.86, 14960.46)	11.61 (10.15, 13.09)
Czechia	CZE	2021	Full National	HIC	EUR	24398.00	23590.51 (21737.62, 25591.8)	20.18 (18.29, 22.26)
Democratic People's Republic of Korea	PRK	2020	No Data	LIC	SEAR	0.00	-5611.44 (-23663.34, 13876.16)	-2.44 (-10.27, 6.07)
Democratic People's Republic of Korea	PRK	2021	No Data	LIC	SEAR	0.00	-1486.91 (-22078.56, 20639.04)	-0.6 (-9.4, 9.18)
Democratic Republic of the Congo	COD	2020	No Data	LIC	AFR	584.00	37211.09 (-15942.25, 93901)	5.15 (-2.21, 12.98)
Democratic Republic of the Congo	COD	2021	No Data	LIC	AFR	621.00	80546.62 (20072.72, 144378.84)	11.13 (2.77, 19.92)
Denmark	DNK	2020	Full National	HIC	EUR	1319.00	449.42 (-174.63, 1059.07)	0.83 (-0.32, 1.97)
Denmark	DNK	2021	Full National	HIC	EUR	1938.00	3266.84 (2374.48, 4170)	6.05 (4.32, 7.84)
Djibouti	DJI	2020	No Data	LMIC	EMR	61.00	703.4 (118.3, 1360.56)	9.72 (1.64, 18.84)
Djibouti	DJI	2021	No Data	LMIC	EMR	128.00	1063.92 (441.48, 1757.57)	14.63 (6.07, 24.11)
Dominica	DMA	2020	No Data	UMIC	AMR	0.00	-54.59 (-130.04, 21.89)	-7.25 (-17.29, 2.92)
Dominica	DMA	2021	No Data	UMIC	AMR	45.00	69.64 (-18.99, 157.49)	9.2 (-2.5, 20.86)
Dominican Republic	DOM	2020	Partial National	UMIC	AMR	2409.00	1075.32 (-2023.9, 4626.07)	1.46 (-2.74, 6.22)
Dominican Republic	DOM	2021	No Data	UMIC	AMR	1837.00	10797.87 (3696.38, 18625.11)	14.41 (4.88, 24.79)
Ecuador	ECU	2020	Full National	UMIC	AMR	14023.00	46402.35 (45173.07, 47540.88)	57.92 (55.53, 60.19)
Ecuador	ECU	2021	Full National	UMIC	AMR	19646.00	34465.67 (32782.82, 36272.36)	43.19 (40.22, 46.49)
Egypt	EGY	2020	Full National	LMIC	EMR	7576.00	90949.01 (78345.28, 102751.24)	15.67 (13.2, 18.06)
Egypt	EGY	2021	Partial National	LMIC	EMR	14151.00	160152.56 (135305.03, 187606.24)	27.23 (22.49, 32.49)
El Salvador	SLV	2020	Partial National	LMIC	AMR	1327.00	7506.55 (4793.25, 10648.54)	17.71 (11.26, 25.36)
El Salvador	SLV	2021	No Data	LMIC	AMR	2496.00	9529.02 (3655.13, 16447.27)	22.44 (8.38, 38.67)
Equatorial Guinea	GNQ	2020	No Data	UMIC	AFR	86.00	695.34 (-154.92, 1630.93)	6.67 (-1.49, 15.66)
Equatorial Guinea	GNQ	2021	No Data	UMIC	AFR	89.00	1024.15 (147.36, 1927.33)	9.69 (1.4, 18.25)
Eritrea	ERI	2020	No Data	LIC	AFR	1.00	295.03 (-1774.83, 2316.53)	1.07 (-6.42, 8.38)
Eritrea	ERI	2021	No Data	LIC	AFR	74.00	2117.76 (-186.5, 4379.8)	7.72 (-0.68, 16)
Estonia	EST	2020	Full National	HIC	EUR	229.00	299.67 (72.69, 520.23)	1.93 (0.46, 3.39)
Estonia	EST	2021	Full National	HIC	EUR	1703.00	3074.76 (2786.8, 3369.27)	19.73 (17.55, 22.03)
Eswatini	SWZ	2020	No Data	LMIC	AFR	185.00	917.19 (-77.93, 1893.35)	7.92 (-0.67, 16.42)
Eswatini	SWZ	2021	No Data	LMIC	AFR	1114.00	2903.06 (1802.66, 4105.1)	25.62 (15.89, 36.43)
Ethiopia	ETH	2020	No Data	LIC	AFR	1918.00	27372.94 (-23353.37, 77334.17)	4.38 (-3.74, 12.38)
Ethiopia	ETH	2021	No Data	LIC	AFR	5008.00	76122.88 (22947.34, 126436.16)	12.2 (3.67, 20.28)
Fiji	FJI	2020	No Data	UMIC	WPR	2.00	-780.61 (-1306.07, -227.21)	-10.75 (-17.99, -3.14)
Fiji	FJI	2021	No Data	UMIC	WPR	696.00	663.69 (-30.35, 1392.36)	8.97 (-0.41, 18.84)
Finland	FIN	2020	Full National	HIC	EUR	592.00	746.98 (190.01, 1344.84)	1.33 (0.33, 2.42)
Finland	FIN	2021	Full National	HIC	EUR	1135.00	2110.02 (1441.41, 2722.2)	3.72 (2.51, 4.84)
France	FRA	2020	Full National	HIC	EUR	64004.00	49178.73 (41817.75, 56173.29)	8.12 (6.82, 9.38)
France	FRA	2021	Full National	HIC	EUR	56958.00	32671.26 (24098.68, 41499.51)	5.36 (3.89, 6.9)
Gabon	GAB	2020	No Data	UMIC	AFR	64.00	278.01 (-850.76, 1468.64)	1.94 (-5.95, 10.31)
Gabon	GAB	2021	No Data	UMIC	AFR	224.00	1324.04 (148.87, 2539.18)	9.23 (1.04, 17.74)
Gambia	GMB	2020	No Data	LIC	AFR	124.00	1000.59 (-150.65, 2271.64)	7.01 (-1.05, 15.92)
Gambia	GMB	2021	No Data	LIC	AFR	219.00	1903.59 (655.27, 3261.08)	13.26 (4.56, 22.76)

Supplementary Table 7: Total reported COVID-19 and excess mortality measures for years 2020 and 2021. The measures shown are excess deaths and P-Scores by country and year. Country-specific information shown are the data availability type, World Bank income grouping and the WHO region.

Country	ISO3	Year	Data Type	Income Group	WHO Region	Reported COVID	Excess	P-Score
Georgia	GEO	2020	Full National	UMIC	EUR	2505.00	5570.7 (4765.25, 6357.9)	10.67 (8.98, 12.36)
Georgia	GEO	2021	Partial National	UMIC	EUR	11295.00	18893.9 (14009.88, 24699.86)	36.64 (27.03, 48.36)
Germany	DEU	2020	Full National	HIC	EUR	33071.00	55648.09 (8027.71, 102189.2)	5.89 (4.27, 7.44)
Germany	DEU	2021	Full National	HIC	EUR	78854.00	66784.05 (12029.44, 120424.6)	6.46 (5.21, 7.56)
Ghana	GHA	2020	No Data	LMIC	AFR	335.00	6460.7 (-8605.77, 21248.07)	3.4 (-4.53, 11.18)
Ghana	GHA	2021	No Data	LMIC	AFR	952.00	14446.19 (-685.83, 29501.82)	7.62 (-0.36, 15.54)
Greece	GRC	2020	Full National	HIC	EUR	4788.00	4043.16 (2182.73, 5861.84)	3.16 (1.68, 4.64)
Greece	GRC	2021	Full National	HIC	EUR	15920.00	15350.83 (13324.26, 17518.6)	11.84 (10.11, 13.73)
Grenada	GRD	2020	Annual Data	UMIC	AMR	1.00	-253.35 (-276.33, -232.79)	-24.62 (-26.83, -22.6)
Grenada	GRD	2021	No Data	UMIC	AMR	199.00	-13.68 (-271.04, 292.77)	-1.29 (-25.76, 28.19)
Guatemala	GTM	2020	Full National	UMIC	AMR	4803.00	8748.55 (7987.92, 9559.58)	8.97 (8.13, 9.88)
Guatemala	GTM	2021	Full National	UMIC	AMR	11299.00	40647.14 (39640.82, 41596.3)	40.93 (39.51, 42.29)
Guinea	GIN	2020	No Data	LIC	AFR	80.00	3930.87 (-4603.79, 12868.15)	3.64 (-4.26, 11.89)
Guinea	GIN	2021	No Data	LIC	AFR	311.00	8598.8 (-276.29, 17696.33)	7.97 (-0.26, 16.4)
Guinea-Bissau	GNB	2020	No Data	LIC	AFR	45.00	1097.88 (-191.08, 2521.52)	6.8 (-1.18, 15.54)
Guinea-Bissau	GNB	2021	No Data	LIC	AFR	104.00	1743.28 (400.08, 3080.72)	10.83 (2.48, 19.14)
Guyana	GUY	2020	No Data	UMIC	AMR	164.00	395.68 (-256.96, 1080.05)	4.75 (-3.09, 12.96)
Guyana	GUY	2021	No Data	UMIC	AMR	887.00	2415.5 (1528.98, 3321.2)	27.89 (17.65, 38.29)
Haiti	HTI	2020	No Data	LMIC	AMR	236.00	2865.06 (-14992.02, 20056.71)	3.4 (-14.48, 23.16)
Haiti	HTI	2021	No Data	LMIC	AMR	530.00	6743.71 (-13735.06, 27688.93)	7.67 (-13.44, 33)
Honduras	HND	2020	No Data	LMIC	AMR	3111.00	7284.69 (2354.81, 12167.54)	12.98 (4.11, 21.77)
Honduras	HND	2021	No Data	LMIC	AMR	7323.00	15377.4 (9270.91, 21526.85)	26.72 (16, 37.78)
Hungary	HUN	2020	Full National	HIC	EUR	9537.00	11071.3 (9157.45, 13133.37)	8.52 (6.94, 10.26)
Hungary	HUN	2021	Full National	HIC	EUR	29649.00	25425.41 (23150.67, 27522.26)	19.57 (17.5, 21.52)
Iceland	ISL	2020	Full National	HIC	EUR	29.00	-2.57 (-37.85, 32.09)	-0.1 (-1.56, 1.37)
Iceland	ISL	2021	Full National	HIC	EUR	8.00	-8.07 (-49.89, 36.35)	-0.33 (-2.03, 1.53)
India	IND	2020	Subnational Data	LMIC	SEAR	148738.00	832531.02 (384866.44, 1315711.87)	9.03 (4.16, 14.4)
India	IND	2021	Subnational Data	LMIC	SEAR	332342.00	3908362.5 (2672403.78, 5501911.64)	42.03 (28.59, 59.37)
Indonesia	IDN	2020	Subnational Data	LMIC	SEAR	22138.00	357987.87 (201047.49, 518748.96)	19.35 (10.86, 28.01)
Indonesia	IDN	2021	Subnational Data	LMIC	SEAR	121956.00	670577.19 (506117.54, 838433.43)	35.6 (26.81, 44.43)
Iran (Islamic Republic of)	IRN	2020	Full National	LMIC	EMR	55095.00	108918.59 (103976.59, 113417.77)	27.51 (25.94, 28.97)
Iran (Islamic Republic of)	IRN	2021	Full National	LMIC	EMR	76477.00	123234.78 (116246.95, 129565.67)	30.21 (28.01, 32.26)
Iraq	IRQ	2020	Full National	UMIC	EMR	12808.00	40399.73 (37410.32, 43198.32)	24.76 (22.5, 26.92)
Iraq	IRQ	2021	No Data	UMIC	EMR	11346.00	26334.88 (8606.83, 47117.47)	15.84 (5.18, 28.05)
Ireland	IRL	2020	Full National	HIC	EUR	2276.00	421.58 (-26.26, 846.71)	1.34 (-0.08, 2.72)
Ireland	IRL	2021	Full National	HIC	EUR	3798.00	2500.26 (2003.89, 3010.52)	7.86 (6.19, 9.6)
Israel	ISR	2020	Full National	HIC	EUR	3337.00	2431.73 (1823.56, 2991.96)	5.25 (3.89, 6.54)
Israel	ISR	2021	Full National	HIC	EUR	4920.00	3745.34 (2993.43, 4495.51)	7.97 (6.27, 9.72)
Italy	ITA	2020	Full National	HIC	EUR	73604.00	100431 (90654.76, 110827.23)	15.32 (13.62, 17.17)
Italy	ITA	2021	Full National	HIC	EUR	63643.00	60369.58 (48931.18, 72362.33)	9.19 (7.31, 11.21)
Jamaica	JAM	2020	Partial National	UMIC	AMR	302.00	-902.28 (-1353.42, -340.55)	-4.62 (-6.94, -1.75)
Jamaica	JAM	2021	No Data	UMIC	AMR	2168.00	4494.59 (2128.56, 7154.67)	22.74 (10.79, 36.06)
Japan	JPN	2020	Full National	HIC	WPR	3414.00	-30138.79 (-40681.71, -19990.84)	-2.13 (-2.85, -1.42)
Japan	JPN	2021	Full National	HIC	WPR	14979.00	10668.1 (-1651.96, 22689.77)	0.74 (-0.11, 1.59)
Jordan	JOR	2020	Full National	UMIC	EMR	3815.00	3640.28 (3057.1, 4186.84)	12.38 (10.18, 14.49)
Jordan	JOR	2021	No Data	UMIC	EMR	8805.00	8288.61 (4513.55, 12287.85)	27.7 (15.03, 41.01)
Kazakhstan	KAZ	2020	Full National	UMIC	EUR	2749.00	28494.29 (26982.35, 30003.26)	21.53 (20.15, 22.93)
Kazakhstan	KAZ	2021	Full National	UMIC	EUR	15478.00	47720.34 (45758.83, 49570.3)	36 (34.01, 37.92)

Supplementary Table 8: Total reported COVID-19 and excess mortality measures for years 2020 and 2021. The measures shown are excess deaths and P-Scores by country and year. Country-specific information shown are the data availability type, World Bank income grouping and the WHO region.

Country	ISO3	Year	Data Type	Income Group	WHO Region	Reported COVID	Excess	P-Score
Kenya	KEN	2020	Full National	LMIC	AFR	1667.00	-12514.25 (-13047.79, -11965.69)	-4.22 (-4.4, -4.05)
Kenya	KEN	2021	Full National	LMIC	AFR	3709.00	24865.17 (23985.97, 25754.47)	8.31 (8, 8.64)
Kiribati	KIR	2020	No Data	LMIC	WPR	0.00	-30.92 (-156.27, 101.11)	-2.35 (-11.87, 7.68)
Kiribati	KIR	2021	No Data	LMIC	WPR	0.00	-14.49 (-138.99, 115.98)	-1.08 (-10.4, 8.73)
Kuwait	KWT	2020	Full National	HIC	EMR	933.00	3177.32 (3048.04, 3298.69)	36.35 (34.35, 38.26)
Kuwait	KWT	2021	No Data	HIC	EMR	1535.00	1062.38 (-66.4, 2240.97)	11.42 (-0.71, 24.21)
Kyrgyzstan	KGZ	2020	Full National	LMIC	EUR	1355.00	6646.62 (6171.45, 7137.49)	19.95 (18.26, 21.73)
Kyrgyzstan	KGZ	2021	Full National	LMIC	EUR	1447.00	5666.37 (4939.52, 6383.03)	16.92 (14.42, 19.45)
Lao People's Democratic Republic	LAO	2020	No Data	LMIC	WPR	0.00	-274.5 (-3314.28, 3118.54)	-0.63 (-7.57, 7.11)
Lao People's Democratic Republic	LAO	2021	No Data	LMIC	WPR	360.00	2030.59 (-1197.29, 5707.43)	4.61 (-2.72, 13.01)
Latvia	LVA	2020	Full National	HIC	EUR	626.00	986.03 (551.39, 1394.68)	3.28 (1.81, 4.7)
Latvia	LVA	2021	Full National	HIC	EUR	3935.00	6682.21 (6141.91, 7214.18)	22.09 (19.94, 24.26)
Lebanon	LBN	2020	Full National	UMIC	EMR	1443.00	3412.92 (2601.51, 4228.08)	8.62 (6.43, 10.89)
Lebanon	LBN	2021	Full National	UMIC	EMR	7659.00	15107.18 (14044.28, 16066.77)	36.94 (33.46, 40.21)
Lesotho	LSO	2020	No Data	LMIC	AFR	50.00	201.25 (-2346.75, 2932.07)	0.56 (-6.56, 8.22)
Lesotho	LSO	2021	No Data	LMIC	AFR	615.00	3780.67 (760.48, 7025.88)	10.57 (2.12, 19.71)
Liberia	LBR	2020	No Data	LIC	AFR	83.00	1353.9 (-1820.53, 4492.29)	3.75 (-5.03, 12.7)
Liberia	LBR	2021	No Data	LIC	AFR	204.00	2689.37 (-502.49, 6373.63)	7.49 (-1.39, 17.64)
Libya	LBY	2020	No Data	UMIC	EMR	1459.00	1863.44 (-384.61, 4345.61)	6.47 (-1.33, 15.14)
Libya	LBY	2021	No Data	UMIC	EMR	4237.00	5997.98 (3043.93, 8867.9)	20.37 (10.32, 30.22)
Lithuania	LTU	2020	Full National	HIC	EUR	1800.00	6651.48 (6140.17, 7196.99)	15.98 (14.56, 17.51)
Lithuania	LTU	2021	Full National	HIC	EUR	5597.00	10603.22 (10020.67, 11188.55)	25.48 (23.74, 27.26)
Luxembourg	LUX	2020	Full National	HIC	EUR	489.00	151.89 (73.56, 227.29)	3.42 (1.62, 5.19)
Luxembourg	LUX	2021	Full National	HIC	EUR	426.00	-82.41 (-180.04, 6.06)	-1.8 (-3.87, 0.14)
Madagascar	MDG	2020	No Data	LIC	AFR	261.00	7974.15 (-5885.11, 23642.59)	4.43 (-3.28, 13.14)
Madagascar	MDG	2021	No Data	LIC	AFR	766.00	17608.04 (2599.8, 33469.52)	9.67 (1.43, 18.41)
Malawi	MWI	2020	No Data	LIC	AFR	189.00	3089.82 (-4847.65, 11593.18)	2.89 (-4.53, 10.85)
Malawi	MWI	2021	No Data	LIC	AFR	2166.00	14023.33 (5646.93, 23360.32)	13.26 (5.33, 22.17)
Malaysia	MYS	2020	Full National	UMIC	WPR	463.00	-15091.04 (-16534.51, -13653.53)	-7.85 (-8.54, -7.16)
Malaysia	MYS	2021	Partial National	UMIC	WPR	30999.00	22624.01 (12799.32, 33140.8)	11.27 (6.35, 16.53)
Maldives	MDV	2020	Full National	UMIC	SEAR	48.00	2.99 (-30.19, 34.22)	0.24 (-2.17, 2.59)
Maldives	MDV	2021	Partial National	UMIC	SEAR	214.00	242.82 (95.69, 413.81)	17.81 (6.97, 30.27)
Mali	MLI	2020	No Data	LIC	AFR	269.00	12468.21 (-482.17, 26218.17)	8.08 (-0.31, 17)
Mali	MLI	2021	No Data	LIC	AFR	389.00	14842.72 (2339.14, 28123.86)	9.65 (1.52, 18.28)
Malta	MLT	2020	Full National	HIC	EUR	223.00	263.33 (184.42, 349.43)	6.76 (4.64, 9.16)
Malta	MLT	2021	Full National	HIC	EUR	253.00	213.34 (113.96, 309.32)	5.35 (2.78, 7.93)
Marshall Islands	MHL	2020	No Data	UMIC	WPR	0.00	-42.68 (-88.26, 7.44)	-10.18 (-21, 1.79)
Marshall Islands	MHL	2021	No Data	UMIC	WPR	0.00	-36.72 (-83.67, 14.7)	-8.67 (-19.69, 3.53)
Mauritania	MRT	2020	No Data	LMIC	AFR	324.00	2992.28 (766.15, 5455.85)	11.31 (2.91, 20.64)
Mauritania	MRT	2021	No Data	LMIC	AFR	539.00	3955.45 (1536.67, 6419.18)	14.84 (5.76, 24.13)
Mauritius	MUS	2020	Full National	UMIC	AFR	10.00	-471.75 (-626.58, -316.44)	-4.01 (-5.27, -2.73)
Mauritius	MUS	2021	Full National	UMIC	AFR	776.00	1413.56 (1233.93, 1591.44)	11.68 (10.04, 13.34)
Mexico	MEX	2020	Full National	UMIC	AMR	147623.00	314540.27 (305939.71, 323110.29)	41.9 (40.29, 43.53)
Mexico	MEX	2021	Full National	UMIC	AMR	155119.00	311676.27 (300349.47, 323208.86)	40.64 (38.58, 42.78)
Micronesia (Federated States of)	FSM	2020	No Data	LMIC	WPR	0.00	-62.86 (-158.85, 44.41)	-6 (-15.07, 4.25)
Micronesia (Federated States of)	FSM	2021	No Data	LMIC	WPR	0.00	-47.86 (-151.38, 58.27)	-4.45 (-14.06, 5.48)
Monaco	MCO	2020	Full National	HIC	EUR	3.00	10.22 (-14.71, 34.33)	1.99 (-2.65, 6.77)
Monaco	MCO	2021	Full National	HIC	EUR	35.00	53.64 (12.25, 91.61)	10.1 (2.11, 18.27)

Supplementary Table 9: Total reported COVID-19 and excess mortality measures for years 2020 and 2021. The measures shown are excess deaths and P-Scores by country and year. Country-specific information shown are the data availability type, World Bank income grouping and the WHO region.

Country	ISO3	Year	Data Type	Income Group	WHO Region	Reported COVID	Excess	P-Score
Mongolia	MNG	2020	Full National	LMIC	WPR	1.00	-2383.39 (-2640.13, -2145.31)	-9.92 (-10.87, -9.02)
Mongolia	MNG	2021	Full National	LMIC	WPR	1985.00	2387.4 (2072.7, 2721.93)	9.67 (8.29, 11.17)
Montenegro	MNE	2020	Full National	UMIC	EUR	677.00	870.37 (706.07, 1038.34)	12.04 (9.54, 14.68)
Montenegro	MNE	2021	Partial National	UMIC	EUR	1724.00	3039.44 (2573.38, 3583.38)	42.43 (34.95, 50.85)
Morocco	MAR	2020	No Data	LMIC	EMR	7355.00	15113.87 (-5979.21, 36558.14)	5.98 (-2.36, 14.45)
Morocco	MAR	2021	No Data	LMIC	EMR	7489.00	19634.56 (-1460.4, 40976)	7.6 (-0.57, 15.88)
Mozambique	MOZ	2020	No Data	LIC	AFR	163.00	5716.6 (-13900.66, 28061.23)	2 (-4.86, 9.85)
Mozambique	MOZ	2021	No Data	LIC	AFR	1813.00	37014.2 (11649.69, 62000.13)	12.9 (4.07, 21.55)
Myanmar	MMR	2020	No Data	LMIC	SEAR	2682.00	749.63 (-26848.68, 30586.77)	0.21 (-7.1, 8.18)
Myanmar	MMR	2021	No Data	LMIC	SEAR	16586.00	43438.63 (9401.11, 79888.41)	11.76 (2.57, 21.69)
Namibia	NAM	2020	No Data	UMIC	AFR	196.00	895.94 (-463.3, 2354.71)	4.98 (-2.57, 13.09)
Namibia	NAM	2021	No Data	UMIC	AFR	3420.00	6856.91 (5058.64, 8887.6)	38.63 (28.43, 50.17)
Nauru	NRU	2020	No Data	HIC	WPR	0.00	-0.78 (-15.99, 16.51)	-1.21 (-24.7, 25.91)
Nauru	NRU	2021	No Data	HIC	WPR	0.00	-0.68 (-16.75, 17.06)	-1.04 (-26.81, 26.76)
Nepal	NPL	2020	No Data	LMIC	SEAR	2758.00	7972.4 (-4480.73, 21756.07)	4.49 (-2.53, 12.32)
Nepal	NPL	2021	No Data	LMIC	SEAR	8836.00	24540.18 (9076.69, 41099.66)	13.69 (5.07, 22.86)
Netherlands	NLD	2020	Full National	HIC	EUR	11298.00	14481.44 (12588.56, 16225.5)	9.29 (7.97, 10.52)
Netherlands	NLD	2021	Full National	HIC	EUR	9589.00	14730.51 (12482.35, 16892.17)	9.35 (7.81, 10.86)
New Zealand	NZL	2020	Full National	HIC	WPR	25.00	-2064.7 (-2410.68, -1733.43)	-5.93 (-6.86, -5.03)
New Zealand	NZL	2021	Full National	HIC	WPR	26.00	-612.44 (-982.5, -252.63)	-1.72 (-2.74, -0.72)
Nicaragua	NIC	2020	Partial National	LMIC	AMR	165.00	9166.43 (7487.92, 11164.66)	27.72 (22.62, 33.75)
Nicaragua	NIC	2021	No Data	LMIC	AMR	52.00	2928.99 (-509.46, 6591.29)	8.67 (-1.5, 19.62)
Niger	NER	2020	No Data	LIC	AFR	96.00	15938.32 (272.41, 31332.38)	8.64 (0.15, 17)
Niger	NER	2021	No Data	LIC	AFR	178.00	18400.3 (3635.29, 34390.27)	9.92 (1.96, 18.49)
Nigeria	NGA	2020	No Data	LMIC	AFR	1278.00	60583.71 (-76897.74, 192137.2)	3.68 (-4.67, 11.67)
Nigeria	NGA	2021	No Data	LMIC	AFR	1752.00	125849.84 (-13147.14, 270017.46)	7.66 (-0.8, 16.43)
Niue	NIU	2020	No Data	UMIC	WPR	0.00	-2.5 (-9.76, 5.72)	-13.77 (-54.75, 32.23)
Niue	NIU	2021	No Data	UMIC	WPR	0.00	-2.47 (-10.11, 5.5)	-13.65 (-55.82, 30.55)
North Macedonia	MKD	2020	Full National	UMIC	EUR	2488.00	5712.65 (5248.25, 6144.54)	22.46 (20.25, 24.56)
North Macedonia	MKD	2021	Full National	UMIC	EUR	5478.00	9644.75 (9108.37, 10164.51)	37.55 (34.72, 40.37)
Norway	NOR	2020	Full National	HIC	EUR	433.00	-594.62 (-1001.59, -231.48)	-1.43 (-2.39, -0.56)
Norway	NOR	2021	Full National	HIC	EUR	947.00	493.46 (24.07, 953.47)	1.19 (0.06, 2.32)
Oman	OMN	2020	Full National	HIC	EMR	1497.00	4115.06 (3799.86, 4426.18)	25.18 (22.8, 27.6)
Oman	OMN	2021	Full National	HIC	EMR	2619.00	7384.46 (6955.82, 7803.83)	44.97 (41.26, 48.74)
Pakistan	PAK	2020	No Data	LMIC	EMR	10047.00	93543.71 (-36046.87, 231765.41)	5.67 (-2.19, 14.06)
Pakistan	PAK	2021	No Data	LMIC	EMR	18874.00	136896.57 (7012.94, 277767.64)	8.24 (0.42, 16.7)
Palau	PLW	2020	No Data	HIC	WPR	0.00	-19.66 (-49.61, 12.42)	-9.72 (-24.22, 6.15)
Palau	PLW	2021	No Data	HIC	WPR	0.00	-20.73 (-52.64, 10.72)	-10.14 (-25.59, 5.32)
Panama	PAN	2020	Full National	UMIC	AMR	3933.00	2997.46 (2774.92, 3209.76)	14.36 (13.15, 15.53)
Panama	PAN	2021	Partial National	UMIC	AMR	3492.00	4645.13 (3897.5, 5462.5)	21.7 (18.13, 25.62)
Papua New Guinea	PNG	2020	No Data	LMIC	WPR	9.00	-1810.06 (-6275.78, 2769.24)	-2.95 (-10.19, 4.52)
Papua New Guinea	PNG	2021	No Data	LMIC	WPR	581.00	2571.22 (-1923.85, 7566.79)	4.14 (-3.09, 12.18)
Paraguay	PRY	2020	Full National	UMIC	AMR	2220.00	2410.48 (1844.79, 2996.87)	6.9 (5.19, 8.72)
Paraguay	PRY	2021	Full National	UMIC	AMR	14404.00	17406.87 (16612.67, 18190.88)	49.04 (45.76, 52.38)
Peru	PER	2020	Full National	UMIC	AMR	93066.00	133816.37 (131320.83, 136189.07)	91.11 (87.91, 94.24)
Peru	PER	2021	Full National	UMIC	AMR	109518.00	155851.18 (152540.19, 159146.2)	102.84 (98.48, 107.32)

Supplementary Table 10: Total reported COVID-19 and excess mortality measures for years 2020 and 2021. The measures shown are excess deaths and P-Scores by country and year. Country-specific information shown are the data availability type, World Bank income grouping and the WHO region.

Country	ISO3	Year	Data Type	Income Group	WHO Region	Reported COVID	Excess	P-Score
Philippines	PHL	2020	Full National	LMIC	WPR	9230.00	-40147.49 (-45475.36, -35073.08)	-5.24 (-5.89, -4.61)
Philippines	PHL	2021	Partial National	LMIC	WPR	42143.00	225400.29 (205554.99, 247793.8)	28.22 (25.64, 31.08)
Poland	POL	2020	Full National	HIC	EUR	28641.00	60686.98 (56012.46, 65634.01)	14.53 (13.26, 15.89)
Poland	POL	2021	Full National	HIC	EUR	68416.00	96843.54 (91196.64, 102531.33)	22.9 (21.27, 24.57)
Portugal	PRT	2020	Full National	HIC	EUR	6830.00	9907.73 (7680.05, 12078.38)	8.7 (6.61, 10.8)
Portugal	PRT	2021	Full National	HIC	EUR	12107.00	10539.5 (8229.12, 12882.69)	9.21 (7.04, 11.47)
Qatar	QAT	2020	Full National	HIC	EMR	245.00	716.69 (655.4, 779.56)	15.25 (13.76, 16.81)
Qatar	QAT	2021	Full National	HIC	EMR	372.00	822.4 (745.5, 894.5)	16.8 (14.99, 18.54)
Republic of Korea	KOR	2020	Full National	HIC	WPR	900.00	248.44 (-3049.12, 3393.26)	0.08 (-0.99, 1.12)
Republic of Korea	KOR	2021	Full National	HIC	WPR	4663.00	6039.57 (2148.86, 9782.18)	1.94 (0.68, 3.17)
Republic of Moldova	MDA	2020	Full National	UMIC	EUR	3110.00	5067.8 (4294.97, 5887.72)	12.65 (10.51, 14.99)
Republic of Moldova	MDA	2021	Full National	UMIC	EUR	7159.00	13089.77 (12107.29, 13994.02)	33.31 (30.04, 36.43)
Romania	ROU	2020	Full National	UMIC	EUR	15596.00	34995.92 (31447.1, 38424.38)	13.36 (11.84, 14.85)
Romania	ROU	2021	Full National	UMIC	EUR	43118.00	71915.86 (67747.97, 76142.17)	27.42 (25.42, 29.5)
Russian Federation	RUS	2020	Full National	UMIC	EUR	57019.00	369306.43 (351737.33, 386423.72)	20.88 (19.68, 22.05)
Russian Federation	RUS	2021	Full National	UMIC	EUR	251841.00	703019.46 (683875.54, 723962.49)	40.35 (38.82, 42.05)
Rwanda	RWA	2020	No Data	LIC	AFR	86.00	-369.62 (-5205.31, 4626.54)	-0.57 (-8.02, 7.14)
Rwanda	RWA	2021	No Data	LIC	AFR	1263.00	5770.74 (403.34, 11665.73)	8.8 (0.62, 17.75)
Saint Kitts and Nevis	KNA	2020	Annual Data	HIC	AMR	0.00	-131.02 (-151.83, -109.77)	-25.71 (-29.98, -21.57)
Saint Kitts and Nevis	KNA	2021	No Data	HIC	AMR	28.00	-76.35 (-246.14, 158)	-14.34 (-45.97, 29.8)
Saint Lucia	LCA	2020	No Data	UMIC	AMR	5.00	-109.99 (-254.5, 27.67)	-7.03 (-16.25, 1.77)
Saint Lucia	LCA	2021	No Data	UMIC	AMR	290.00	479.91 (291.69, 668.3)	29.63 (17.96, 41.54)
Saint Vincent and the Grenadines	VCT	2020	Annual Data	UMIC	AMR	0.00	106.02 (88.33, 123.03)	11.29 (9.42, 13.17)
Saint Vincent and the Grenadines	VCT	2021	No Data	UMIC	AMR	83.00	386.55 (53.79, 796.03)	41.01 (5.64, 85.61)
Samoa	WSM	2020	No Data	LMIC	WPR	0.00	-56.29 (-168.87, 59.99)	-4.68 (-14.05, 5.01)
Samoa	WSM	2021	No Data	LMIC	WPR	0.00	-32.31 (-138.83, 89.38)	-2.67 (-11.52, 7.39)
San Marino	SMR	2020	Full National	HIC	EUR	59.00	110.48 (97.83, 122.86)	36.7 (31.14, 42.49)
San Marino	SMR	2021	Full National	HIC	EUR	40.00	60.05 (44.16, 76.03)	19.71 (13.72, 26.22)
Sao Tome and Principe	STP	2020	No Data	LMIC	AFR	17.00	70.78 (-31.4, 171.78)	7.12 (-3.15, 17.47)
Sao Tome and Principe	STP	2021	No Data	LMIC	AFR	40.00	120.11 (12.79, 234.54)	12.06 (1.29, 23.5)
Saudi Arabia	SAU	2020	No Data	HIC	EMR	6214.00	12625.98 (-190.48, 25079.54)	8.66 (-0.13, 17.2)
Saudi Arabia	SAU	2021	No Data	HIC	EMR	2661.00	4923.67 (-7085.95, 17039.11)	3.32 (-4.77, 11.54)
Senegal	SEN	2020	No Data	LMIC	AFR	402.00	4845.18 (-1499.04, 11886.21)	5.6 (-1.73, 13.74)
Senegal	SEN	2021	No Data	LMIC	AFR	1488.00	11658.91 (4436.75, 19045.9)	13.48 (5.14, 22)
Serbia	SRB	2020	Full National	UMIC	EUR	3163.00	17171.87 (15215.57, 19100.31)	14.8 (12.89, 16.73)
Serbia	SRB	2021	Full National	UMIC	EUR	9525.00	38471.61 (36372.09, 40720.84)	33.11 (30.74, 35.73)
Seychelles	SYC	2020	Full National	HIC	AFR	0.00	-172.83 (-193.19, -152.62)	-20.54 (-22.43, -18.6)
Seychelles	SYC	2021	Partial National	HIC	AFR	126.00	179.76 (57.78, 313.58)	20.75 (6.59, 36.47)
Sierra Leone	SLE	2020	No Data	LIC	AFR	76.00	3566.21 (-1667.76, 9353.13)	5.45 (-2.57, 14.35)
Sierra Leone	SLE	2021	No Data	LIC	AFR	47.00	4318.55 (-1047.87, 10276.34)	6.75 (-1.64, 16.1)
Singapore	SGP	2020	Full National	HIC	WPR	29.00	-30.47 (-312.04, 253.68)	-0.11 (-1.11, 0.92)
Singapore	SGP	2021	Full National	HIC	WPR	798.00	1505.35 (1129.25, 1905.68)	5.2 (3.85, 6.67)
Slovakia	SVK	2020	Full National	HIC	EUR	2138.00	5462.14 (4720.41, 6202.56)	10.17 (8.66, 11.7)
Slovakia	SVK	2021	Full National	HIC	EUR	14497.00	18858.02 (17998.19, 19700.58)	35.04 (32.91, 37.18)

Supplementary Table 11: Total reported COVID-19 and excess mortality measures for years 2020 and 2021. The measures shown are excess deaths and P-Scores by country and year. Country-specific information shown are the data availability type, World Bank income grouping and the WHO region.

Country	ISO3	Year	Data Type	Income Group	WHO Region	Reported COVID	Excess	P-Score
Slovenia	SVN	2020	Full National	HIC	EUR	2955.00	3345.58 (2975.31, 3704.56)	16.02 (13.99, 18.03)
Slovenia	SVN	2021	Full National	HIC	EUR	3164.00	2236.26 (1732.81, 2748.4)	10.69 (8.07, 13.44)
Solomon Islands	SLB	2020	No Data	LMIC	WPR	0.00	-69.65 (-468.59, 318.82)	-1.43 (-9.63, 6.55)
Solomon Islands	SLB	2021	No Data	LMIC	WPR	0.00	9.52 (-397.69, 425.77)	0.19 (-8, 8.51)
Somalia	SOM	2020	No Data	LIC	EMR	130.00	12918.3 (-608.18, 28514.7)	7.64 (-0.36, 16.82)
Somalia	SOM	2021	No Data	LIC	EMR	1203.00	22542.22 (7909.15, 37419.68)	13.39 (4.69, 22.27)
South Africa	ZAF	2020	Full National	UMIC	AFR	28033.00	54402.02 (49157.12, 59474.6)	10.32 (9.23, 11.39)
South Africa	ZAF	2021	Full National	UMIC	AFR	63028.00	184268.99 (178559.45, 189658.68)	35.01 (33.56, 36.41)
South Sudan	SSD	2020	No Data	LIC	AFR	63.00	2798.28 (-4321.27, 9817.16)	3.1 (-4.77, 10.86)
South Sudan	SSD	2021	No Data	LIC	AFR	72.00	6367.88 (-596.27, 14129.48)	7.1 (-0.66, 15.69)
Spain	ESP	2020	Full National	HIC	EUR	54055.00	72576.06 (66238.31, 78989.73)	16.74 (15.05, 18.48)
Spain	ESP	2021	Full National	HIC	EUR	36950.00	31360.92 (24285.96, 39166.07)	7.19 (5.47, 9.13)
Sri Lanka	LKA	2020	Annual Data	LMIC	SEAR	204.00	-17357.78 (-20469.59, -14027.95)	-11.58 (-13.39, -9.58)
Sri Lanka	LKA	2021	Partial National	LMIC	SEAR	14775.00	8526.6 (-5501.75, 23659.41)	5.58 (-3.57, 15.54)
Sudan	SDN	2020	No Data	LIC	EMR	1561.00	14225.09 (-5832.9, 34610.01)	5.86 (-2.41, 14.3)
Sudan	SDN	2021	No Data	LIC	EMR	1776.00	23773.55 (2989.42, 45311.95)	9.73 (1.22, 18.53)
Suriname	SUR	2020	Full National	UMIC	AMR	120.00	-286.72 (-340.9, -231.11)	-6.01 (-7.06, -4.9)
Suriname	SUR	2021	Partial National	UMIC	AMR	1069.00	1013.26 (858.72, 1189.74)	20.07 (16.84, 23.79)
Sweden	SWE	2020	Full National	HIC	EUR	9650.00	10469.41 (6490.81, 14378.41)	11.74 (10.35, 13.22)
Sweden	SWE	2021	Full National	HIC	EUR	5715.00	2969.29 (-1614.46, 7518.91)	7.56 (6.50, 8.63)
Switzerland	CHE	2020	Full National	HIC	EUR	7517.00	7540.16 (6685.65, 8440.89)	10.77 (9.43, 12.2)
Switzerland	CHE	2021	Full National	HIC	EUR	4385.00	707.06 (-310.05, 1801.14)	1 (-0.43, 2.58)
Syrian Arab Republic	SYR	2020	No Data	LIC	EMR	704.00	1634.52 (-7106.04, 10812.99)	2.38 (-9.34, 15.42)
Syrian Arab Republic	SYR	2021	No Data	LIC	EMR	2189.00	5679.32 (-3966.31, 15998.76)	9.63 (-5.98, 28.06)
Tajikistan	TJK	2020	Full National	LMIC	EUR	90.00	13555.47 (12489.61, 14598.16)	27.14 (24.47, 29.83)
Tajikistan	TJK	2021	No Data	LMIC	EUR	35.00	-546.74 (-5855.64, 4957.54)	-1.08 (-11.68, 10.02)
Thailand	THA	2020	Full National	UMIC	SEAR	61.00	-15606.42 (-23521.41, -7760.6)	-3.01 (-4.48, -1.52)
Thailand	THA	2021	Full National	UMIC	SEAR	21637.00	30907.7 (18296.46, 43275.38)	5.82 (3.35, 8.32)
The United Kingdom	GBR	2020	Full National	HIC	EUR	75112.00	85504.32 (76126.93, 94916.21)	13.84 (12.13, 15.6)
The United Kingdom	GBR	2021	Full National	HIC	EUR	74492.00	63392.44 (53118.83, 74604.74)	10.24 (8.43, 12.26)
Timor-Leste	TLS	2020	No Data	LMIC	SEAR	0.00	43.48 (-545.69, 703.65)	0.57 (-7.09, 9.11)
Timor-Leste	TLS	2021	No Data	LMIC	SEAR	122.00	639.58 (-64.43, 1366.74)	8.15 (-0.82, 17.35)
Togo	TGO	2020	Full National	LIC	AFR	68.00	-5217.91 (-5435.38, -4975.74)	-9.54 (-9.9, -9.14)
Togo	TGO	2021	Full National	LIC	AFR	180.00	-1770.84 (-2079.43, -1466.35)	-3.26 (-3.81, -2.72)
Tonga	TON	2020	No Data	UMIC	WPR	0.00	-23.21 (-89.48, 45.08)	-3.69 (-14.19, 7.18)
Tonga	TON	2021	No Data	UMIC	WPR	0.00	-13.5 (-78.43, 59.19)	-2.14 (-12.42, 9.43)
Trinidad and Tobago	TTO	2020	No Data	HIC	AMR	126.00	92.05 (-739.38, 913.39)	0.98 (-7.82, 9.77)
Trinidad and Tobago	TTO	2021	No Data	HIC	AMR	2699.00	1938.91 (950.86, 2958.15)	20.83 (10.25, 31.81)
Tunisia	TUN	2020	Full National	LMIC	EMR	4620.00	3142.68 (1942.18, 4352.42)	4.38 (2.66, 6.16)
Tunisia	TUN	2021	Partial National	LMIC	EMR	20944.00	20678.8 (12536.24, 29053.06)	28.09 (16.97, 39.86)
Turkey	TUR	2020	Subnational Data	UMIC	EUR	20642.00	126390.92 (108577.62, 145361.39)	28.82 (24.8, 33.13)
Turkey	TUR	2021	Subnational Data	UMIC	EUR	61556.00	137650.44 (119504.12, 158698.31)	30.87 (26.79, 35.72)
Turkmenistan	TKM	2020	No Data	UMIC	EUR	0.00	785.35 (-2129.09, 3816.04)	2.11 (-5.72, 10.27)
Turkmenistan	TKM	2021	No Data	UMIC	EUR	0.00	-133.68 (-3033.46, 3257.36)	-0.34 (-7.94, 8.69)
Tuvalu	TUV	2020	No Data	UMIC	WPR	0.00	-5.95 (-25.04, 14.63)	-5.66 (-23.84, 14.02)
Tuvalu	TUV	2021	No Data	UMIC	WPR	0.00	-5 (-25.76, 17.61)	-4.74 (-24.35, 16.56)

Supplementary Table 12: Total reported COVID-19 and excess mortality measures for years 2020 and 2021. The measures shown are excess deaths and P-Scores by country and year. Country-specific information shown are the data availability type, World Bank income grouping and the WHO region.

**Country**	**ISO3**	**Year**	**Data Type**	**Income Group**	**WHO Region**	**Reported COVID**	**Excess**	**P-Score**
Uganda	UGA	2020	No Data	LIC	AFR	303.00	2828.57 (-13677.34, 20183.14)	1.25 (-6.01, 8.88)
Uganda	UGA	2021	No Data	LIC	AFR	2992.00	17689.96 (-959.31, 37556.46)	7.81 (-0.42, 16.55)
Ukraine	UKR	2020	Full National	LMIC	EUR	18533.00	45366.77 (36995.59, 53463.73)	7.75 (6.23, 9.26)
Ukraine	UKR	2021	Full National	LMIC	EUR	77366.00	152859.7 (143822.04, 162128.85)	26.47 (24.51, 28.53)
United Arab Emirates	ARE	2020	No Data	HIC	EMR	665.00	928.72 (-728.58, 2735.6)	4.23 (-3.32, 12.48)
United Arab Emirates	ARE	2021	No Data	HIC	EMR	1497.00	1401.17 (-567.78, 3457.1)	6.08 (-2.46, 15)
United Republic of Tanzania	TZA	2020	No Data	LMIC	AFR	21.00	14727.71 (-10679.76, 39813.73)	4.59 (-3.32, 12.41)
United Republic of Tanzania	TZA	2021	No Data	LMIC	AFR	716.00	25301.95 (-4518.67, 54287.49)	7.91 (-1.41, 16.97)
United States of America	USA	2020	Full National	HIC	AMR	351839.00	465706 (436048.56, 493570.08)	15.33 (14.21, 16.39)
United States of America	USA	2021	Full National	HIC	AMR	466624.00	466752.44 (430260.92, 502453.95)	14.98 (13.64, 16.31)
Uruguay	URY	2020	Full National	HIC	AMR	168.00	-2245.55 (-2694.93, -1784.22)	-6.43 (-7.63, -5.18)
Uruguay	URY	2021	Full National	HIC	AMR	6000.00	5351.6 (4788.61, 5908.7)	15.19 (13.37, 17.03)
Uzbekistan	UZB	2020	Full National	LMIC	EUR	614.00	22812.71 (17784.46, 27727.44)	13.39 (10.12, 16.73)
Uzbekistan	UZB	2021	Full National	LMIC	EUR	871.00	22073.59 (15875.45, 28337.3)	13.01 (9, 17.28)
Vanuatu	VUT	2020	No Data	LMIC	WPR	0.00	-72.45 (-264.98, 125.05)	-3.28 (-12.05, 5.67)
Vanuatu	VUT	2021	No Data	LMIC	WPR	0.00	-3.91 (-196.34, 201.47)	-0.17 (-8.68, 8.91)
Venezuela (Bolivarian Republic of)	VEN	2020	No Data	HIC	AMR	1021.00	3641.51 (-11403.29, 19076.75)	2 (-6.29, 10.47)
Venezuela (Bolivarian Republic of)	VEN	2021	No Data	HIC	AMR	4300.00	18683.91 (3785.56, 33942.59)	10.01 (2.04, 18.17)
Viet Nam	VNM	2020	Annual Data	LMIC	WPR	35.00	-58679.26 (-59601.85, -57772.27)	-7.96 (-8.08, -7.83)
Viet Nam	VNM	2021	No Data	LMIC	WPR	32133.00	52468.26 (-135388.08, 281578.14)	7.02 (-18.11, 37.68)
Yemen	YEM	2020	No Data	LIC	EMR	611.00	14407.76 (33.81, 30836.36)	8.06 (0.02, 17.27)
Yemen	YEM	2021	No Data	LIC	EMR	1373.00	19384.56 (3552.01, 35760.29)	10.57 (1.95, 19.49)
Zambia	ZMB	2020	No Data	LMIC	AFR	386.00	5293.68 (-4225.41, 15074.03)	4.36 (-3.48, 12.39)
Zambia	ZMB	2021	No Data	LMIC	AFR	3344.00	18259.13 (7382.07, 28834.85)	15.04 (6.06, 23.77)
Zimbabwe	ZWE	2020	No Data	LMIC	AFR	361.00	1475.69 (-6905.35, 10642.39)	1.27 (-5.96, 9.21)
Zimbabwe	ZWE	2021	No Data	LMIC	AFR	4637.00	17155.99 (7983.81, 27426.53)	15 (6.98, 23.98)

Supplementary Table 13: Total reported COVID-19 and excess mortality measures for years 2020 and 2021. The measures shown are excess deaths and P-Scores by country and year. Country-specific information shown are the data availability type, World Bank income grouping and the WHO region.

References

Anand, A., Sandefur, J., Subramanian, A., et al. (2021). *Three new estimates of India's all-cause excess mortality during the COVID-19 pandemic*. Center for Global Development.

Boyd, C., Ely, J., and Craig, E. (2022). Britain has LOWER Covid excess death rate than Spain, Germany and Italy - despite doom-mongers insisting UK fared worst in Europe. *The Daily Mail*. 5th May, 2022.

House of Commons (2021). Coronavirus: lessons learned to date. `https://committees.parliament.uk/publications/7496/documents/78687/default/`. Sixth Report of the Health and Social Care Committee and Third Report of the Science and Technology Committee of Session 2021-22.

Jha, P., Deshmukh, Y., Tumbe, C., Suraweera, W., Bhowmick, A., Sharma, S., Novosad, P., Fu, S. H., Newcombe, L., Gelband, H., et al. (2022). COVID mortality in India: National survey data and health facility deaths. *Science*, page eabm5154.

Knutson, V., Aleshin-Guendel, S., Karlinsky, A., Msemburi, W., and Wakefield, J. (2023). Estimating country-specific excess mortality during the COVID-19 pandemic. *Annals of Applied Statistics (In Press)*. https://arxiv.org/abs/2205.09081.

Reuters (2020). UK death toll 27,241, Labour leader Starmer says. `https://www.reuters.com/article/uk-health-coronavirus-britain-starmer/uk-death-toll-27241-labour-leader-starmer-says-idUKKBN22B1K1`.

Spiegelhalter, D. (2020). Coronavirus deaths: how does Britain compare with other countries? https://www.theguardian.com/commentisfree/2020/apr/30/coronavirus-deaths-how-does-britain-compare-with-other-countries.
