# Supplementary material for: The WHO estimates of excess mortality associated with the COVID-19 pandemic
Source: Nature. 2022 Dec 14;613(7942):130–7. doi: 10.1038/s41586-022-05522-2 (PMC9812776; doi:10.1038/s41586-022-05522-2)
Supplement: Supplementary file 3 — Peer Review File [file 41586_2022_5522_MOESM3_ESM.pdf]

Peer Review File

Manuscript Title: The WHO estimates of excess mortality associated with the COVID-19 pandemic

Reviewer Comments & Author Rebuttals

Reviewer Reports on the Initial Version:

Referees' comments:

Referee #1 (Remarks to the Author):

Proper ascertainment, via state-of-the-art estimation methods, of excess mortality for the pandemic years, at this time 2020 and 2021, is of the utmost important to gauge the impact of the pandemic on a very important parameter. This paper is not the first to address this important problem but, in my view, has the potential to be the most successful of all to date. Well-known attempts are that of IHME and The Economist. The authors voice justified criticisms against these two approaches and then try to overcome them in their own approach. These are:

- Models that are arguably too simplistic, in view of the large heterogeneity in data availability and quality – this applies predominantly to IHME; in my view, to the point that the IHME approach should be discarded.
- Models that do take into account national and regional differences, but are not sufficiently methodologically principled, perhaps even black box – this applies to The Economist. While this criticism is justified, the estimates from The Economist are widely considered plausible.

My main concern with the paper as it stands now is a certain lack of organization. Even though addressing it might require considerable editing work, it is not a methodological criticism. Slightly overstating the issue, the Nature submission might come across as 'a less technical version of the companion paper'. I will expand on this and offer some suggestions for redistribution and reorganization of the material.

Related to this, the Nature submission should have a more crisp structure:
- Background: Problem statement.
- Everything to do with data and the problems with the data: this involves the current Process and Data sections. Regarding data availability, I would like to see a clear statement regarding the issues that the modeler is confronted with. Ideally, data are of high quality, available quickly and without interruption, nationally and regionally, and at sufficiently short time intervals (e.g., monthly). Then, everything that deviates from this is a problem that the modeler needs to overcome, with clever, appropriate methods, and inevitable with plausible but unverifiable assumptions. I think that the authors do a very good job in this regard, but I am not sure the Nature reader will easily appreciate this. This brings me to…
- Methodology: in the main Nature paper, there is a bit too much statistical jargon (even though most statisticians would disagree with this perhaps). The methodology should be presented continually with an eye on how the above issues are overcome. I would be inclined not to present formulas in the main paper, but describe how various modeling tools are used to work with 'sub-optimal data' to satisfaction. The formula-based pieces could be assembled in the Nature

manuscript's appendix/supplement.
- Related to the above, I would suggest to explain some of the issues that occur based on a set of countries that are now discussed in the companion paper. What is now in the companion paper on page 21 on Germany and Sweden, I would tend to move to the main Nature paper. Likewise, the Indian case is extremely important, from a methodological perspective (can be elaborated form that angle in the companion) and from a mortality perspective (should go in the Nature submission).
- When presenting methods, the Nature submission should spell out which covariates and auxiliary information are used. This should all come at once, and not dotted around the paper(s). For example, as a reader I would like to know quickly whether reported COVID-19 mortality is used, whether seasonality/temperature is used, whether confirmed cases, hospitalizations, ICU occupancy,… are used, etc. Likewise, the age and gender structuring should be discussed. With all of these, brief motivations as to why or why not these are used, would be extremely useful.
- The main focus should be on the results and interpretation thereof. This will connect seamlessly to the discussion of some specific countries in the previous item.
- The results should be contrasted with those by other efforts (IHME, The Economist, but also others). Especially for The Economist, it would be good to move beyond the mere (and justified) comment that the method is not sufficiently theoretically backed up. In other words, "it seems to work" and a case in point is that the ratio of 2.75 in the current work is not too far away from the about 3.0 to 3.3 that The Economist has been obtaining. I realize that such a comparison is given in the companion paper, but I suggest to discuss it (predominantly) in the Nature submission. That actually strengthens the current paper and does not subtract from the fact that, in the long run, the current method might be more sustainable.
- Returning to India, some interesting developments are in the supplement of the companion paper. A broadly accessible narrative belongs, in my view, in the Nature submission – perhaps in its Supplement.
- The section on ranking is a bit a digression. At the same time, it is extremely important as every effort should be made to warn against over-interpretation of statistical estimates by properly taking uncertainty into account. This problem is not new, of course, and pops up all over empirical research (e.g., hospital performance). The problem has exacerbated during the pandemic, so it is very opportune to talk about it here. I would therefore make it a more prominent part of the paper, by referring to it briefly but clearly in the Abstract, Introductory sections, and Discussion. A disadvantage is that we do not get a simple, one-dimensional ordered list. This should be explained and it should be clear to the reader that it is simply unavoidable. The current statement about a 'two-dimensional summary/projection of a 6-D object' is likely not going to do the job.

Some further specific comments

1. The large table in Appendix B is immensely useful and I already look forward to the 2022 update. Unless I overlooked it, I was missing the United Kingdom. I would like to see a bit of discussion around negative estimates, i.e., undermortality. Of course, this might occur because of poor data availability – then usually accompanied by wide intervals. But there are some countries where this is the case because of policy. A good example is Norway, where the negative estimate remains even if we add 2020 and 2021. It is at start contract with Sweden where the combined estimate is 11,255; Denmark and Finland are in between with a total of 3000 to 4000. In Sweden, the toll is higher in 2020, whereas in the others it is the other way around. Given the endless debate about Sweden's

approach, this paper's modeling effort offers the authority to briefly discuss the Nordic countries. I left out Iceland, in spite of its two negative estimates, given the extremely small size of the country.

2. In terms of context, it might make sense to give some brief comparisons with historic sources of excess mortality. Of course, the further we go back in time, the less reliable the sources become, but we have, for example, influenza epidemics in the 1950 and 1960 (e.g., Hong Kong flu), World War II, and the Spanish flu.

3. Aron and Muellbauer (2020), referenced in the paper, provide early estimates (Spring 2020 wave) of underreporting of COVID-19 related mortality in a small set of countries. For countries like Spain and Belgium, the results in this paper for 2020 seem to be in line with their estimate (which, admittedly, does not address excess mortality but true COVID-19 deaths). For the Netherlands, even when accounting for a discrepancy between total COVID-19 deaths and excess mortality, this paper's estimate for 2020 seems to be a bit optimistic.

4. A very powerful message is that the excess mortality is higher in 2021 than in 2020. For this reason, having 2020 and 2021 columns in Table 1, in addition to the combined estimates, would be useful, pretty much as is the case in the Appendix table. Some comments as to the reasons for this would be welcome. We started vaccinating in 2021, but there was vaccine hesitancy, equity problems, waning, increasingly pathogenic variants (Alpha and Delta in particular in 2021, but some others in Latin America, such as Lambda and Mu), and less support in policy makers and general public for non-pharmaceutical interventions.

5. The message in the Disclaimer is important. In some countries, there has been pressure on researchers not to report or 'report optimistically' mortality. It will be comforting for the reader to know that the current work has been done without the influence of any such pressures. I have no reason to doubt this personally, though.

6. In Europe, EUROMOMO has been monitoring (excess) mortality in a number of countries or regions. Would this be a worthwhile source?

7. The authors indicate how they deal with aligning national and subnational data, when both are available. Has there been any adjudication in cases where there were blatant discrepancies, in case the problem occurred.

8. The authors correctly indicate that countries with good and poor reporting are not distributed uniformly around the globe. Still, would there be any hope to use geographical proximity (hence, spatial methodology) to borrow information from well-reporting countries in the neighborhood to inform those with data of lesser quality?

9. The section starting on page 8 is an illustration of my earlier point where methodological presentation should be re-thought. This is a point where the interested but less technical reader will be lost.
10. Page 9, penultimate paragraph: unresolved LaTeX reference

11. When stating that you have data on 17 Indian states, it would be good to state the total number as well.

12. Explain what you mean by ‘generative model’ if the term is going to be preserved.

13. The reader might be lost over ‘and with the models for different data types being consistent with each other’

14. The P-score, with its shortcomings, is a useful measure and happy to see it reported in the Appendix table. Explain it as non-technical as possible. The technical reader can easily access one of the technical references, or you could even explain it formula based in the Supplement.

15. Page 12, line -13: There is nothing wrong with the sentence starting with “This sharp increase…” yet many people might have to read it a few times…

16. Page 12, line -10: state --> states

17. Figure 7, make the legend more descriptive

18. Discussion, line 2: also here, give numbers and intervals for 2020 and 2021 separately. Of course, the interval for 2020+2021 will not simply be the sum of the other intervals, but the point estimates will.

19. Discussion, line 2: avoid ‘significant’ in this slightly non-technical sense

20. Here, the 2.74 should be contrasted with (at least) the corresponding The Economist estimate. This number alone, and the ensuing estimate of the total excess mortality for the years 2020 and 2021 should be estimated in an authoritative way and this paper has the potential to do it.

21. I like the careful discussion in the middle of page 22 on the attribution of excess deaths to COVID-19. Not everyone on the planet will be convinced, but it is a sound conclusion, based on proper research. One may also want to discuss harvesting: in some countries, with high mortality in 2020 and high quality reporting, one often sees a much lower excess mortality in 2021, and a bigger discrepancy between COVID-19 and excess deaths – against the dominant trend. This is the case for Sweden, Belgium, France,…

22. On page 23, second half, reference is made to mortality as part of a monitoring system. I agree with this if properly qualified. I think it is clear that this will not be a component of an early-warning system. For that, we need GP workload, wastewater surveillance, genomic surveillance, and general monitoring of emerging pathogens. It will play an important role when a health crisis is ongoing. For example, if we would have had this model in the middle of 2020 and later, it could have contributed to counterargue the ‘it has become a mere flu-like condition’ argument. At least until now, The Economist results have played this role to some extent.

23. In this regard, the shiny app is a wonderful tool.

Geert Molenberghs

Referee #2 (Remarks to the Author):

The paper presents estimates of excess mortality, directly and indirectly, attributable to COVID-19 during the years 2020 and 2021. It is a significant contribution to the analysis and assessment of the global mortality burden of the pandemic. There is a massive work behind these analyses in data collection, curation, modeling, and analysis of the estimates. In general, the approach, data quality, and presentation quality are valid, although there are a few suggestions to improve the modeling strategy. The statistics and treatment of uncertainties seem appropriate. The use of a Bayesian approach, integrating prior uncertainty, is convenient. The manuscript is very well written. The abstract, introduction, and conclusions are lucid and appropriate.

The authors take a considerable risk in estimating excess mortality in countries where information on all-cause mortality is unavailable in the years 2020 and 2021. Although this estimation implies substantial potential biases, the authors acknowledge these risks well and do the best possible with the available resources. In this sense, the exercise is essential and hopefully a good approximation to the worldwide mortality crisis caused by the pandemic. The call for improving monitoring systems, vital statistics, and civil registrations is very pertinent and much needed.

Major issues:

In general, I think the models are robust. However, I am concerned about the parameterization for fitting the baseline mortality. For fitting monthly mortality, the authors use a thin-plate spline for the "annual trend" and a cyclic cubic spline for the seasonable component. Splines are great for interpolation but generally very risky for extrapolation. Splines extrapolation only uses the last fitted coefficients, depending on the order of difference selected for fitting the spline. The authors do not give information regarding the order of difference parameterized for the spline fitting. However, according to the information provided in the methodological paper, the fitting was done using the default options of the mgcv package, which uses 2nd order difference for the splines. Under a 2nd order difference parameterization, the model linearly extrapolates based on the last two coefficients. This extrapolation has the risk of generating inadequate baselines in several cases. To avoid this, I would suggest using a safer option, such as a log-linear parameterization for the secular change in mortality. In the methodological paper, the authors mention this issue and note that adjustments of this kind were made to Germany and Sweden but not to the other populations. Those adjustments and the identified limitation should be acknowledged in this manuscript. It should be justified why the log-linear trend was exclusively applied to two countries and not to all of them. I can think of many reasons for using splines for interpolation, but none that justifies using splines for extrapolation. If the authors see the use of splines as an advantage over a simpler log-linear trend, it should be justified.

The selection of contextual variables for extrapolating the estimates to countries with no data seems adequate, as they take into consideration proxies for Covid-19 activity and severity (confirmed deaths, positivity rate, etc.), country-specific containment measures, and different indicators of population-specific vulnerability to COVID-19 (cardiovascular and diabetes prevalence, and income). Of course, it is always possible to include a large number of variables, as it is done in other modeling attempts (e.g., Economist). Still, I think the conservative approach of the authors is a wise decision. However, there seems to be a critical omission of one of the most important determinants of the

risk of death in a population in the context of COVID-19: information on the proportion of people at old ages in the population (Dowd et al., 2020; Goldstein & Lee, 2020; Sasson, 2021).

Other issues:

The authors propose a proportionality assumption for countries where data is restricted to subnational regions. According to this hypothesis, the subnational share of ACM is the same before and during the pandemic. However, there is no evaluation of how plausible is this assumption, or at least a mention of this. With the available data, it would be possible to evaluate this assumption in countries where complete monthly data is available at the national and subnational levels (e.g., the US). Such sensitivity analysis would inform the potential bias under the proportionality assumption.

If the excess is estimated annually and no attempts are made for influenza correction, it is unclear why it is essential to use monthly data for the baseline mortality estimation. Fitting annual data is more straightforward than monthly data, and the estimates are consistent (Nepomuceno et al., 2022). I suppose the importance of this temporal resolution could be related to the time-varying variables used for extrapolating the estimates to countries with no data (?). However, this is my guess, and I am not even convinced about it. I think there is a need for more clarity in this respect, as the use of monthly data makes the modeling considerably more complex and implies strong assumptions for the countries with no monthly data.

In addition, the model for partitioning annual death counts to monthly is poorly described. According to the methodological paper, the partition is done using a model including information on temperature. Therefore, it would be important to mention the use of temperature in this paper briefly. This mention is essential because the inclusion of temperature guarantees that the monthly partition follows the divergences in seasonality across countries.

The model assumes influenza mortality in 2020 and 2021 to be similar to the exceptionally severe seasonal epidemics in 2015-2019. The authors acknowledge that there is evidence that influenza circulation was exceptionally low during 2020 and 2021, which implies a considerable underestimation of excess mortality. Still, there is no attempt to adjust this bias. This wouldn't be problematic if there were no way to take into consideration the exceptional low influenza mortality, but there are several excess mortality models that would allow for this (Simonsen et al., 2006; Thompson et al., 2009) and correct for a bias that seems to be non-negligible in the context of the COVID-19 pandemic (Shkolnikov et al., 2022). The use of monthly data would allow for such adjustments.

Last but not least, it is excellent that the WHO publishes the mortality estimates by country in the shiny app. The authors state, "This tool allows transparent exploration of estimates from the country level up to the regional and global level." However, there is no access to the scripts that were employed for the estimation of excess mortality. The publication of the scripts to reproduce the analysis presented here would allow for a transparent exploration not only of the outputs but, more importantly, of the methods and models employed to produce them. The publication of these scripts not only ensures the transparency of this work but would also offer valuable materials to the specialized public for better scrutiny of the work and suggest further improvements. If the "WHO excess mortality model is a live model that will be periodically updated given additional data and a review of the statistical framework," it would benefit hugely from the review of the readers.

Minor issues:

• In the map in Fig 1, Puerto Rico has no data, although the CDC provides it.
• There is a reference missing on page 9: "(?)".

• It isn't easy to distinguish the colors in Fig 10.

References
Dowd, J. B., Andriano, L., Brazel, D. M., Rotondi, V., Block, P., Ding, X., Liu, Y., & Mills, M. C. (2020). Demographic science aids in understanding the spread and fatality rates of COVID-19. Proceedings of the National Academy of Sciences, 117(18), 9696–9698. https://doi.org/10.1073/pnas.2004911117
Goldstein, J. R., & Lee, R. D. (2020). Demographic perspectives on the mortality of COVID-19 and other epidemics. Proceedings of the National Academy of Sciences, 117(36), 22035–22041. https://doi.org/10.1073/pnas.2006392117
Nepomuceno, M., Klimkin, I., Jdanov, D. A., Alustiza-Galarza, A., & Shkolnikov, V. M. (2022). Sensitivity Analysis of Excess Mortality due to the COVID-19 pandemic. Population and Development Review, 0(0), 1–24.
Sasson, I. (2021). Age and COVID-19 mortality: A comparison of Gompertz doubling time across countries and causes of death. Demographic Research, 44(16), 379–396. https://doi.org/10.4054/DemRes.2021.44.16
Shkolnikov, V. M., Klimkin, I., McKee, M., Jdanov, D. A., Alustiza-Galarza, A., Németh, L., Timonin, S. A., Nepomuceno, M. R., Andreev, E. M., & Leon, D. A. (2022). What should be the baseline when calculating excess mortality? New approaches suggest that we have underestimated the impact of the COVID-19 pandemic and previous winter peaks. SSM - Population Health, 18, 101118. https://doi.org/10.1016/j.ssmph.2022.101118
Simonsen, L., Taylor, R., Viboud, C., Dushoff, J., & Miller, M. (2006). US flu mortality estimates are based on solid science. BMJ (Clinical Research Ed.), 332(7534), 177–178. https://doi.org/10.1136/bmj.332.7534.177-a
Thompson, W. W., Weintraub, E., Dhankhar, P., Cheng, P.-Y., Brammer, L., Meltzer, M. I., Bresee, J. S., & Shay, D. K. (2009). Estimates of US influenza-associated deaths made using four different methods. Influenza and Other Respiratory Viruses, 3(1), 37–49. https://doi.org/10.1111/j.1750-2659.2009.00073.x

Referee #3 (Remarks to the Author):

A. Key results: Please summarise what you consider to be the outstanding features of the work.

The study provides a broad overview of the results of the WHO TAG analysis of excess mortality during the pandemic, which was published in May (with later revisions). The work gives an extensive overview of the methods previously developed and provides various summaries and figures presenting the global estimates of excess mortality, focussing heavily on P-scores. The notable outstanding and novel features of the work are the discussion of the ranking across countries and the broader discussion of the challenges in conducting a study of this nature, the political aspects related to Member State consultation and the CRVS gaps and how that impacts the methods chosen.

B. Validity: Does the manuscript have flaws which should prohibit its publication? If so, please provide details.

There are no flaws with the methods described and the rationale behind their use, which prioritised using conventional approaches allowing for consistent models for each of the available data types and also one that allows for easier interpretation of model operating characteristics in comparison to the Economist and IHME models. This choice makes sense given the need for engaging member states through consultation and the need for predictions that can be explained.

C. Originality and significance: If the conclusions are not original, please provide relevant references. On a more subjective note, do you feel that the results presented are of immediate interest to many people in your own discipline, and/or to people from several disciplines?

The manuscript's originality is complicated given the overlap with the submitted manuscript in AAS. The manuscript does not have direct duplication in terms of the figures presented (trends in excess over time and global maps), with the Nature submission focusing on plotting excess deaths in terms of P-scores, whereas the AAS submission plots total excess deaths per capita. However, I feel that the content underlying both submissions is fundamentally the same, with different metrics chosen to present and different regional summaries used.

The ranking analysis, however, is original and touches on important wider concerns about the suitability of country comparisons. This area of the manuscript I think has considerable merit and in any resubmission I would focus more on how excess mortality and COVID-19 mortality statistics have resulted in unhelpful country comparisons that distract from data transparency and effective public health responses. Given the supranational role of the WHO, this area of the study and the steps taken and considered by the TAG in how to "depoliticise" COVID-19 and excess mortality reporting and engage member state buy in would be original and highly important for shaping how we improve transparency during the next pandemic.

D. Data & methodology: Please comment on the validity of the approach, quality of the data and quality of presentation. Please note that we expect our reviewers to review all data, including any extended data and supplementary information. Is the reporting of data and methodology sufficiently detailed and transparent to enable reproducing the results?

The quality of the data and the steps taken by the authors to consult and engage with countries is commendable and has resulted in a valuable data product in its own right. However, the absence of any Data availability and sharing section or reproducible codebase is an unacceptable omission. I appreciate that some of the data shared with the WHO is not available to share, there must still be a reproducible code base (any data not able to share could be omitted and the model fit object shared but with the data used in fitting removed) in any revised submission (whether that is included in the AAS submission or any revised submission, it must be somewhere).

E. Appropriate use of statistics and treatment of uncertainties: All error bars should be defined in the corresponding figure legends; please comment if that's not the case. Please include in your report a specific comment on the appropriateness of any statistical tests, and the accuracy of the description of any error bars and probability values.

Descriptions of uncertainty intervals are missing in all figure legends (except for Figure 10). The use of statistical summaries and description of the uncertainty generation process in the methods and how this is contrasted against IHME and Economist is very well written. The ranking methodology is interesting and well described and I think very useful for conveying the difficulty in making country comparisons of excess mortality (especially when based solely on central estimates).

F. Conclusions: Do you find that the conclusions and data interpretation are robust, valid and reliable?

Yes, the conclusions are robust, valid and reliable (however I would still want to see a code base and to check this through).

G. Suggested improvements: Please list additional experiments or data that could help strengthening the work in a revision.

One of the more interesting findings in the AAS submission is the comparison across IHME and the Economist. Focussing just on comparisons against the Economist model (which is more defensible methodologically in my opinion), the WHO model produces overlapping totals for all regions except for EMRO. This finding is not discussed in either manuscript and should be. Understanding what is driving those differences and critically reviewing the estimates made in EMRO countries should be done. Do the authors have a sense of why this is the case? Is the reliance on covariate imputation based on EMRO medians, which will draw on covariates from higher income countries for LICs?

Related to this, I think a particular extension to the model framework should be the inclusion of seroprevalence data. Representative seroprevalence data has been shown to be highly predictive of excess mortality in many countries (e.g. Ghafari et al 2022. Nat Comms) and would be a valuable covariate to incorporate. I am aware that seroprevalence is more sparse than all cause mortality data, but it could be incorporated in a number of ways. Possibly the simplest would be to infer the expected seroprevalence nationally/regionally based on reported COVID-19 deaths and an assumed IFR based on IFR by age patterns. The ratio of expected seroprevalence against observed seroprevalence could be incorporated as a time-invariant variable. If multiple seroprevalence data exist, then extensions to acknowledge that COVID-19 death ascertainment may change over time. Regional/urban seroprevalence data could similarly be used when regional/urban data are available.

Alternatively, seroprevalence could be inferred from the predicted excess mortality estimates that have been produced (assuming all excess mortality is COVID-19) and compared against seroprevalence data. And if the only good seroprevalence data is subnational/in cities then making simple assumptions that subnational transmission is comparable to national transmission. This would show that in some EMRO countries, e.g. Somalia, the excess mortality aligns closely with seroprevalence (e.g. in Benadir, Adam et al 2022. Vaccines) but in Syria the estimates appear too low.

Lastly, there are a number of subnational studies that have produced estimates of changes in mortality during COVID-19 in LICs that could be interesting to consider in Discussion for comparison. E.g. Koum-Besson et al BMJGH 2021, or Watson et al. 2021 Nat Comms, which have mortality data

sets, which although not all cause mortality, could be used to produce P-scores to compare against the predictions. (There are more examples, but have just focussed on those I am aware of in EMRO regions).

H. References: Does this manuscript reference previous literature appropriately? If not, what references should be included or excluded?

Overall referencing is good. Discussion could be extended to review the results in the context of other mortality studies (as above) to evaluate the predictions.

I. Clarity and context: Is the abstract clear, accessible? Are abstract, introduction and conclusions appropriate?

Manuscript needs to be re-written to be more succinct - much of the methods are in AAS, and a shorter description could be used in the Methods here.

Author Rebuttals to Initial Comments:

Response to Editor and Reviewers on "Estimates of the excess mortality associated with the COVID-19 pandemic from the World Health Organization"

1. *As we discussed in the past, it is important to include in the Nature paper a certain level of detail about process, methods and data, to make the paper as self-contained as possible. As the referees point out, however, right now these discussions take a lot of space right at the beginning of the paper. Given the existence of the companion AAS work, the Nature paper should really focus on the results and their discussion. As such, the process/methods/data sections in the body of the main paper should be reduced considerably and their current shape (which, I think, reads really well) moved to the methods section.*

   We have followed this suggestion and moved the process/methods/data sections to the methods section, and included a short summary in the main paper.

2. *In the revision you will have to include a data and code availability statement providing detailed information of what data/code is available or what is not, and how can readers and scholar access it. Given the high-profile of this publication we would naturally strongly encourage you to make as much content available as possible for transparency.*

   We have created a github site for all the code and data: `https://github.com/WHOexcessc19/Codebase`, which allows the estimates to be reproduced.

3. *Reviewers #1 and #2 bring up the matter of Germany, Sweden and the spline interpolation. It seems important to include this discussion in the Nature paper: while the details of the interpolation are and should remain in the methods paper, the effect of this choice, its limitations and impact seems to be much more relevant to the Nature paper.*

   We have moved the discussion of the cases of Germany and Sweden out of the AoAS paper and into the Results section of this paper. The details in the supplement of the AoAS paper have also been moved to the Nature supplement.

4. *Reviewer #1 believes India deserves to be highlighted and discussed in the Nature paper. We generally agree but we can find a middle ground on this given that you decided not to remove it from the AAS paper.*

We have moved one of the summary plots for India and the comparison of estimates from different data sources from the AoAS papers to the supplement, and provided some discussion in the text.

5. *An in-depth technical comparison against IMHE and Economist models is better placed in the AAS. But all reviewers point out that this is something that everyone reading the Nature paper will wonder about, so it would be best to expand on it and better highlight the advantages of your approach over theirs.*

   In the methods section we summarize the differences, and then in the supplementary materials we provide a comparison of point and interval estimates, by country (4 figures, with discussion).

6. *The reviewers found the rankings section quite stimulating, but they also point out that it's quite disconnected from the rest of the paper. We believe – also in the interest of starting to work to bring your paper in line with our formatting requirements – that a full section devoted to rankings may be excessive. This, with its figures, can be moved to Methods or supplementary information. But it would be very important to summarize the main take-home points of this section within the paper itself in order to support some of the broader points that your paper is making regarding the impact and management of the pandemic.*

   We haves added a discussion of uses of the modeling and methods in main paper, and then added the rankings discussion to the methods section. We have also included a more substantive rankings example to the supplement.

7. *Finally, we need to work on the title and abstract of your work (and, ideally, of the AAS paper) to ensure that the Nature paper's comply with our limits (title: 75 characters including spaces). Abstract: 230 words and it has to include references) and that the two papers are sufficiently distinct as not to be confused by someone searching for them.*

   We have provided a new abstract.

On the formatting front, I am going to ask you to start bringing the paper towards the shape of a nature article, which has a maximum of 6 display items (tables and figures), a maximum of 10 Extended display items (they will appear on the html and appended to the PDF, but will not be within

the paper) and a methods section that shouldn't be more than around 3000 words long. Everything else can be included in a separate supplementary information PDF on which we have no restrictions.

There are now 6 figures in the main paper, and 4 in the methods section.

In terms of the length of your paper, I will not ask you to cut any text but simply to rearrange it such that some sections end up in the methods section, and the main body of the paper is as streamlined as possible in delivering the key findings for the nature paper (Reviewer #1 has good suggestions for this). Please aim at a length of around 3000–3500 words but if this is very challenging contact me and we can discuss how to do this best.

Referee #1 (Remarks to the Author):

Proper ascertainment, via state-of-the-art estimation methods, of excess mortality for the pandemic years, at this time 2020 and 2021, is of the utmost important to gauge the impact of the pandemic on a very important parameter. This paper is not the first to address this important problem but, in my view, has the potential to be the most successful of all to date. Well-known attempts are that of IHME and The Economist. The authors voice justified criticisms against these two approaches and then try to overcome them in their own approach. These are:

- *Models that are arguably too simplistic, in view of the large heterogeneity in data availability and quality – this applies predominantly to IHME; in my view, to the point that the IHME approach should be discarded.*
- *Models that do take into account national and regional differences, but are not sufficiently methodologically principled, perhaps even black box – this applies to The Economist. While this criticism is justified, the estimates from The Economist are widely considered plausible.*

Thanks, we agree with these summaries.

- My main concern with the paper as it stands now is a certain lack of organization. Even though addressing it might require considerable editing work, it is not a methodological criticism. Slightly overstating the issue, the Nature submission might come across as 'a less technical version of the companion paper'. I will expand on this and offer some suggestions for redistribution and reorganization of the material. Related to this, the Nature submission should have a more crisp structure.

Thanks for your suggestions. We have carried out a major restructuring of the paper, based on your comments and those of the editor and other referees.

- *Background: Problem statement.*

- *Everything to do with data and the problems with the data: this involves the current Process and Data sections. Regarding data availability, I would like to see a clear statement regarding the issues that the modeler is confronted with. Ideally, data are of high quality, available quickly and without interruption, nationally and regionally, and at sufficiently short*

time intervals (e.g., monthly). Then, everything that deviates from this is a problem that the modeler needs to overcome, with clever, appropriate methods, and inevitable with plausible but unverifiable assumptions. I think that the authors do a very good job in this regard, but I am not sure the Nature reader will easily appreciate this.

We added, "Ideally, we would have all-cause mortality data for all countries and for all months. The reality is that such monthly national data are only available for 100 countries (52%) with other countries having annual data, subnational data or no data. For the latter three cases, we predict the monthly data within a Poisson count model framework, as detailed in the Methods section."

- *Methodology: in the main Nature paper, there is a bit too much statistical jargon (even though most statisticians would disagree with this perhaps). The methodology should be presented continually with an eye on how the above issues are overcome. I would be inclined not to present formulas in the main paper, but describe how various modeling tools are used to work with 'sub-optimal data' to satisfaction. The formula-based pieces could be assembled in the Nature manuscript's appendix/supplement.*

  The formulas have now been placed in the Methods section.

- *Related to the above, I would suggest to explain some of the issues that occur based on a set of countries that are now discussed in the companion paper. What is now in the companion paper on page 21 on Germany and Sweden, I would tend to move to the main Nature paper. Likewise, the Indian case is extremely important, from a methodological perspective (can be elaborated form that angle in the companion) and from a mortality perspective (should go in the Nature submission).*

  We have moved the Germany and Sweden discussion and some aspects of the Indian analysis from the AoAS paper to the Nature submission.

- *When presenting methods, the Nature submission should spell out which covariates and auxiliary information are used. This should all come at once, and not dotted around the paper(s). For example, as a reader I would like to know quickly whether reported COVID-19 mortality is used, whether seasonality/temperature is used, whether confirmed cases, hospitalizations, ICU occupancy, are used, etc. Likewise, the age and gender structuring should be discussed.*

With all of these, brief motivations as to why or why not these are used, would be extremely useful.

We have included the details about the covariates used for the model in both the summarized "Process, data and methods" section as well as the full detailed version found in the detailed methods section. In both descriptions, the variable details are in one place. The age and sex model is a work in progress and methods for it are not included in this paper nor are any age and sex disaggregated estimates.

- *The main focus should be on the results and interpretation thereof. This will connect seamlessly to the discussion of some specific countries in the previous item.*

  This is what we have aimed for in the latest version.

- *The results should be contrasted with those by other efforts (IHME, The Economist, but also others). Especially for The Economist, it would be good to move beyond the mere (and justified) comment that the method is not sufficiently theoretically backed up. In other words, 'it seems to work' and a case in point is that the ratio of 2.75 in the current work is not too far away from the about 3.0 to 3.3 that The Economist has been obtaining. I realize that such a comparison is given in the companion paper, but I suggest to discuss it (predominantly) in the Nature submission. That actually strengthens the current paper and does not subtract from the fact that, in the long run, the current method might be more sustainable.*

  We have added country-level comparisons between the estimates (point and interval) in the Supplementary Materials. In the Supplementary Materials, we have also included a table of excess estimates to reported COVID-19 globally and by region, from each of WHO, The Economist and IHME. There are quite large differences in EMRO, AFRO AND WPRO, which we highlight.

- *Returning to India, some interesting developments are in the supplement of the companion paper. A broadly accessible narrative belongs, in my view, in the Nature submission – perhaps in its Supplement.*

  We have followed this suggestion.

- *The section on ranking is a bit a digression. At the same time, it is extremely important as every effort should be made to warn against over-interpretation of statistical estimates by properly taking uncertainty into account. This problem is not new, of course, and pops up all over empirical research (e.g., hospital performance). The problem has exacerbated during the pandemic, so it is very opportune to talk about it here. I would therefore make it a more prominent part of the paper, by referring to it briefly but clearly in the Abstract, Introductory sections, and Discussion. A disadvantage is that we do not get a simple, one-dimensional ordered list. This should be explained and it should be clear to the reader that it is simply unavoidable. The current statement about a 'two-dimensional summary/projection of a 6-D object', is likely not going to do the job.*

  The rankings work is briefly discussed in the main paper (Results section) with the example in the previous section being moved to the Methods section. We have also added a more substantive example to the Supplementary Materials.

Some further specific comments

1. *The large table in Appendix B is immensely useful and I already look forward to the 2022 update. Unless I overlooked it, I was missing the United Kingdom. I would like to see a bit of discussion around negative estimates, i.e., under mortality. Of course, this might occur because of poor data availability – then usually accompanied by wide intervals. But there are some countries where this is the case because of policy. A good example is Norway, where the negative estimate remains even if we add 2020 and 2021. It is at stark contract with Sweden where the combined estimate is 11,255; Denmark and Finland are in between with a total of 3000 to 4000. In Sweden, the toll is higher in 2020, whereas in the others it is the other way around. Given the endless debate about Sweden's approach, this paper's modeling effort offers the authority to briefly discuss the Nordic countries. I left out Iceland, in spite of its two negative estimates, given the extremely small size of the country.*

   The UK has a 'The' in front. In the discussion, we state how "...excess mortality may provide a reliable lower-bound on COVID-19 deaths considering that for several countries, we have mortality deficits or negative estimates for certain months. The greater number of these countries have high quality reporting systems and this deficit is due to deaths from non-natural and natural causes decreasing during the analyzed period and there having been less severe influenza seasons in 2020 and 2021 relative to previous years." We did not add more specifically on the Nordic countries beyond the mention of Sweden in the Germany-Sweden section for two reasons. Firstly, most of the differences across models for these very strong

reporting systems will be in the assumptions made for the expected deaths. We detail the spline vs linear sensitivity and note that the expected changes are minimal. However, the second reason is that beyond the methods we've applied to fill the gaps in places with weaker systems, we would like to echo the clarion call for greater investment in CRVs systems in these countries that are lagging behind. We want this paper to strongly advocate for this.

2. *In terms of context, it might make sense to give some brief comparisons with historic sources of excess mortality. Of course, the further we go back in time, the less reliable the sources become, but we have, for example, influenza epidemics in the 1950 and 1960 (e.g., Hong Kong flu), World War II, and the Spanish flu.*

   We have added a short comparison in the discussion. We compare the COVID-19 pandemic to previous influenza pandemics.

3. *Aron and Muellbauer (2020), referenced in the paper, provide early estimates (Spring 2020 wave) of underreporting of COVID-19 related mortality in a small set of countries. For countries like Spain and Belgium, the results in this paper for 2020 seem to be in line with their estimate (which, admittedly, does not address excess mortality but true COVID-19 deaths). For the Netherlands, even when accounting for a discrepancy between total COVID-19 deaths and excess mortality, this paper's estimate for 2020 seems to be a bit optimistic.*

   The Netherlands estimates of excess for the years 2020 and 2021 for this paper are 14.5K (UI 12.6K, 16.2K) and 29.2K (UI 26.4K, 31.8K), respectively. While slightly lower than the corresponding Economist estimates of 16.4K and 33K for the same period, the difference is not implausible given the sensitivity of the estimate to the underlying assumptions when deriving the Expected deaths. Comparing monthly estimates of excess for these two models as well as the WMD (Table 1 below), the WHO estimates are not systematically lower.

month	Economist	WHO	WMD
1	-168	-96	-1,107
2	-957	-1,314	-1,225
3	3,650	2,642	2,331
4	5,154	5,725	6,089
5	105	372	164
6	-208	-412	-221
7	-317	-350	-517
8	882	786	726
9	447	201	294
10	1,992	2,326	1,980
11	2,695	1,919	2,226
12	3,095	2,682	3,334
13	2,152	2,955	2,029
14	490	-482	216
15	-706	-323	-720
16	892	503	855
17	783	750	633
18	372	179	481
19	382	505	380
20	1,110	923	979
21	1,138	807	1,130
22	1,401	1,679	1,553
23	5,206	3,747	4,281
24	3,372	3,486	4,150

Table 1: Comparing estimates of excess estimates by month across 3 models for the Netherlands

4. *A very powerful message is that the excess mortality is higher in 2021 than in 2020. For this reason, having 2020 and 2021 columns in Table 1, in addition to the combined estimates, would be useful, pretty much as is the case in the Appendix table. Some comments as to the reasons for this would be welcome. We started vaccinating in 2021, but there was vaccine hesitancy, equity problems, waning, increasingly pathogenic variants (Alpha and Delta in particular in 2021, but some others in Latin America, such as Lambda and Mu), and less support in policy makers and general public for non-pharmaceutical interventions.*

   We have extended the table, as suggested and added text in the discussion looking at this – in the paragraph which begins with, "As shown in the supplementary materials, there is a more than doubling of excess deaths when comparing 2021 to 2020...".

5. *The message in the Disclaimer is important. In some countries, there has been pressure on researchers not to report or 'report optimistically' mortality. It will be comforting for the reader to know that the current work has been done without the influence of any such pressures. I have no reason to doubt this personally, though.*

6. *In Europe, EUROMOMO has been monitoring (excess) mortality in a number of countries or regions. Would this be a worthwhile source?*

   EUROMOMO is a worthwhile source for validating/benchmarking the estimates seeing as they are generally accepted in Europe. For some of the countries, the same individuals supply data to EUROMOMO and WHO. However, they apply different assumptions on how the data are aggregated and length of time-series used to generate the expected etc, which are not entirely clear.

7. *The authors indicate how they deal with aligning national and subnational data, when both are available. Has there been any adjudication in cases where there were blatant discrepancies, in case the problem occurred.*

   We didn't have situations in which we looked at both subnational and national data, so this has not arisen.

8. *The authors correctly indicate that countries with good and poor reporting are not distributed uniformly around the globe. Still, would there be any hope to use geographical proximity (hence, spatial methodology) to borrow information from well-reporting countries in the neighborhood to inform those with data of lesser quality?*

   That is a possibility that we have discussed as a group (and we mention in the discussion of the accompanying paper).

9. *The section starting on page 8 is an illustration of my earlier point where methodological presentation should be re-thought. This is a point where the interested but less technical reader will be lost.*

   This material has been moved to the Methods section.

10. *Page 9, penultimate paragraph: unresolved LaTeX reference.*

    Fixed.

11. *When stating that you have data on 17 Indian states, it would be good to state the total number as well.*

    Done.

12. *Explain what you mean by 'generative model' if the term is going to be preserved.*

    We have removed this term.

13. *The reader might be lost over 'and with the models for different data types being consistent with each other'.*

    We have expanded on this sentence and added, "As an example, if the mortality in subnational regions are Poisson random variables, then the sum (the mortality in the country) is also Poisson. Further, given the total mortality in a country the subnational counts follow a multinomial distribution. Our framework exploit these relationships when we formulate models for the situation in which we have subnational data only. Similarly, our annual model (for countries with such data only) is consistent with the monthly models we use for the majority of the countries."

14. *The P-score, with its shortcomings, is a useful measure and happy to see it reported in the Appendix table. Explain it as non-technical as possible. The technical reader can easily access one of the technical references, or you could even explain it formula based in the Supplement.*

    We have moved the mathematical description to the Methods section.

15. *Page 12, line -13: There is nothing wrong with the sentence starting with 'This sharp increase...' yet many people might have to read it a few times.*

We have reworded as, “This sharp increase is almost entirely due to the catastrophic wave that hit India at this time.”

16. *Page 12, line -10: state → states.*

    Done.

17. *Figure 7, make the legend more descriptive.*

    Done.

18. *Discussion, line 2: also here, give numbers and intervals for 2020 and 2021 separately. Of course, the interval for 2020+2021 will not simply be the sum of the other intervals, but the point estimates will.*

    This is a good idea and we have included.

19. *Discussion, line 2: avoid ‘significant’ in this slightly non-technical sense.*

    We have replaced with ‘dramatic’.

20. *Here, the 2.74 should be contrasted with (at least) the corresponding The Economist estimate. This number alone, and the ensuing estimate of the total excess mortality for the years 2020 and 2021 should be estimated in an authoritative way and this paper has the potential to do it.*

    In Tables 5 and 6 of the supplementary information we compare the excess estimates from this paper to those of IHME and the Economist for the year 2020 and the combined 2020 to 2021 period, respectively. We compare using two different measures, firstly the absolute count and secondly, the ratio of the excess deaths to the reported COVID-19.

21. *I like the careful discussion in the middle of page 22 on the attribution of excess deaths to COVID-19. Not everyone on the planet will be convinced, but it is a sound conclusion, based on proper research. One may also want to discuss harvesting: in some countries, with high*

mortality in 2020 and high quality reporting, one often sees a much lower excess mortality in 2021, and a bigger discrepancy between COVID-19 and excess deaths – against the dominant trend. This is the case for Sweden, Belgium, France,...

Thank you, one can expect excess mortality to provide a lower bound on the true number of COVID-19 deaths. In other words, we speculate that whenever COVID deaths are counted perfectly, they should exceed the excess mortality, leading to undercount ratio below 1. This is indeed what we observed in several countries with strong COVID-19 outbreaks but accurate accounting of COVID deaths, for example Belgium, France, and Germany (undercount ratios 0.6, 0.7, and 0.4, respectively). And we have looked at the two year period as separate years as well as cumulatively. Using the cumulative sum accounts for 'mortality displacement' according to Islam et al. (2021) who have a brief description of this: https://www.bmj.com/content/373/bmj.n1137

22. *On page 23, second half, reference is made to mortality as part of a monitoring system. I agree with this if properly qualified. I think it is clear that this will not be a component of an early-warning system. For that, we need GP workload, wastewater surveillance, genomic surveillance, and general monitoring of emerging pathogens. It will play an important role when a health crisis is ongoing. For example, if we would have had this model in the middle of 2020 and later, it could have contributed to counterargue the 'it has become a mere flu-like condition' argument. At least until now, The Economist results have played this role to some extent.*

    The point here is that as in Europe that has a more advanced system able to identify the excess attributable to seasonal influenza and other shocks, more advances need to be made for such surveillance capacity to be present in poorer countries. This would be a system that is more readily available for more countries as compared to waste-water tracking or genomic sequencing for which many countries have even less capacity. The advantage of the mortality surveillance being this generalisability with one not needing to know what to look for specifically beyond noting that there must be a problem leading to an excess of deaths beyond expected. Next steps would be investigating the specific pathogen.

23. *In this regard, the shiny app is a wonderful tool.*

    Thanks!

Referee #2 (Remarks to the Author):

The paper presents estimates of excess mortality, directly and indirectly, attributable to COVID-19 during the years 2020 and 2021. It is a significant contribution to the analysis and assessment of the global mortality burden of the pandemic. There is a massive work behind these analyses in data collection, curation, modeling, and analysis of the estimates. In general, the approach, data quality, and presentation quality are valid, although there are a few suggestions to improve the modeling strategy. The statistics and treatment of uncertainties seem appropriate. The use of a Bayesian approach, integrating prior uncertainty, is convenient. The manuscript is very well written. The abstract, introduction, and conclusions are lucid and appropriate.

The authors take a considerable risk in estimating excess mortality in countries where information on all-cause mortality is unavailable in the years 2020 and 2021. Although this estimation implies substantial potential biases, the authors acknowledge these risks well and do the best possible with the available resources. In this sense, the exercise is essential and hopefully a good approximation to the worldwide mortality crisis caused by the pandemic. The call for improving monitoring systems, vital statistics, and civil registrations is very pertinent and much needed.

Major issues:

In general, I think the models are robust. However, I am concerned about the parameterization for fitting the baseline mortality. For fitting monthly mortality, the authors use a thin-plate spline for the "annual trend" and a cyclic cubic spline for the seasonable component. Splines are great for interpolation but generally very risky for extrapolation. Splines extrapolation only uses the last fitted coefficients, depending on the order of difference selected for fitting the spline. The authors do not give information regarding the order of difference parameterized for the spline fitting. However, according to the information provided in the methodological paper, the fitting was done using the default options of the mgcv package, which uses 2nd order difference for the splines. Under a 2nd order difference parameterization, the model linearly extrapolates based on the last two coefficients. This extrapolation has the risk of generating inadequate baselines in several cases. To avoid this, I would suggest using a safer option, such as a log-linear parameterization for the secular change in mortality. In the methodological paper, the authors mention this issue and note that adjustments of this kind were made to Germany and Sweden but not to the other populations. Those adjustments and the identified limitation should be acknowledged in this manuscript. It should be justified why the log-linear trend was exclusively applied to two countries and not to all of them. I can think of many reasons for using splines for interpolation, but none that justifies using splines for extrapolation. If the authors see the use of splines as an advantage over a simpler log-linear trend, it should

be justified.

As we discuss in the AoAS paper with hindsight we would have expended more effort on selecting a model for the baseline mortality, and we will do this before producing the next round of estimates. Saying that, we have examined more carefully the excess estimates and do not believe that replacing the spline model will make a substantive difference this time in many countries. In the Discussion we have added, "We used spline models as the basis for the modeling of the expected numbers, but will revisit this choice for the next round of estimates, since such models can produce inappropriate extrapolations."

The selection of contextual variables for extrapolating the estimates to countries with no data seems adequate, as they take into consideration proxies for COVID-19 activity and severity (confirmed deaths, positivity rate, etc.), country-specific containment measures, and different indicators of population-specific vulnerability to COVID-19 (cardiovascular and diabetes prevalence, and income). Of course, it is always possible to include a large number of variables, as it is done in other modeling attempts (e.g., Economist). Still, I think the conservative approach of the authors is a wise decision. However, there seems to be a critical omission of one of the most important determinants of the risk of death in a population in the context of COVID-19: information on the proportion of people at old ages in the population (Dowd et al., 2020; Goldstein & Lee, 2020; Sasson, 2021).

We examined the use of the proportion of the population over the age of 65, but the distribution of this covariate was very different in countries with data and those without data, and so we were nervous about the use of this covariate. However, the covariate model is 'live' and we plan to revisit the covariate choice for the next round of estimates.

Other issues:

The authors propose a proportionality assumption for countries where data is restricted to subnational regions. According to this hypothesis, the subnational share of ACM is the same before and during the pandemic. However, there is no evaluation of how plausible is this assumption, or at least a mention of this. With the available data, it would be possible to evaluate this assumption in countries where complete monthly data is available at the national and subnational levels (e.g., the US). Such sensitivity analysis would inform the potential bias under the proportionality assumption.

In the accompanying paper we carry out extensive sensitivity analyses for India and for Argentina, where we have both national and subnational data during part of the pandemic.

If the excess is estimated annually and no attempts are made for influenza correction, it is unclear why it is essential to use monthly data for the baseline mortality estimation. Fitting annual data is more straightforward than monthly data, and the estimates are consistent (Nepomuceno et al., 2022). I suppose the importance of this temporal resolution could be related to the time-varying variables used for extrapolating the estimates to countries with no data (?). However, this is my guess, and I am not even convinced about it. I think there is a need for more clarity in this respect, as the use of monthly data makes the modeling considerably more complex and implies strong assumptions for the countries with no monthly data.

We have used the monthly model with expected values that incorporate both annual and seasonal trends, this to account for historical seasonal increases or decreases in mortality including what would be due to regular influenza or heat wave shocks. Any excess influenza beyond what is normally experienced would be the target of this study. Although an annual model would be more straightforward, we would lose a significant amount of information on the drivers of excess mortality by averaging the covariates to their annual summaries. The information is not only in the levels of the variables considered but also their temporal association with excess. This in turn would restrict the inference to the locations with limited data if for example we only have annual containment, annual test positivity and annual reported COVID-19. Additionally, COVID-19 mortality was experienced in waves, yielding high peaks in some periods but approximating expected mortality in the rest of the year. Using just annual counts would potentially under-estimate excess in months without COVID-19 (start of 2020 for example). Where high-frequency data do not exist, we indeed use annual mortality counts as second best.

In addition, the model for partitioning annual death counts to monthly is poorly described. According to the methodological paper, the partition is done using a model including information on temperature. Therefore, it would be important to mention the use of temperature in this paper briefly. This mention is essential because the inclusion of temperature guarantees that the monthly partition follows the divergences in seasonality across countries.

We now include the following in the Methods section, "For some countries, we only have national historic ACM data. For such countries we model within-year variation using temperature as a surrogate for seasonality, full details are given in Knutson et al. (2022)".

The model assumes influenza mortality in 2020 and 2021 to be similar to the exceptionally severe seasonal epidemics in 2015-2019. The authors acknowledge that there is evidence that influenza circulation was exceptionally low during 2020 and 2021, which implies a considerable underestimation of excess mortality. Still, there is no attempt to adjust this bias. This wouldn't be problematic

if there were no way to take into consideration the exceptional low influenza mortality, but there are several excess mortality models that would allow for this (Simonsen et al., 2006; Thompson et al., 2009) and correct for a bias that seems to be non-negligible in the context of the COVID-19 pandemic (Shkolnikov et al., 2022). The use of monthly data would allow for such adjustments.

This is a limitation of the access to limited historical data that we had. The next iteration of this work will incorporate a longer historical time-series which will allow us to identify and quantify the non-typical excess attributable to more severe influenza for the 2015–2019 period. We would consider a shock free expected in that case more robust to recent fluctuations. The exploration of the linear vs spline fit for the annual trend will also contribute to our improving the model in the next iteration.

Last but not least, it is excellent that the WHO publishes the mortality estimates by country in the shiny app. The authors state, "This tool allows transparent exploration of estimates from the country level up to the regional and global level." However, there is no access to the scripts that were employed for the estimation of excess mortality. The publication of the scripts to reproduce the analysis presented here would allow for a transparent exploration not only of the outputs but, more importantly, of the methods and models employed to produce them. The publication of these scripts not only ensures the transparency of this work but would also offer valuable materials to the specialized public for better scrutiny of the work and suggest further improvements. If the "WHO excess mortality model is a live model that will be periodically updated given additional data and a review of the statistical framework," it would benefit hugely from the review of the readers.

The scripts for model fitting are now available at `https://github.com/WHOexcessc19/Codebase`.

Minor issues:

- *In the map in Fig 1, Puerto Rico has no data, although the CDC provides it.*

  The current study is restricted to the 194 WHO member states. Puerto Rico does not fall within this grouping, similar to French overseas departments, Hong Kong, Taiwan and the Palestinian Authority. In future we will be looking at these additional territories.

- *There is a reference missing on page 9: "(?)".*

  Fixed.

- *It isn't easy to distinguish the colors in Fig 10.*

  We have changed the color scheme.

References

Dowd, J. B., Andriano, L., Brazel, D. M., Rotondi, V., Block, P., Ding, X., Liu, Y., & Mills, M. C. (2020). Demographic science aids in understanding the spread and fatality rates of COVID-19. Proceedings of the National Academy of Sciences, 117(18), 9696-9698.
https://doi.org/10.1073/pnas.2004911117

Goldstein, J. R., & Lee, R. D. (2020). Demographic perspectives on the mortality of COVID-19 and other epidemics. Proceedings of the National Academy of Sciences, 117(36), 22035–22041.
https://doi.org/10.1073/pnas.2006392117

Nepomuceno, M., Klimkin, I., Jdanov, D. A., Alustiza-Galarza, A., & Shkolnikov, V. M. (2022). Sensitivity Analysis of Excess Mortality due to the COVID-19 pandemic. Population and Development Review, 0(0), 1–24.

Sasson, I. (2021). Age and COVID-19 mortality: A comparison of Gompertz doubling time across countries and causes of death. Demographic Research, 44(16), 379-396.
https://doi.org/10.4054/DemRes.2021.44.16

Shkolnikov, V. M., Klimkin, I., McKee, M., Jdanov, D. A., Alustiza-Galarza, A., Németh, L., Timonin, S. A., Nepomuceno, M. R., Andreev, E. M., & Leon, D. A. (2022). What should be the baseline when calculating excess mortality? New approaches suggest that we have underestimated the impact of the COVID-19 pandemic and previous winter peaks. SSM - Population Health, 18, 101118.
https://doi.org/10.1016/j.ssmph.2022.101118

Simonsen, L., Taylor, R., Viboud, C., Dushoff, J., & Miller, M. (2006). US flu mortality estimates are based on solid science. BMJ (Clinical Research Ed.), 332(7534), 177-178.
https://doi.org/10.1136/bmj.332.7534.177-a

Thompson, W. W., Weintraub, E., Dhankhar, P., Cheng, P.-Y., Brammer, L., Meltzer, M. I., Bresee, J. S., & Shay, D. K. (2009). Estimates of US influenza-associated deaths made using four different methods. Influenza and Other Respiratory Viruses, 3(1), 37-49.https://doi.org/10.1111/j.1750-2659.2009.00073.x

Referee #3 (Remarks to the Author):

A. Key results: Please summarise what you consider to be the outstanding features of the work.

The study provides a broad overview of the results of the WHO TAG analysis of excess mortality during the pandemic, which was published in May (with later revisions). The work gives an extensive overview of the methods previously developed and provides various summaries and figures presenting the global estimates of excess mortality, focussing heavily on P-scores. The notable outstanding and novel features of the work are the discussion of the ranking across countries and the broader discussion of the challenges in conducting a study of this nature, the political aspects related to Member State consultation and the CRVS gaps and how that impacts the methods chosen.

B. Validity: Does the manuscript have flaws which should prohibit its publication? If so, please provide details.

There are no flaws with the methods described and the rationale behind their use, which prioritised using conventional approaches allowing for consistent models for each of the available data types and also one that allows for easier interpretation of model operating characteristics in comparison to the Economist and IHME models. This choice makes sense given the need for engaging member states through consultation and the need for predictions that can be explained.

C. Originality and significance: If the conclusions are not original, please provide relevant references. On a more subjective note, do you feel that the results presented are of immediate interest to many people in your own discipline, and/or to people from several disciplines?

The manuscript's originality is complicated given the overlap with the submitted manuscript in AAS. The manuscript does not have direct duplication in terms of the figures presented (trends in excess over time and global maps), with the Nature submission focusing on plotting excess deaths in terms of P-scores, whereas the AAS submission plots total excess deaths per capita. However, I feel that the content underlying both submissions is fundamentally the same, with different metrics chosen to present and different regional summaries used.

We have now removed some of the results and discussion on Sweden, Germany and India from the AoAS paper and put into this submission, and expanded the rankings discussion, as well as added to the interpretation of the results in various places. We now feel that the papers are more clearly delineated.

The ranking analysis, however, is original and touches on important wider concerns about the suitability of country comparisons. This area of the manuscript I think has considerable merit and in any resubmission I would focus more on how excess mortality and COVID-19 mortality statistics have resulted in unhelpful country comparisons that distract from data transparency and effective public health responses. Given the supranational role of the WHO, this area of the study and the steps taken and considered by the TAG in how to 'depoliticise' COVID-19 and excess mortality reporting and engage member state buy in would be original and highly important for shaping how we improve transparency during the next pandemic.

The rankings discussion is now expanded both in the main body of the paper and in the Supplement.

D. Data & methodology: Please comment on the validity of the approach, quality of the data and quality of presentation. Please note that we expect our reviewers to review all data, including any extended data and supplementary information. Is the reporting of data and methodology sufficiently detailed and transparent to enable reproducing the results?

The quality of the data and the steps taken by the authors to consult and engage with countries is commendable and has resulted in a valuable data product in its own right. However, the absence of any Data availability and sharing section or reproducible codebase is an unacceptable omission. I appreciate that some of the data shared with the WHO is not available to share, there must still be a reproducible code base (any data not able to share could be omitted and the model fit object shared but with the data used in fitting removed) in any revised submission (whether that is included in the AAS submission or any revised submission, it must be somewhere).

The updated codebase includes all the data used in the model, i.e., the country consulted data as well as the data from other sources and all the code: `https://github.com/WHOexcessc19/Codebase`.

E. Appropriate use of statistics and treatment of uncertainties: All error bars should be defined in the corresponding figure legends; please comment if that's not the case. Please include in your report a specific comment on the appropriateness of any statistical tests, and the accuracy of the description of any error bars and probability values.

Descriptions of uncertainty intervals are missing in all figure legends (except for Figure 10). The use of statistical summaries and description of the uncertainty generation process in the methods and how this is contrasted against IHME and Economist is very well written. The ranking

methodology is interesting and well described and I think very useful for conveying the difficulty in making country comparisons of excess mortality (especially when based solely on central estimates).

We have now included descriptions of the uncertainty intervals for each figure.

F. Conclusions: Do you find that the conclusions and data interpretation are robust, valid and reliable?

Yes, the conclusions are robust, valid and reliable (however I would still want to see a code base and to check this through).

As indicated above, we have created a site for all the code and data: `https://github.com/WHOexcessc19/Codebase`

G. Suggested improvements: Please list additional experiments or data that could help strengthening the work in a revision.

One of the more interesting findings in the AAS submission is the comparison across IHME and the Economist. Focussing just on comparisons against the Economist model (which is more defensible methodologically in my opinion), the WHO model produces overlapping totals for all regions except for EMRO. This finding is not discussed in either manuscript and should be. Understanding what is driving those differences and critically reviewing the estimates made in EMRO countries should be done. Do the authors have a sense of why this is the case? Is the reliance on covariate imputation based on EMRO medians, which will draw on covariates from higher income countries for LICs?

In the Supplementary Materials, we now provide plots of point (and interval estimate width) estimates against each other for all 194 countries, i.e., WHO versus IHME and WHO versus Economist, and comment. As to explaining the differences we see, that is more difficult, especially with the IHME method, since it is so unprincipled. But we have given some comments on the differences.

Related to this, I think a particular extension to the model framework should be the inclusion of seroprevalence data. Representative seroprevalence data has been shown to be highly predictive of excess mortality in many countries (e.g. Ghafari et al 2022. Nat Comms) and would be a valuable covariate to incorporate. I am aware that seroprevalence is more sparse than all cause mortality data, but it could be incorporated in a number of ways. Possibly the simplest would be to infer

the expected seroprevalence nationally/regionally based on reported COVID-19 deaths and an assumed IFR based on IFR by age patterns. The ratio of expected seroprevalence against observed seroprevalence could be incorporated as a time-invariant variable. If multiple seroprevalence data exist, then extensions to acknowledge that COVID-19 death ascertainment may change over time. Regional/urban seroprevalence data could similarly be used when regional/urban data are available.

Alternatively, seroprevalence could be inferred from the predicted excess mortality estimates that have been produced (assuming all excess mortality is COVID-19) and compared against seroprevalence data. And if the only good seroprevalence data is subnational/in cities then making simple assumptions that subnational transmission is comparable to national transmission. This would show that in some EMRO countries, e.g. Somalia, the excess mortality aligns closely with seroprevalence (e.g. in Benadir, Adam et al 2022. Vaccines) but in Syria the estimates appear too low.

This is a good idea, and we will examine the feasibility of using this variable when we next update the estimates, later in the year.

Lastly, there are a number of subnational studies that have produced estimates of changes in mortality during COVID-19 in LICs that could be interesting to consider in Discussion for comparison. E.g. Koum-Besson et al BMJGH 2021, or Watson et al. 2021 Nat Comms, which have mortality data sets, which although not all cause mortality, could be used to produce P-scores to compare against the predictions. (There are more examples, but have just focussed on those I am aware of in EMRO regions).

For this study we have focused on data for which we can rely on a proportionality assumption for the historical subnational and national time series, such that the inference on all-cause mortality in the pandemic period is reliable and defensible. We have not considered non-all-cause data sets missing this criticial piece of information with which to reliable scale observed mortality during the pandemic as the indirect COVID-19 mortality and changes in other causes would be missing.

H. References: Does this manuscript reference previous literature appropriately? If not, what references should be included or excluded?

Overall referencing is good. Discussion could be extended to review the results in the context of other mortality studies (as above) to evaluate the predictions.

The main results of this paper are the estimates of excess at the global and regional level with some country examples. We have referenced the main studies that have done the same i.e. IHME

and the Economist and for select countries such as India for which a number of studies have generated estimates.

I. Clarity and context: Is the abstract clear, accessible? Are abstract, introduction and conclusions appropriate?

Manuscript needs to be re-written to be more succinct - much of the methods are in AAS, and a shorter description could be used in the Methods here.

We have done this.

Reviewer Reports on the First Revision:

Referees' comments:

Referee #1 (Remarks to the Author):

The current version is a great improvement over the previous ones and all of my main concerns have been addressed:
- The Nature submission is well organized, with a clear 'division of labour' between this manuscript and the technical companion paper (Knutson et al. 2022). It flows well and the technical level is uniform throughout the manuscript.
- The Supplement to the Nature submission is informative in its own right, and contains a number of important ramifications (India, Sweden and Germany, ranking) that nevertheless would break the flow of the main paper.
- The methodology is clearly described, at a level accessible to the broad readership of Nature, with a fine logical structure (process / data / statistical methods).
- It is stated several times that a number of choices made will be revisited in the future, e.g., related to Sweden and Germany. The criticisms regarding these countries have been properly addressed.
- The comparison with other leading causes of mortality is very insightful (page 18)
A number of small residual points:
- On page 27 of the main paper: please mention the total number of Indian states in addition to the number of states with sufficient data.
- Page 8, line -9: COVID-19 mortality
- Page 8, line -3: perhaps: "we estimate that more…"
- Page 17, line 18: but as we have
- Page 17, line -1: high. To
- Page 19: "ravaged humanity": could perhaps be rephrased as "severely impacted humanity" or something to that effect
- Table 3 in the Supplement needs a better caption.
- Page 9 in the Supplement contains an unresolved LaTeX reference.
- Figures 5 and 9 in the Supplement contain some very small text.

Geert Molenberghs

Referee #2 (Remarks to the Author):

The authors have properly addressed all the comments and observations I made during the review process. Even if the potential issues with the models' parameterization are still in place, the authors discuss these issues carefully and state that future releases will include adjustments and further sensitivity analyses.

Finally, the changes to the paper structure and content make it more consistent and a better complement to the AAS methodological paper. For instance, the simplification of the description of the methods in this manuscript is appropriate for putting more emphasis on the estimates and

potential implications, which is the aim of this manuscript.

I recommend accepting this paper for publication.

The paper is a valuable contribution to the analysis and assessment of the global mortality burden of the pandemic. The approach, data quality, presentation quality, statistics, and treatment of uncertainties are appropriate. The manuscript is very well written. The conclusions are robust and reliable. The abstract, introduction and conclusions are lucid and appropriate.

Congratulations to the authors for a great job!

Referee #3 (Remarks to the Author):

Thank you to the authors for taking the time to respond to my comments.

In particular, thank you for providing a codebase to go with the manuscript. It is very much appreciated and having gone through it, I am largely satisfied with the code/annotations. I have a few comments though, which should be addressed before publication to ensure reproducibility in the future:

1. A tagged release of the codebase must be conducted so that the analysis the resulted in the first set of WHO excess mortality estimates can be reproduced in the future, given that the team has stated that the analysis will be updated in/every 6 months.

2. There are numerous datasets that are sourced either from dropbox links or local directories not on Github. These must be changed so the analysis is fully reproducible. For example the dropbox files should be downloaded into the repository and read from within, which will protect against either file changes to these datasets or these being removed in the future.

3. For datasets that are imported from external databases (STMF, Economist estimates, WHO estimates), these need to be version tagged in some way. For example, the economist data has had changes to historical estimates as the model is developed. Similarly, country mortality estimates may be historically updated. Without being able to link to a specific version of the data (e.g. Github commit) then the repository is not future proofed for reproducibility. Most simply, downloading these datasets to be saved locally and then reading these data sets in will provide one solution as long as the data/time of the data access is recorded as metadata. Better still would be using specific Github commits where possible.

4. The Dependency requirements for INLA, could you state which version of INLA was used. (Ideally all versions of R packages used should be noted in the repository for reproducibility, simply a saved sessionInfo() object, or better still using the renv package).

Author Rebuttals to First Revision:

• From reviewer 1, we have updated the text for the number of states in India and included the edits supplied for the text. We also fixed the caption and latex reference issues pointed out.

• From reviewer 3. We have updated the github code so that it only draws from locally stored files. We have also added comment with details on the dates of the inputs from the external databases. The version for INLA is declared in the landing page and the codebase itself tagged for this release. Many thanks to the editors and reviewers for all the very constructive comments and feedback that contributed to this final product.
